# Genome-wide scan identifies novel genetic loci regulating salivary metabolite levels

**DOI:** 10.1093/hmg/ddz308

**Published:** 2020-01-21

**Authors:** Abhishek Nag, Yuko Kurushima, Ruth C E Bowyer, Philippa M Wells, Stefan Weiss, Maik Pietzner, Thomas Kocher, Johannes Raffler, Uwe Völker, Massimo Mangino, Timothy D Spector, Michael V Milburn, Gabi Kastenmüller, Robert P Mohney, Karsten Suhre, Cristina Menni, Claire J Steves

**Affiliations:** 1 Department of Twin Research and Genetic Epidemiology, King’s College London, London SE1 7EH, UK; 2 Wellcome Centre for Human Genetics, University of Oxford, Oxford OX3 7BN, UK; 3 Interfaculty Institute of Genetics and Functional Genomics, University Medicine Greifswald & University of Greifswald, 17489 Greifswald, Germany; 4 Institute of Clinical Chemistry and Laboratory Medicine, University Medicine Greifswald, 17489 Greifswald, Germany; 5 Department of Restorative Dentistry, Periodontology, Endodontology, and Preventive and Pediatric Dentistry, University Medicine Greifswald, 17489 Greifswald, Germany; 6 Institute of Bioinformatics and Systems Biology, Helmholtz Zentrum München, German Research Center for Environmental Health, D-85764 Neuherberg, Germany; 7 Discovery and Translational Sciences, Metabolon, Inc., Morrisville, NC 27560, USA; 8 Department of Physiology and Biophysics, Weill Cornell Medicine-Qatar, Doha 24144, Qatar

## Abstract

Saliva, as a biofluid, is inexpensive and non-invasive to obtain, and provides a vital tool to investigate oral health and its interaction with systemic health conditions. There is growing interest in salivary biomarkers for systemic diseases, notably cardiovascular disease. Whereas hundreds of genetic loci have been shown to be involved in the regulation of blood metabolites, leading to significant insights into the pathogenesis of complex human diseases, little is known about the impact of host genetics on salivary metabolites. Here we report the first genome-wide association study exploring 476 salivary metabolites in 1419 subjects from the TwinsUK cohort (discovery phase), followed by replication in the Study of Health in Pomerania (SHIP-2) cohort. A total of 14 distinct locus-metabolite associations were identified in the discovery phase, most of which were replicated in SHIP-2. While only a limited number of the loci that are known to regulate blood metabolites were also associated with salivary metabolites in our study, we identified several novel saliva-specific locus-metabolite associations, including associations for the *AGMAT* (with the metabolites 4-guanidinobutanoate and beta-guanidinopropanoate), *ATP13A5* (with the metabolite creatinine) and *DPYS* (with the metabolites 3-ureidopropionate and 3-ureidoisobutyrate) loci. Our study suggests that there may be regulatory pathways of particular relevance to the salivary metabolome. In addition, some of our findings may have clinical significance, such as the utility of the pyrimidine (uracil) degradation metabolites in predicting 5-fluorouracil toxicity and the role of the agmatine pathway metabolites as biomarkers of oral health.

## Introduction

Metabolic reactions pervade every aspect of human physiology, abnormalities in which underlie a plethora of human diseases (1). Investigating the genetic underpinnings of population-wide variation of metabolites can offer novel insights into human metabolism and diseases, in addition to providing potential therapeutic targets to modulate metabolite levels. Large-scale genetic association studies have so far identified hundreds of loci that regulate the levels of metabolites in blood (2–6) and to a lesser extent in other biospecimens as well (7–9). Previous studies have shown that genetic variants on average explain a greater proportion of trait variance for metabolites compared to what is generally observed for complex traits (3,4), highlighting the utility of metabolites as intermediate traits for dissecting the genetics of complex diseases.

Saliva is an abundantly produced biofluid, and it can be obtained in an inexpensive and non-invasive manner, without the need for healthcare professionals. It is mainly composed of water (>99%) and several other minor constituents such as mucous, digestive enzymes, cytokines, immunoglobulins, antibacterial peptides, and low molecular weight metabolites (10).

Recent advances in metabolomic profiling allow quantification of hundreds of metabolites belonging to diverse biochemical pathways in large population samples (4,11). In 2015, the Human Metabolome Database (HMDB) incorporated data on the ‘salivary metabolome’ which included 853 salivary metabolites that were systematically characterized using a multiplatform approach (12). Since saliva is separated from the systemic circulation by just a thin layer of cells, which allows passive and active exchange of substances (13), it provides a reflection of not just oral health but the functioning of other organ systems as well (14). Indeed, a number of studies have previously reported associations between oral health and systemic conditions such as cardiovascular diseases, diabetes, autoimmune diseases, mental health disorders, and dementia, amongst others (15–19). Therefore, investigation of salivary metabolites could not only provide novel biomarkers but also further our understanding of biological pathways underlying oral as well as general health conditions.

Here we report a genome-wide association analysis (based on 1000 Genomes imputed data) for 476 salivary metabolites in the population-based TwinsUK study, followed by replication in the population-based Study of Health in Pomerania (SHIP-2) cohort.

## Results

### Identification of novel genetic loci regulating salivary metabolite levels

**Figure 1:**
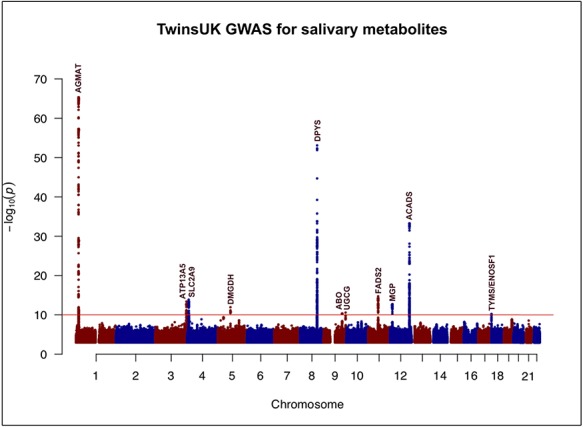
Manhattan plot illustrating the findings of the discovery phase (TwinsUK) of the genome-wide association study for salivary metabolites. The red horizontal line demarcates the study-wide significance threshold of *P* = 10^−10^. Eleven loci surpassed the study-wide significance threshold. The loci are referred to by the name (s) of the overlapping or the nearest gene(s).

Primary genome-wide discovery analysis in TwinsUK identified 13 metabolites that were significantly associated with genetic loci after correcting for multiple testing (*P* < 10^−10^). Furthermore, when we narrowed our analysis to just the loci that were identified in the primary stage, one additional metabolite was found associated (*P* < 10^−6^). Consequently, a total of 14 distinct locus-metabolite associations (hereafter, referred to as ‘mQTLs’) were identified in the discovery phase of our study. The set of significantly associated variants mapped to 11 distinct genetic loci, which have been referred to by the name(s) of the overlapping or the nearest gene(s) (Fig. 1). Three of those loci (*AGMAT, SLC2A9* and *DPYS*) were associated with two metabolites each (Table 1). In all three instances, the two metabolites regulated by the same locus were correlated (Pearson’s *r*^2^ for the metabolite pairs ranged between 0.42 and 0.84) (Supplementary Material, Fig. S1). On the other hand, none of the 14 metabolites that were associated in our study had more than one significant locus. Quantile-quantile (QQ) plots for the significantly associated metabolites are provided in Supplementary Material, Figure S2.

**Table 1:** Summary of genetic loci that were significantly associated with salivary metabolite(s) in the discovery phase (TwinsUK)

Locus	Index variant	Chr	Position (build 37 bp)	Biochemical (metabolite)	EA	EAF	Beta	SE	*P*-value
AGMAT	rs10927806	1	15 909 239	4-Guanidinobutanoate	C	0.45	0.252	0.037	4.4 × 10^−11^
	rs6690813	1	15 861 073	Beta-guanidinopropanoate	C	0.45	0.708	0.034	4.6 × 10^−66^
ATP13A5	rs55918334	3	193 086 314	Creatinine	G	0.53	0.305	0.039	3.9 × 10^−14^
SLC2A9	rs13129697	4	9 926 967	Urate	T	0.72	0.337	0.042	1.3 × 10^−14^
	rs7675964*	4	9 941 434	Allantoin	C	0.73	0.239	0.043	4.1 × 10^−8^
DMGDH	rs248386	5	78 330 227	Dimethylglycine	C	0.81	−0.349	0.048	1.1 × 10^−12^
DPYS	rs80274300	8	105 470 882	3-Ureidopropionate	C	0.81	−0.764	0.043	8.1 × 10^−54^
	rs80274300	8	105 470 882	3-Ureidoisobutyrate	C	0.81	−0.482	0.048	5.8 × 10^−22^
ABO	rs201298979	9	136 145 424	*N*-acetylglucosamine/*N*-acetylgalactosamine	AC	0.77	−0.314	0.046	2.6 × 10^−11^
UGCG	rs10981098	9	114 591 623	Glycosyl-*N*-stearoyl-sphinganine	T	0.79	−0.308	0.046	4.6 × 10^−11^
FADS2	rs174564	11	61 588 305	1-(1-Enyl-palmitoyl)-2-arachidonoyl-GPC	A	0.67	0.324	0.039	1.9 × 10^−15^
ACADS	rs34673751	12	121 171 891	Ethylmalonate	G	0.74	−0.539	0.041	5.1 × 10^−34^
MGP	rs5796614	12	15 053 094	Gamma-carboxyglutamate	G	0.60	−0.290	0.038	1.9 × 10^−13^
TYMS/ENOSF1	rs2790	18	673 086	Ribonate	A	0.81	0.325	0.049	5.8 × 10^−11^

Chr = Chromosome; EA = effect allele; EAF = effect allele frequency; SE = standard error.

For the primary stage of association analysis in the discovery phase (TwinsUK), locus-metabolite associations (mQTLs) were identified using a genome-wide and metabolome-wide significance threshold of *P* < 10^−10^.

^*^This mQTL was identified when the analysis was restricted to only the loci that were identified in the primary stage of association testing (significance threshold of *P* < 10^−6^).

Of the 11 genetic loci that were identified in the discovery phase, four of them (*SLC2A9, DMGDH, FADS2* and *ACADS*) have previously been reported in association with blood metabolites (2,4,5); the remaining seven loci were novel, i.e. they had no previously known associations with metabolites in blood or any other biospecimens. For the genetic loci that were associated with a given metabolite in both saliva and blood, co-localization analysis showed that a common underlying causal variant at the locus possibly regulates the metabolite level in both saliva and blood (*P* < 0.05 for each of the three (out of four) mQTLs for which summary level data were available).

The *AGMAT* locus, one of the novel loci identified, was associated with the metabolites 4-guanidinobutanoate and beta-guanidinopropanoate. These metabolites are generated as intermediate products in the polyamine synthesis pathway, the main site of action for the enzyme agmatinase (encoded by the *AGMAT* gene) (20). The most significantly associated variants for the two metabolites, i.e. rs10927806 and rs6690813, respectively, are in high LD with one another (*r*^2^ = 0.99), suggesting a shared underlying genetic regulation for the two metabolites by the *AGMAT* locus. Similarly, the association between the *ATP13A5* locus and creatinine (a widely used measure of renal function), observed in our study, has also not been reported previously. Another interesting novel association that we identified pertained to the metabolism of the pyrimidine uracil—the *DPYS* locus (encodes for the enzyme dihydropyrimidinase, involved in uracil degradation) was associated with the metabolites 3-ureidopropionate and 3-ureidoisobutyrate (breakdown products of uracil metabolism). The association between the *TYMS/ENOSF1* locus and ribonate is also intriguing, since it has previously been shown that ribonate is one of the substrates for the catalytic activity of reverse thymidylate synthase (rTS), the protein product of *ENOSF1* (21). Therefore, it appears that *ENOSF1*, which is the source of anti-sense RNA of *TYMS,* is probably the functional gene mediating the observed association of the *TYMS/ENOSF1* locus with salivary ribonate*.* The associations of the *SLC2A9* locus with allantoin, the *ABO* locus with *N*-acetylglucosamine/*N*-acetylgalactosamine, the *UGCG* locus with glycosyl-*N*-stearoyl-sphinganine, and the *MGP* locus with gamma-carboxyglutamate were the remaining mQTLs observed in our study that have not been reported previously.

Of the 14 mQTLs that we identified, it was possible to test the most significant variant-metabolite pair for nine mQTLs, each in serum and faecal metabolite data in TwinsUK (metabolites corresponding to the remaining five mQTLs were not present in the serum and faecal metabolite datasets). Of them, associations for the *ATP13A5* and *DPYS* loci did not replicate in serum (*P* > 0.05), while none of the associations, barring the one for the *ACADS* locus, replicated in the faecal data (*P* > 0.05) (Supplementary Material, Table S1). Thus, a comparison across all three biospecimens (for the significantly associated salivary metabolites which were also measured in serum and faecal samples in TwinsUK) demonstrates that, in the TwinsUK dataset, the effects of the *ATP13A5* and *DPYS* loci appear to be specific to saliva (Supplementary Material, Fig. S3).

For none of the mQTLs did we find any additional independent signals at the associated locus after conditioning for the most significant variant (conditional *P* > 10^−5^ for all variants tested at each locus), a finding which was verified by the regional association plots (Supplementary Material, Fig. S4). The observation that a single genetic association signal underlies each of the significantly associated metabolites might partly be due to our lack of power to detect secondary signals at these loci.

The strength of association for the most significant variant-metabolite pair did not change much on adjusting for periodontal disease (PD) status, for any of the mQTLs (Supplementary Material, Table S2). Hence, it does not appear that the condition of oral health, which was ascertained using PD status, has a significant effect on the associations observed in our study. Similarly, the most significant variant-metabolite pair for each of the mQTLs remained significant (*P* > 0.05) on adjusting for either the smoking status, amount of alcohol intake or BMI (Supplementary Material, Table S3).

For 8 of the 11 associated loci, it was observed that the most significant variant demonstrated an eQTL effect on at least one transcript in one of the tissues in the GTEx database (no significant eQTL effects were observed for the *ATP13A5*, *SLCA2A9* and *ABO* loci) (Supplementary Material, Table S4). In case of seven of those eight loci (except *DPYS*), the significant eQTL effect was observed for the overlapping or the nearest gene transcript, and for five of those seven loci (*AGMAT*, *FADS2*, *DMGDH*, *TYMS/ENOSF1* and *UGCG*), the eQTL effect was observed in one of the gut-related tissues. While eQTL data for transcripts assayed in the minor salivary glands were available for a small number of donors (*N* = 97) in the GTEx database, we did not observe significant eQTL effects in the salivary tissue for any of the associated loci.

### Replication of the discovery phase findings

In SHIP-2, we could attempt replication for 9 of the 14 mQTLs that were identified in the discovery phase (metabolites corresponding to the remaining five mQTLs were not measured in SHIP-2). In the initial replication analysis, which was performed using salivary metabolite data that were not normalized for sample osmolality, eight of the nine discovery phase associations were replicated (*P* < 0.05), with the direction of effect consistent with that observed in TwinsUK (Table 2). The association between the *ABO* locus and *N*-acetylglucosamine/*N*-acetylgalactosamine was the only finding from the discovery phase that did not replicate in SHIP-2. When the replication analysis in SHIP-2 was repeated with metabolite data that were normalized for the sample osmolality, the strength of all the associations was comparatively reduced (Supplementary Material, Table S5).

**Table 2:** Summary of the discovery phase associations that were tested in the replication study (SHIP-2)

Locus	Index variant	Chr	Biochemical (metabolite)	EA	Beta	SE	*P*-value
AGMAT	rs10927806	1	4-Guanidinobutanoate	C	0.115	0.044	8.9 × 10^−3^
ATP13A5	rs55918334	3	Creatinine	G	0.239	0.040	2.8 × 10^−9^
SLC2A9	rs13129697	4	Urate	T	0.576	0.045	7.7 × 10^−35^
	rs7675964	4	Allantoin	C	0.209	0.049	2.3 × 10^−5^
DMGDH	rs248386	5	Dimethylglycine	C	−0.336	0.055	1.4 × 10^−9^
DPYS	rs80274300	8	3-Ureidopropionate	C	−1.035	0.049	3.1 × 10^−82^
ABO^a^	rs9411378	9	*N*-acetylglucosamine/*N*-acetylgalactosamine	A	0.032	0.048	0.502
ACADS	rs34673751	12	Ethylmalonate	G	−0.132	0.049	7.3 × 10^−3^
TYMS/ENOSF1	rs2790	18	Ribonate	A	0.137	0.055	0.013

^a^Since information for the index variant (rs201298979) at the *ABO* locus was not available in the replication study, a proxy variant (rs9411378) that was in high LD (*r*^2^ = 0.95) with the index variant was used for the replication analysis.

Replication in SHIP-2 could be attempted for 9 of the 14 mQTLs that were identified in the discovery phase (metabolites corresponding to the remaining five mQTLs were not measured in SHIP-2). For each of these nine mQTLs, the most significant variant-metabolite pair identified in the discovery phase was tested in SHIP-2, using salivary metabolite data that were not normalized for osmolality (since SHIP-2 saliva samples represented stimulated saliva).

### Phenotypic associations for salivary metabolites

The majority of the loci associated with salivary metabolites that were identified in our study have been reported in relation with GWAS traits, inborn errors of metabolism and/or clinically relevant biochemical pathways (Table 3). We further tested the salivary metabolites associated with the *DPYS*, *AGMAT* and *ATP13A5* loci in relation with specific phenotypes using information available in the TwinsUK database (Table 4).

**Table 3:** Annotation of the genetic loci and the metabolites that were significantly associated in the discovery phase

Locus	Biochemical (metabolite)	Metabolite super-pathway^a^	Metabolite sub-pathway^a^	Known GWAS disease associations for the locus^b^	Known OMIM phenotype for the locus^c^	Clinical significance of the locus, the metabolite or the biochemical pathway involved
AGMAT	4-Guanidinobutanoate	Amino acid	Guanidino and acetamido metabolism	Glomerular filtration rate, alcoholic chronic pancreatitis	—	Putrescine, a compound that is generated in the pathway which involves the *AGMAT* gene, i.e. polyamine synthesis pathway, has been implicated in bad breath/oral health (22,23)
	Beta-guanidinopropanoate	Xenobiotics	Plant (food) component			
ATP13A5	Creatinine	Amino acid	Creatine metabolism	—	Kufor–Rakeb syndrome	The *ATP13A5* gene encodes the family of proteins that regulate the activity of HMG-CoA reductase (28), the rate-limiting enzyme in cholesterol synthesis
SLC2A9	Urate	Nucleotide	Hypo(xanthine)/inosine (purine) metabolism	Gout^d^	Renal hypouricemia type 2	The *SLC2A9* gene encodes a carrier protein that is involved in urate transport in the proximal convoluted tubules of kidneys
	Allantoin	Nucleotide	Hypo(xanthine)/inosine (purine) metabolism			
DMGDH	Dimethylglycine	Amino acid	Glycine, serine and threonine metabolism	—	Dimethylglycine dehydrogenase deficiency	The *DMGDH* gene encodes an enzyme (dimethylglycine dehydrogenase), which catalyses the conversion of dimethylglycine to sarcosine
DPYS	3-Ureidopropionate	Nucleotide	Uracil (pyrimidine) metabolism	Paget’s disease	Dihydropyrimidinuria	Variants in the *DPYS* gene, which encodes an enzyme in the pyrimidine degradation pathway, have been associated with 5-fluorouracil toxicity (30)
	3-Ureidoisobutyrate	Nucleotide	Uracil (pyrimidine) metabolism			
ABO	*N*-acetylglucosamine/*N*-acetylgalactosamine	Carbohydrate	Amino sugar metabolism	Gastric carcinoma, stroke, venous thromboembolism,^d^ ovarian cancer, malaria, blood cholesterol level, type 2 diabetes	—	The *ABO* gene encodes a protein with glycosyltransferase activity, which forms the basis of the ABO blood group system
UGCG	Glycosyl-*N*-stearoyl-sphinganine	Lipid	Ceramide metabolism	—	—	The *UGCG* gene is involved in glycosphingolipid synthesis, defects in which are known to cause Gaucher’s disease (51), a lysosomal storage disorder
FADS2	1-(1-Enyl-palmitoyl)-2-arachidonoyl-GPC	Lipid	Plasmalogen	ADHD, blood cholesterol level	—	The *FADS2* gene encodes an enzyme (fatty acid desaturase), which catalyses the rate-limiting step in the desaturation of polyunsaturated fatty acids (PUFA)
ACADS	Ethylmalonate	Amino acid	Leucine, isoleucine and valine metabolism	—	Ethylmalonic aciduria	The *ACADS* gene encodes the acyl-CoA dehydrogenase enzyme, which catalyses the initial step in the fatty acid of oxidation pathway
MGP	Gamma-carboxyglutamate	Amino acid	Glutamate metabolism	—	Keutel syndrome	The *MGP* gene, a regulator of physiologic tissue calcification, is associated with calcification of vasculature in patients with cardiovascular disease (40). The *MGP* gene has also been associated with natural tooth loss in elderly women (52)
TYMS/ENOSF1	Ribonate	Carbohydrate	Pentose metabolism	Hypertension	—	The *TYMS* gene encodes the enzyme thymidylate synthase, the main site of action of 5-fluorouracil. The *ENOSF1* gene encodes the antisense RNA of *TYMS*

^a^Obtained from the KEGG database.

^b^Accessed from the NHGRI GWAS catalogue.

^c^Accessed from the OMIM database.

The genetic loci that were identified in the discovery phase were annotated for known disease associations using the NHGRI GWAS and OMIM databases. Similarly, the metabolites identified in the discovery phase were annotated for the associated biochemical pathway.

^d^Strong evidence for co-localization of the association signal for the trait with that for the metabolite which was associated with the same locus.

**Table 4:** Summary of the phenotypes that were tested in relation with specific salivary metabolites

Locus	Chr	Biochemical (metabolite)	Phenotypic association(s) tested
AGMAT	1	4-Guanidinobutanoate	1. Periodontal disease
		Beta-guanidinopropanoate	2. Clinical depression or anxiety disorder
ATP13A5	3	Creatinine	1. eGFR (measure of renal function)
			2. Grip strength (measure of muscle strength)
			3. Statin usage
DPYS	8	3-Ureidopropionate	1. Irritable bowel syndrome (based on the ROME-III criteria)
		3-Ureidoisobutyrate	

eGFR = effective glomerular filtration rate.

The metabolites that were uniquely associated in saliva, i.e. the ones for which a genetic association had not been previously reported in blood, were tested with phenotypes (diseases/traits/adverse drug effects) relating to the metabolite or its associated biochemical pathway, using information from the TwinsUK database.

The *AGMAT-*associated metabolites (4-guanidinobutanoate and beta-guanidinopropanoate) are generated in the polyamine synthesis pathway (https://www.genome.jp/kegg-bin/show_pathway?hsa00330). This pathway also produces the compound putrescine, which has been implicated in poor oral health and foul breath (22,23). Consequently, we evaluated the significance of the *AGMAT-*associated metabolites in oral health by testing them with PD status—the levels of both 4-guanidinobutanoate and beta-guanidinopropanoate were significantly higher in PD cases compared to controls (*P* = 0.0003 and *P* = 0.0006, respectively).

Since eGFR (estimated glomerular filtration rate) is calculated on the basis of serum creatinine, and these two commonly used measures of renal function are negatively correlated, we wanted to investigate the association between salivary creatinine and eGFR. We observed a similar strong negative relationship between salivary creatinine and eGFR (*P* = 1.6 × 10^−11^), which is indicative of a homeostasis between creatinine concentrations in serum and saliva. Furthermore, creatinine is also known to be a marker of muscle mass and strength (24). We, therefore, tested salivary creatinine in relation with grip strength (a measure of muscle strength), which suggested a positive correlation between them (*P* = 0.01).

Apart from these findings, the remaining phenotypic associations that we tested were largely negative, as follows:

The enzyme agmatinase (encoded by the *AGMAT* gene), which acts on the substrate agmatine, has been implicated in the pathophysiology of mood disorders (25). Moreover, studies have also proposed agmatine as a novel neuromodulator (26). We, therefore, tested the *AGMAT-*associated metabolites in relation with a diagnosis of clinical depression or anxiety disorder, and responses (on a ordinal scale) to questions in the Hospital Anxiety and Depression Scale (HADS) questionnaire (27). But, neither analysis showed any significant associations (Supplementary Material, Table S6).

*ATP13A5*, the locus that was associated with salivary creatinine, belongs to the family of ATPases that regulate the activity of HMG-CoA reductase (28), the main site of action of the cholesterol-lowering class of drugs called statins. Statins are known to cause muscle dysfunction (myopathy) in a small fraction of patients (29). Given that *ATP13A5* and statins both act in the same biochemical pathway, we assessed whether salivary creatinine is also associated with statin usage, and could therefore be used as a biomarker for statin-induced myopathy. There was, however, no association between salivary creatinine and statin usage (*P* = 0.22).

5-Fluorouracil (5-FU) is a pyrimidine analogue that is a commonly used anticancer drug. It is eliminated from the body via the pyrimidine degradation pathway, and hence, variants in genes coding for the pyrimidine degradation enzymes (for instance, *DPYS*) are known to be associated with the development of 5-FU toxicity (30), which mainly manifests as gastrointestinal side effects. Since we could not directly assess the *DPYS*-associated metabolites (3-ureidopropionate and 3-ureidoisobutyrate) in relation to the gastrointestinal side effects of 5-FU toxicity, we instead used a commonly observed phenotype of gastrointestinal dysfunction, irritable bowel syndrome or IBS (ascertained using the ROME-III questionnaire (31)). For both metabolites, we observed that the levels were not significantly different in IBS ‘cases’ compared to ‘controls’ (*P* > 0.05, for both metabolites). However, this negative finding does not negate the possibility that these metabolites could be of clinical use in predicting 5-FU toxicity.

## Discussion

Here we report a genome-wide association analysis of 476 metabolites measured in saliva samples of healthy population-based studies of European descent. We identified a total of 11 distinct genetic loci that regulate the level of 14 salivary metabolites, of which three loci were associated with more than one metabolite each.

The fact that saliva is reflective of the concentration of biochemicals in blood forms the basis for certain clinical applications of saliva that others have proposed previously such as therapeutic monitoring of drugs (32), cortisol measurement (33) and renal function monitoring (34). Using salivary metabolite data, we replicated associations for certain well-established genetic loci that are known to regulate the level of blood metabolites. Thus, our findings add further credence to the notion that, as biofluids, a certain degree of homeostasis exists between blood and saliva.

Additionally, we identified some novel associations in our study, which have expanded our knowledge of genetic influences on human metabolites. In particular, the association between the *DPYS* locus and pyrimidine metabolites is intriguing because of its clinical relevance. Mutations in the pyrimidine catabolism genes such as *DPYD* (encodes dihydropyrimidine dehydrogenase) and *DPYS* (encodes dihydropyrimidinase) have been linked to inborn errors of metabolism (35,36) as well as development of severe toxicity to the chemotherapeutic agent 5-FU (30,37,38). Studies have previously demonstrated the applicability of salivary measurement of certain pyrimidine pathway metabolites (uracil and dihydrouracil) for evaluating 5-FU toxicity due to deficient *DPYD* activity (39). In our study, variants in the *DPYS* gene correlated with the levels of specific pyrimidine metabolites (3-ureidopropionate and 3-ureidoisobutyrate). Therefore, studies to explore the utility of salivary measurements of these metabolites as non-invasive tools for predicting 5-FU toxicity resulting from mutations that affect *DPYS* activity are warranted. While we could not test 3-ureidopropionate and 3-ureidoisobutyrate in relation to the gastrointestinal side effects of 5-FU toxicity, we did not find any association between these metabolites and a phenotype relevant to gut dysfunction (IBS phenotype).

The other novel finding of note was the association between the *AGMAT* locus and the metabolites 4-guanidinobutanoate and beta-guanidinopropanoate. The *AGMAT*-associated metabolites are produced in the polyamine synthesis pathway, which also generates the compound putrescine that has been implicated in oral health. Moreover, we found that the *AGMAT*-associated metabolites were correlated with PD status, a disease related to poor oral health. Together, these findings suggest that the *AGMAT*-associated metabolites might serve as potential biomarkers for oral health. On the other hand, though the agmatine pathway has been previously implicated in mood disorders, we did not find any association between the *AGMAT*-associated metabolites and either symptoms of or a prior diagnosis of clinical depression or anxiety disorder. Thus, evidence based on the *AGMAT*-associated metabolites does not lend support to the hypothesis regarding a potential link between pathways involved in maintaining oral health and regulation of mood (15,18).

The association between the *ATP13A5* locus and salivary creatinine was also noteworthy. While we observed a degree of homeostasis between salivary and serum creatinine (a commonly used measure of renal function), we did not find any evidence to support our hypothesis of salivary creatinine as a marker for statin-induced myopathy. There were, however, a few other novel associations of interest in our study, such as the one between the *MGP* locus (known to cause abnormal vascular calcification in patients with cardiovascular disease (40)) and gamma-carboxyglutamate, for which we could not assess the clinical significance since specific phenotypic information was not available in sufficient numbers in those with salivary metabolite data.

While we cannot be certain that the novel genetic associations which we identified for salivary metabolites relate to the salivary metabolome alone, we were intrigued to find that these genetic loci had not been reported in association with blood metabolites. This suggests that there may be regulatory pathways of particular relevance to the salivary metabolome. In most cases, the gene transcript nearest to or overlapping our novel genetic loci was expressed in one or more gut-related tissues, including salivary glands. However, for these loci, we did not find much evidence for cis-eQTL effects specific to salivary or other gut tissues, whereby we could attribute their association with the respective salivary metabolite(s) to transcriptional regulation of overlapping/neighbouring genes.

Interestingly, there is growing evidence to suggest that the human metabolome is a reflection of an interaction between the host and the gut microbiome (7,41,42). In the case of the salivary metabolome, this can be explored by testing the association of the both the compositional and functional attributes of the salivary metagenome with salivary metabolites. Studies investigating the gut microbiome have shown a relatively low overall influence of host genetics on microbiome composition, but some key taxa have significant heritability (43). If this is recapitulated in the salivary microbiome, it is possible that the observed associations between genetic loci and salivary metabolites could be mediated by the microbiome. Thus, in future studies with both salivary microbial and metabolite data, it will be worth investigating whether the salivary metabolites that were associated in our study correlate with the composition of the salivary microbiome.

In summary, our study has provided a map of the genetic loci that influence the salivary metabolome, thus offering insights into hitherto unknown biological pathways involved in the regulation of salivary metabolites. Based on what has been observed for other complex human traits, future studies with larger sample sizes are expected to uncover additional genetic loci with much smaller effects on salivary metabolites. While oral health has been implicated in systemic conditions such as cardiovascular diseases and mental health disorders, the exact mechanisms underlying these associations are far from understood. Our study identified the potential clinical relevance for a few salivary metabolites of interest, such as the utility of the pyrimidine (uracil) degradation metabolites in predicting 5-fluorouracil toxicity and the role of the agmatine pathway metabolites as biomarkers of oral health. However, realizing the potential application of salivary metabolites as biomarkers of systemic health conditions would require conducting a more comprehensive analysis of the salivary metabolome with a wider range of phenotypic domains.

## Materials and Methods

### Discovery phase

*Study population*. The discovery phase of the study was conducted in the TwinsUK cohort, an adult twin registry comprising healthy volunteers, based at St. Thomas’ Hospital in London (44). Twins gave fully informed consent under a protocol reviewed by the St. Thomas’ Hospital Local Research Ethics Committee. Subjects of European ancestry with available genotype data and for whom salivary metabolite profiling was done on a fasting state sample were included in our study (*N* = 1419; mean age = 62.2 years; % females = 92.7).

*Genotyping, imputation and QC*. Subjects were genotyped in two different batches of approximately the same size, using two genotyping platforms from Illumina: 300K Duo and HumanHap610-Quad arrays. Whole genome imputation of the genotypes was performed using the 1000 genomes reference haplotypes (45), further details of which are provided in Moayyeri *et al.* (44). Stringent QC measures, including minimum genotyping success rate (>95%), Hardy–Weinberg equilibrium (*P* > 10^−6^), minimum MAF (>0.5%) and imputation quality score (INFO > 0.5), retained ~9.6 million variants for genome-wide analysis.

*Saliva sample collection*. Saliva samples were obtained by asking the fasted volunteer to spit as much saliva as possible into an empty sterile pot over a period of 10 min. The saliva samples were immediately refrigerated and then frozen at −80°C (usually within 4 h of sample collection) before further processing. Following that, the samples were shipped on dry ice for metabolite profiling at Metabolon Inc., Durham, USA (see Supplementary methods (I) for further details on sample processing).

*Metabolic profiling of saliva samples*. Metabolite concentrations in the saliva samples were estimated using Ultrahigh Performance Liquid Chromatography-Tandem Mass Spectroscopy (UPLC-MS) i.e. chromatographic separation, followed by full-scan mass spectroscopy, to record all detectable ions in the samples (see Supplementary methods (II) for further details). Based on their unique ion signatures (chromatographic and mass spectral), 997 distinct metabolites were identified, of which 823 had known chemical identity at the time of analysis. The 823 known metabolites were broadly classified into eight metabolic groups (amino acids, peptides, carbohydrates, energy, lipids, nucleotides, cofactors and vitamins, and xenobiotics) as described in the KEGG (kyoto encyclopedia of genes and genomes) database (46). The eight metabolic groups were further subdivided into 99 distinct biochemical pathways.

Raw metabolite values were normalized for the volume and osmolality measurement of the saliva samples. The normalized metabolite values were then log-transformed, and scaled to uniform mean 0 and standard deviation 1. Of the 823 known metabolites, 476 were retained for analysis based on the presence of measurement in more than 80% samples. For these metabolites, we imputed missing data using the run day minimum value for the metabolite, based on the rationale that missing values represented metabolite concentrations that were too low to be detected. The resulting imputed dataset of the 476 metabolites was used for further analysis.

*Genome-wide association analysis of salivary metabolites*:

(i) Primary genome-wide association analysis

For each of the 476 metabolites, a linear mixed-model was fitted to test the association between the metabolite (dependent variable) and genome-wide variants (independent variable). Age, sex and time of saliva sample collection were included as covariates in the model. The score test implemented in GEMMA (47), which utilizes a sample kinship matrix (estimated using a subset of ~500 000 variants) to account for the twin structure or relatedness in the TwinsUK data, was used to assess significance of the associations. A genome-wide and metabolome-wide significance cut-off of *P* < 10^−10^ (corresponding to the conventional genome-wide significance threshold of 5 × 10^−8^, corrected for 476 metabolites) was used to identify significant variant-metabolite associations. For each locus that was significantly associated with a metabolite, we reported the variant with the lowest association *P*-value.

(ii) Testing loci identified in the primary analysis for additional metabolite associations

Next, we focused just on the loci that were identified in the primary stage of association testing to look for additional variant-metabolite associations for those loci. For each locus that was identified in the primary analysis, we clumped all variants located within a 100 Mb block and with LD (*r*^2^) > 0.2, to check for additional metabolite associations at a significance threshold of *P* < 10^−6^ (corresponding to *P* = 0.05, corrected for 476 metabolites and a prior assumption of about 100 associated loci).

(iii) Testing the significantly associated loci using metabolite data from other biospecimens

For each associated locus, we further assessed the most significant variant-metabolite pair by using measurements for the respective metabolite in serum (4) and faecal samples (7) of the TwinsUK subjects (provided the metabolite was measured in that biospecimen). We tested only those serum and faecal samples that overlapped with the ones in saliva and were collected within 5 years of the saliva samples (in order to ensure that, for a given individual, samples from the different biospecimens being tested were obtained within a certain period of one another). Association testing for serum and faecal metabolites was done using an identical model to that described for the analysis of salivary metabolites.

In addition, for the locus-metabolite associations that have been previously reported for blood metabolites, we performed co-localization analysis using the GSMR/HEIDI method (implemented in GCTA) (48) in order to test whether the associations in saliva and blood were mediated by a common underlying signal (pleiotropic effect) or distinct signals at the locus. We used the metabolomic GWAS summary level data made available by Shin *et al.* (4) and Long *et al.* (5) for the co-localization analysis.

(iv) Conditional analysis for the significantly associated loci
  (a) Detection of secondary association signals

We used approximate conditional analysis, as implemented in GCTA (49), to test whether any of the associated loci had multiple distinct, i.e. secondary association signals, at a ‘locus-wide’ significance threshold of *P* < 10^−5^. For each associated locus, all variants that surpassed the study-wide significance threshold (*P* < 10^−10^) were conditioned on the most significantly associated variant at that locus (using the association summary statistics). For the conditional analysis, we used genotype data from the complete TwinsUK dataset (*N* = 5654) to model LD patterns between variants.

(b) Adjustment for factors known to affect salivary metabolite levels

Since the condition of oral health is known to affect salivary metabolite levels (11), we adjusted the most significant variant-metabolite pair for each locus-metabolite association for a measure of oral health that was available in the TwinsUK dataset, i.e. periodontal disease (PD) status. Self-reported gingival bleeding and a history of gum disease or tooth mobility were used as indicators of PD in TwinsUK (50) (270 PD cases; 1083 controls).

Similarly, we also tested the effect of other factors that are known to affect salivary metabolite levels, such as smoking, alcohol consumption and BMI on the significant locus-metabolite associations.

*Expression quantitative trait locus (eQTL) analysis*. We used the version 7 data release of the Genotype-Tissue Expression (GTEx) project (accessed 15 April 2019), which was based on RNA-Seq data obtained from 48 non-diseased tissue sites across ~1000 individuals, to test whether the most significant variant at each associated locus had an eQTL effect on transcripts located within a 1 Mb window of the variant.

*Annotation of associations using reference databases*. We searched the NHGRI GWAS catalogue (accessed 15 April 2019) for previous disease associations for the significantly associated loci that were identified in our study. For the loci that were previously associated with other GWAS traits, we performed co-localization analysis using the GSMR/HEIDI method (implemented in GCTA) (48) in order to test whether the associations were mediated by a common underlying signal (pleiotropic effect) or distinct signals at the locus. We used publicly available GWAS summary level data for the co-localization analysis.

We also searched the OMIM database (accessed 15 April 2019) to check the candidate genes at the associated loci for a causal link with inborn errors of metabolism.

Moreover, we also queried the HMDB (12) and KEGG (46) databases to identify biochemical pathways and known disease associations for the associated metabolites.

### Replication phase

The replication phase was performed in the Study of Health in Pomerania (SHIP-2), a population-based study comprising European ancestry subjects, conducted in the northeastern area of Germany. Further details of SHIP-2, including cohort details, genotyping and imputation, and saliva sample collection are provided in Supplementary methods (III). Metabolic profiling for SHIP-2 saliva samples (*N* = 1000) was performed using an identical process to that described for TwinsUK.

Since the method of saliva sample collection in SHIP-2 (chewing on a piece of cotton) meant that the sample thus obtained represented stimulated saliva, normalization of the metabolite measurements for sample osmolality was not considered necessary. The fact that the salivary osmolality values in SHIP-2 had a much narrower distribution compared to that in TwinsUK verified our rationale (Supplementary Material, Fig. S5).

For each locus-metabolite association that was identified in the discovery phase, we tested the most significantly associated variant using a linear regression model that was fitted on R (version 3.5.2). Covariates used in the association model were similar to those used for the discovery phase.

### Testing the significantly associated salivary metabolites with phenotypes of interest

We wanted to test how salivary metabolites that were regulated by genetic loci related to relevant phenotypes. For that, we selected the metabolites that were uniquely associated in saliva, i.e. the ones for which a genetic association had not been previously reported in blood, and tested them with phenotypes (diseases/traits/adverse drug effects) relating to the metabolite or its associated biochemical pathway. We obtained the relevant phenotype information from the TwinsUK database, selecting one twin per pair (*N* = 1426). The phenotype association analysis was performed on R (version 3.5.2) by fitting a linear regression model to test the association between the salivary metabolite and the disease/trait/adverse drug effect (adjusted for age and sex).

## Web resources

1000 Genomes project: http://www.internationalgenome.org/

Metabolon: https://www.metabolon.com/

GEMMA: http://www.xzlab.org/software.html

KEGG: https://www.genome.jp/kegg/

GCTA: http://cnsgenomics.com/software/gcta/

GTEx: https://gtexportal.org/home/

NHGRI GWAS catalogue: https://www.ebi.ac.uk/gwas/

OMIM: http://omim.org/

HMDB: http://www.hmdb.ca/

LocusZoom: http://locuszoom.org/

UK Biobank GWAS summary data: http://www.nealelab.is/uk-biobank

Serum metabolomic GWAS summary data: http://metabolomics.helmholtzmuenchen.de/gwas/; http://www.hli-opendata.com/Metabolome/

## Supplementary Material

FigureS1_ddz308

FigureS2_ddz308

FigureS3_ddz308

FigureS4_ddz308

FigureS5_ddz308

FigureS6_ddz308

Supplementary_material_ddz308

TableS3_ddz308

TableS4_ddz308
