# Supplementary material for: Genome-wide scan identifies novel genetic loci regulating salivary metabolite levels
Source: Hum Mol Genet. 2020 Jan 21;29(5):864–75. doi: 10.1093/hmg/ddz308 (PMC7104674; doi:10.1093/hmg/ddz308)

(i) beta-guanidinopropanoate and 4-guanidinobutanoate

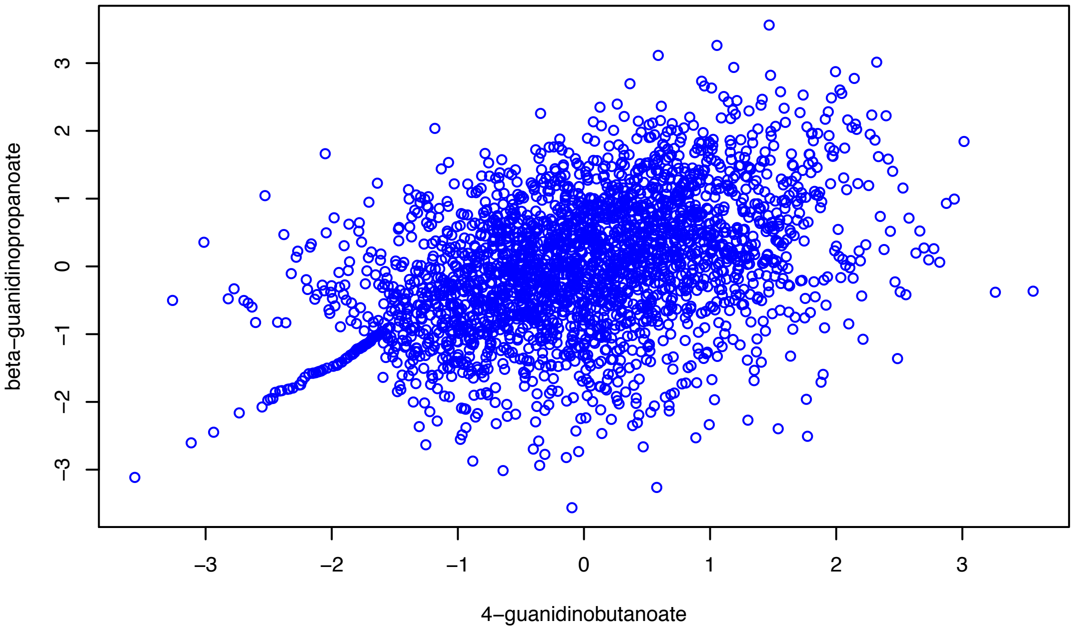

(ii) urate and allantoin

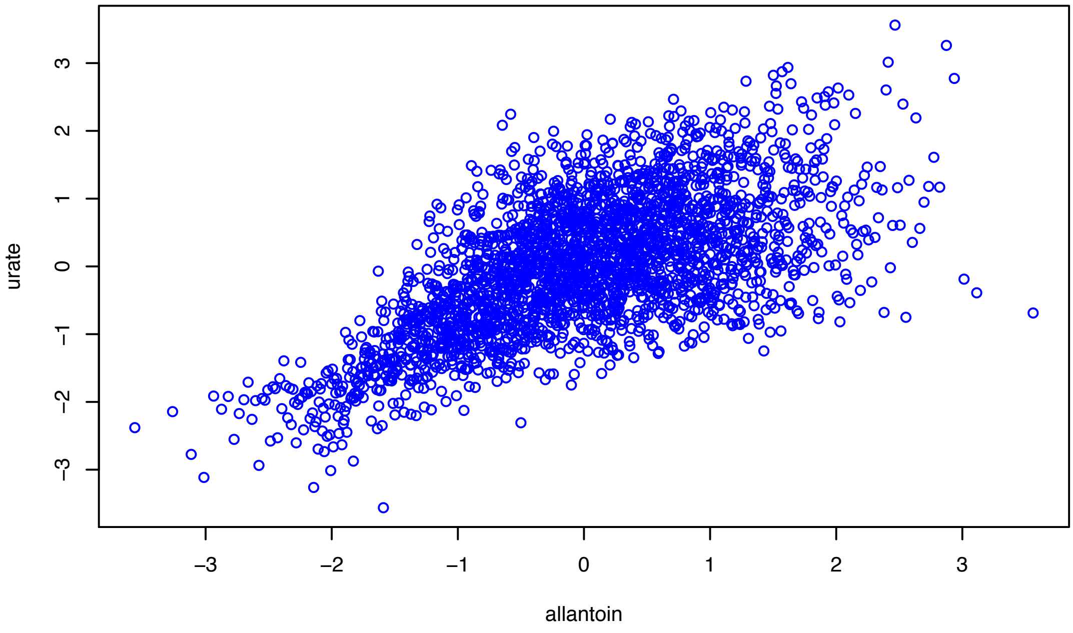

(iii) 3-ureidopropionate and 3-ureidoisobutyrate

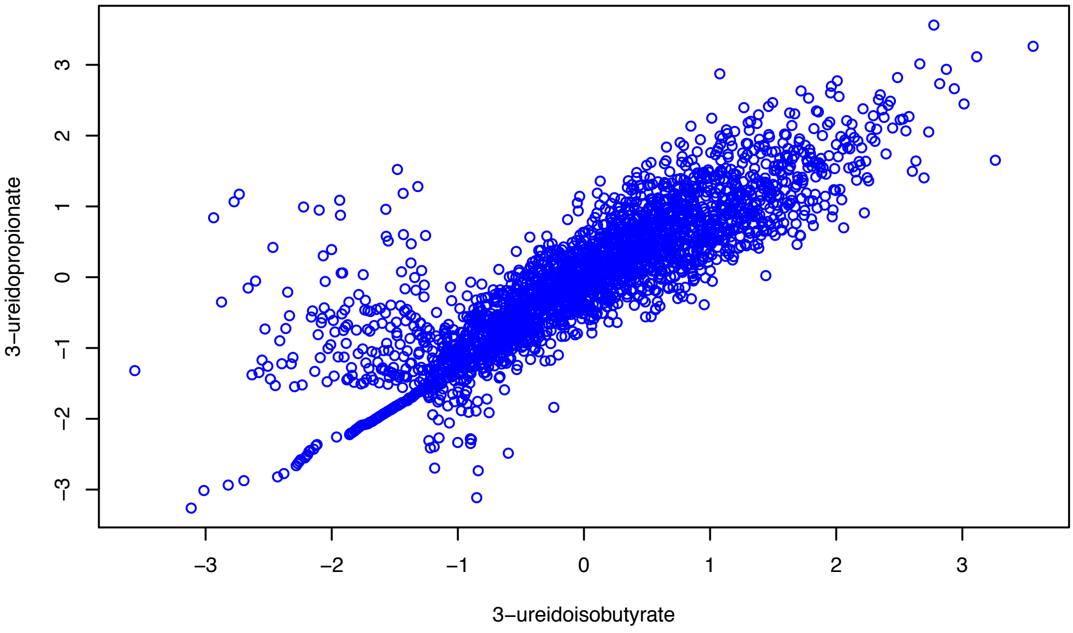

Supplement: FigureS1_ddz308 [file figures1_ddz308.pdf]
