# Supplementary material for: Genome-wide scan identifies novel genetic loci regulating salivary metabolite levels
Source: Hum Mol Genet. 2020 Jan 21;29(5):864–75. doi: 10.1093/hmg/ddz308 (PMC7104674; doi:10.1093/hmg/ddz308)

(i) 4-guanidinobutanoate

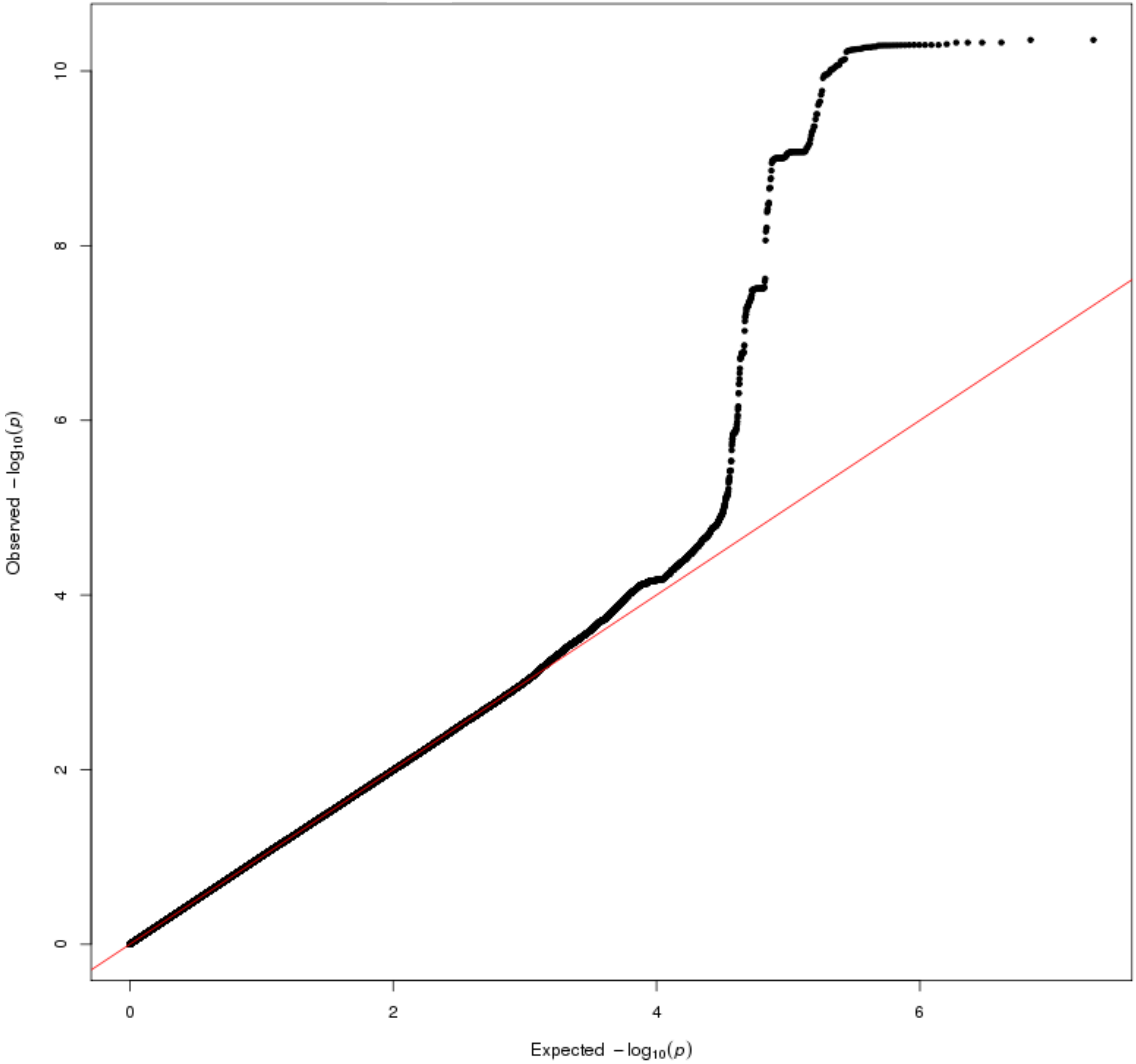

(ii) beta-guanidinopropanoate

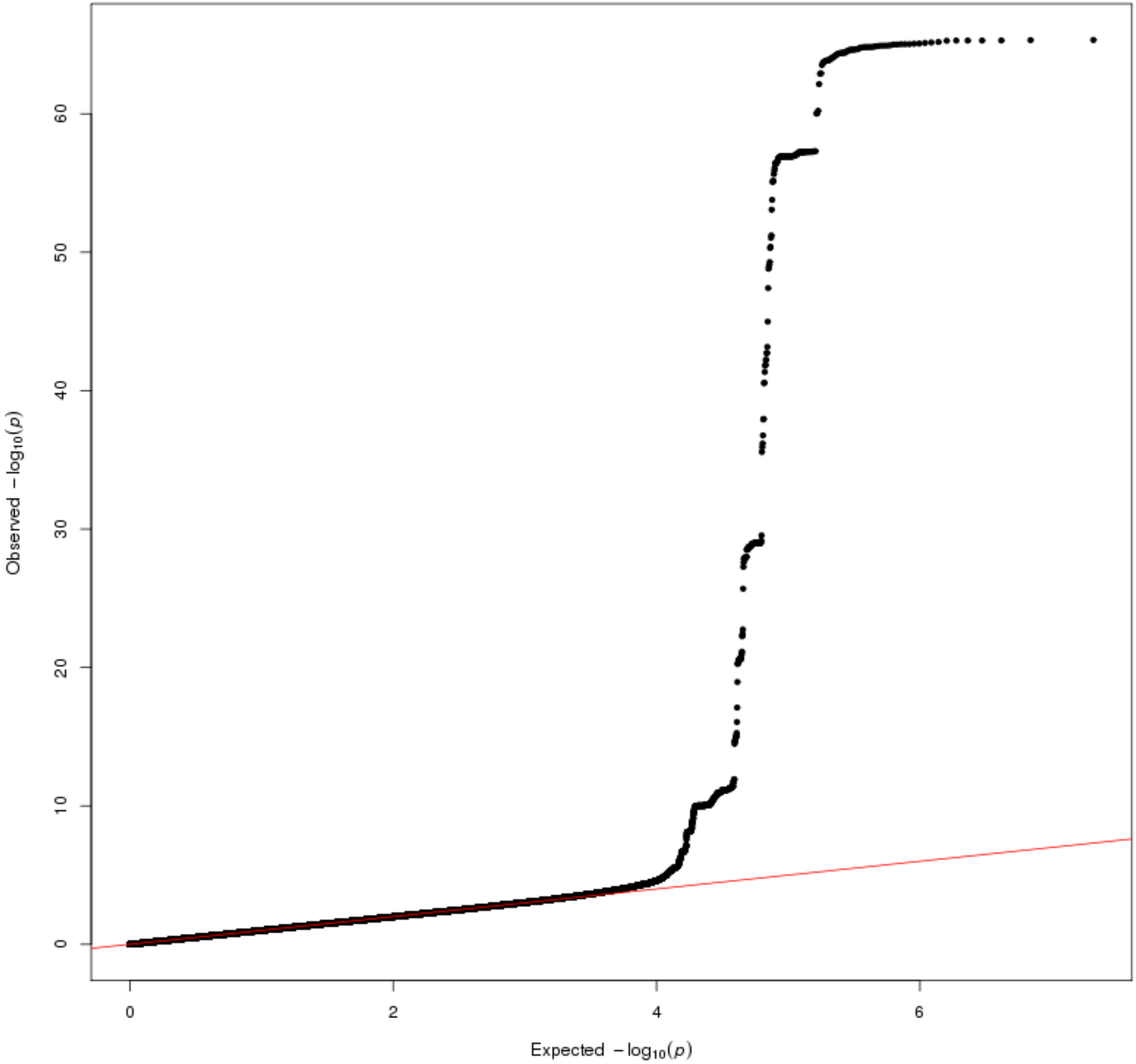

(iii) creatinine

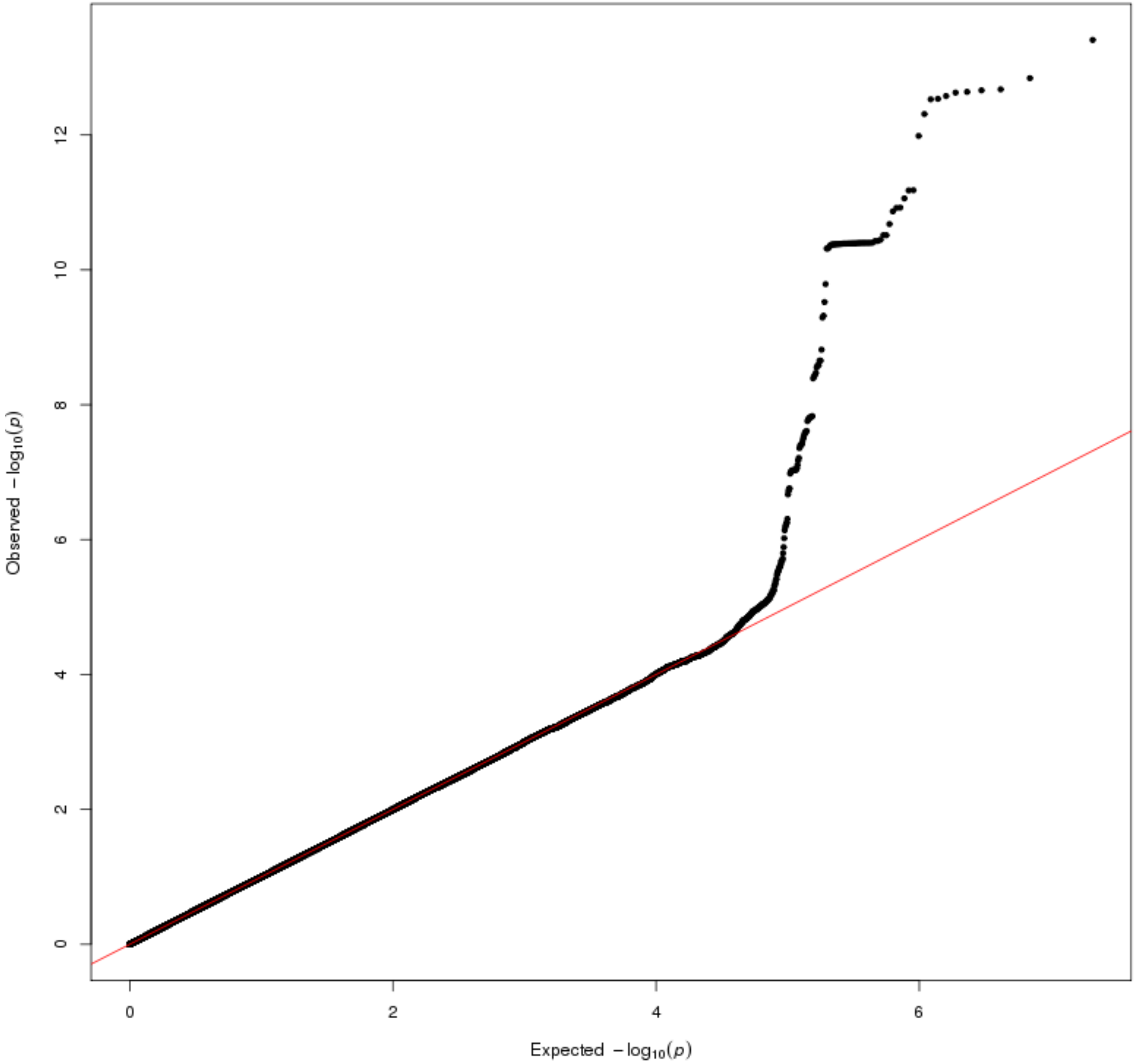

(iv) urate

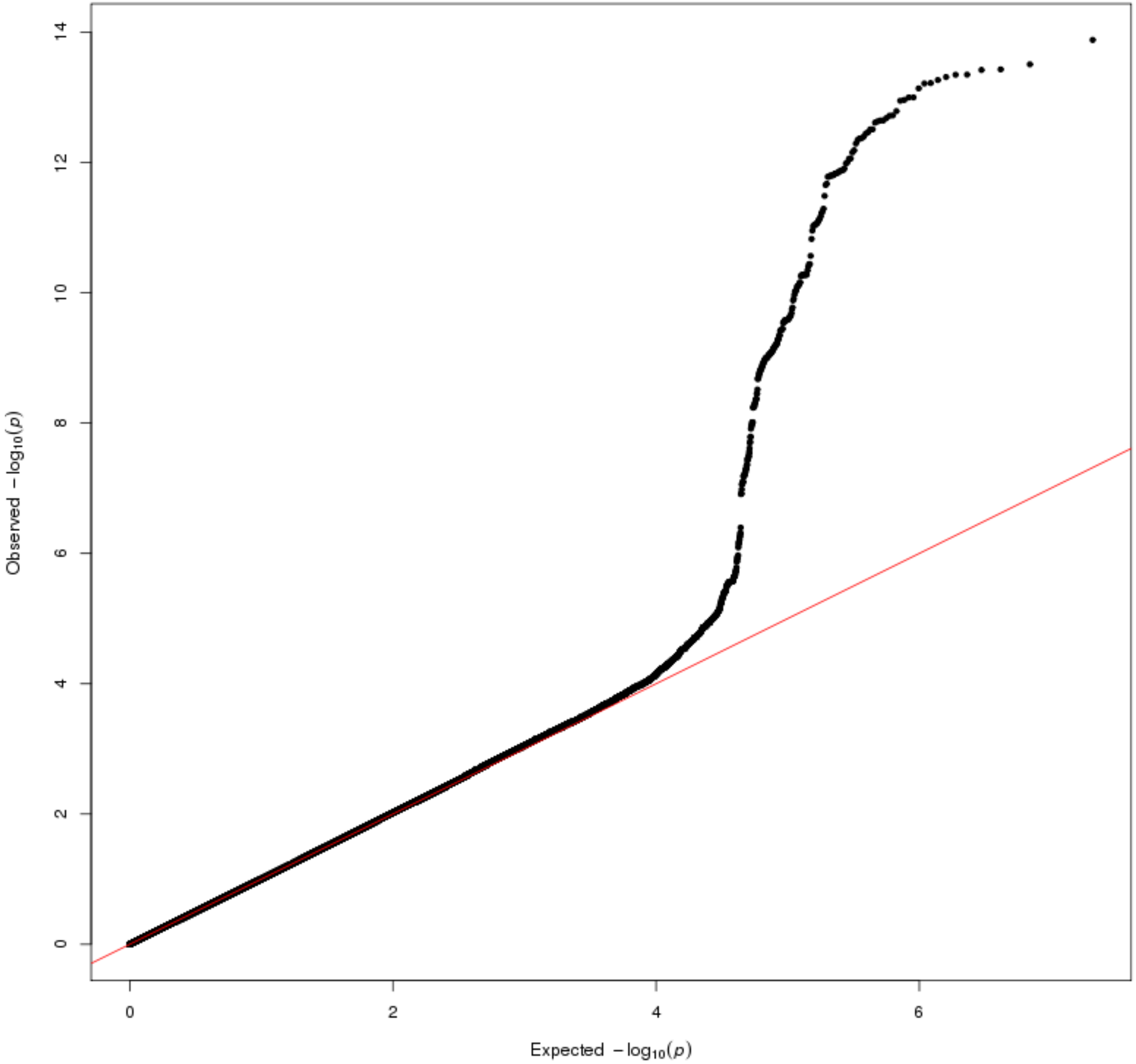

(v) allantoin

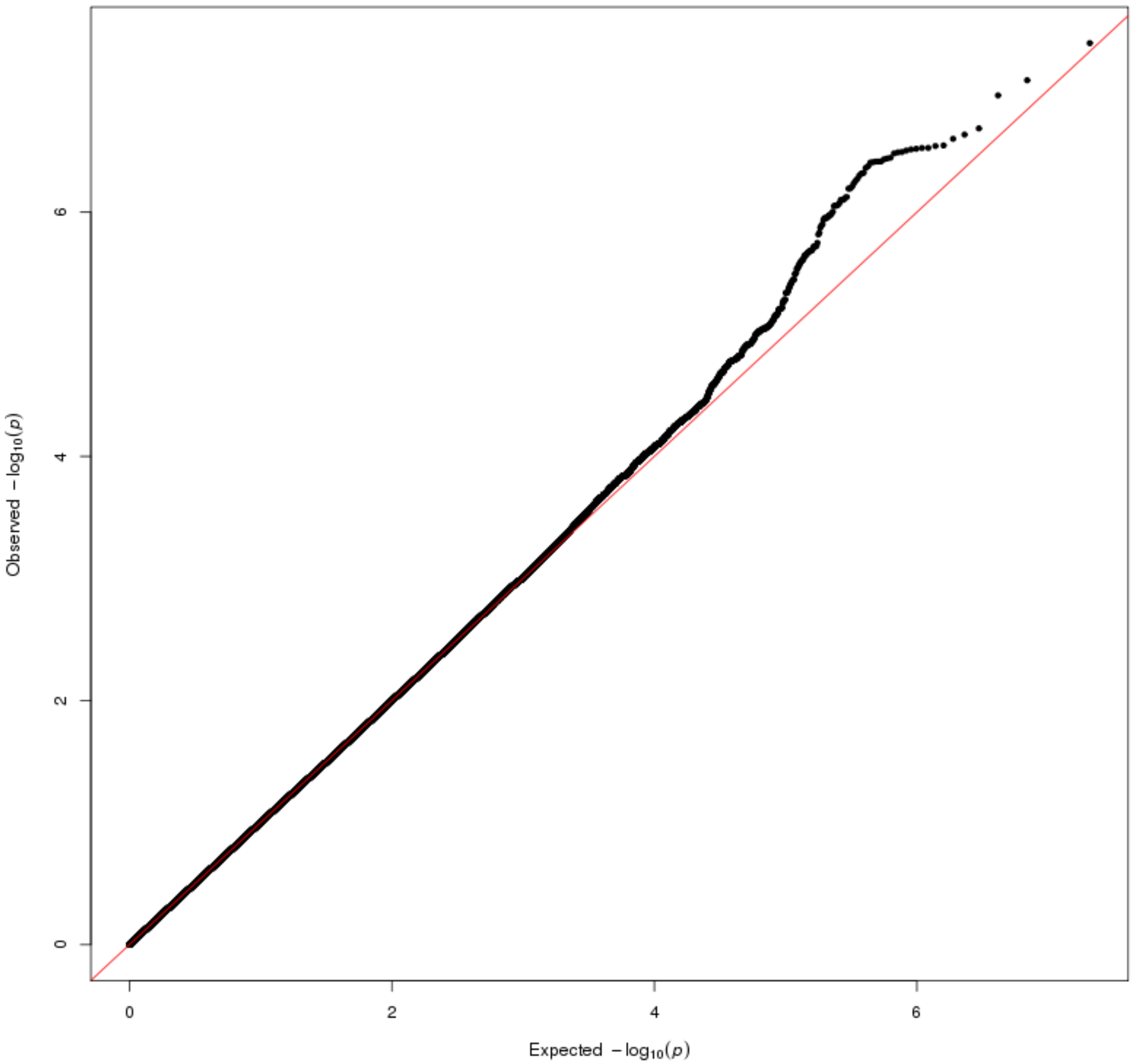

(vi) dimethylglycine

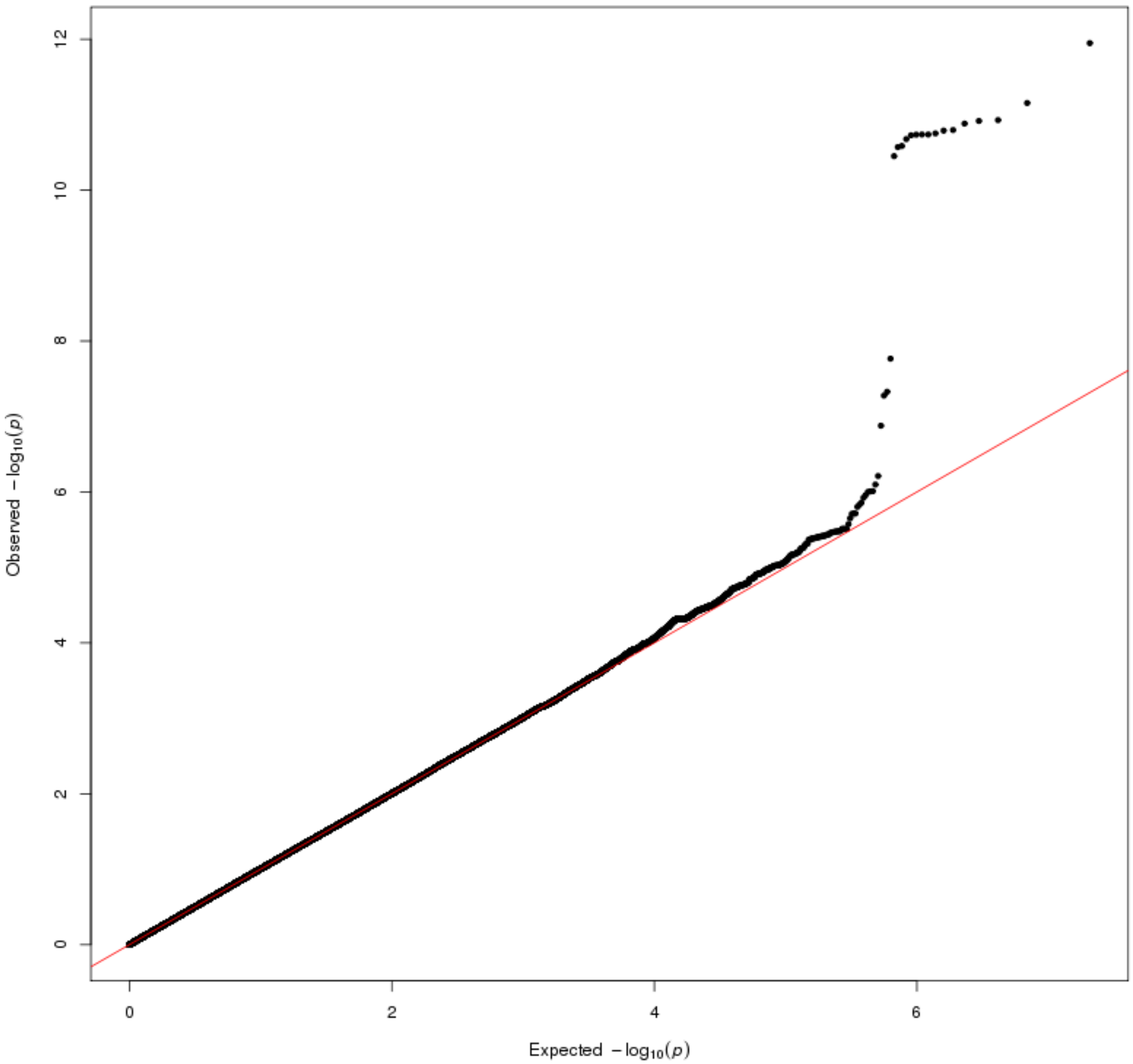

(vii) 3-ureidopropionate

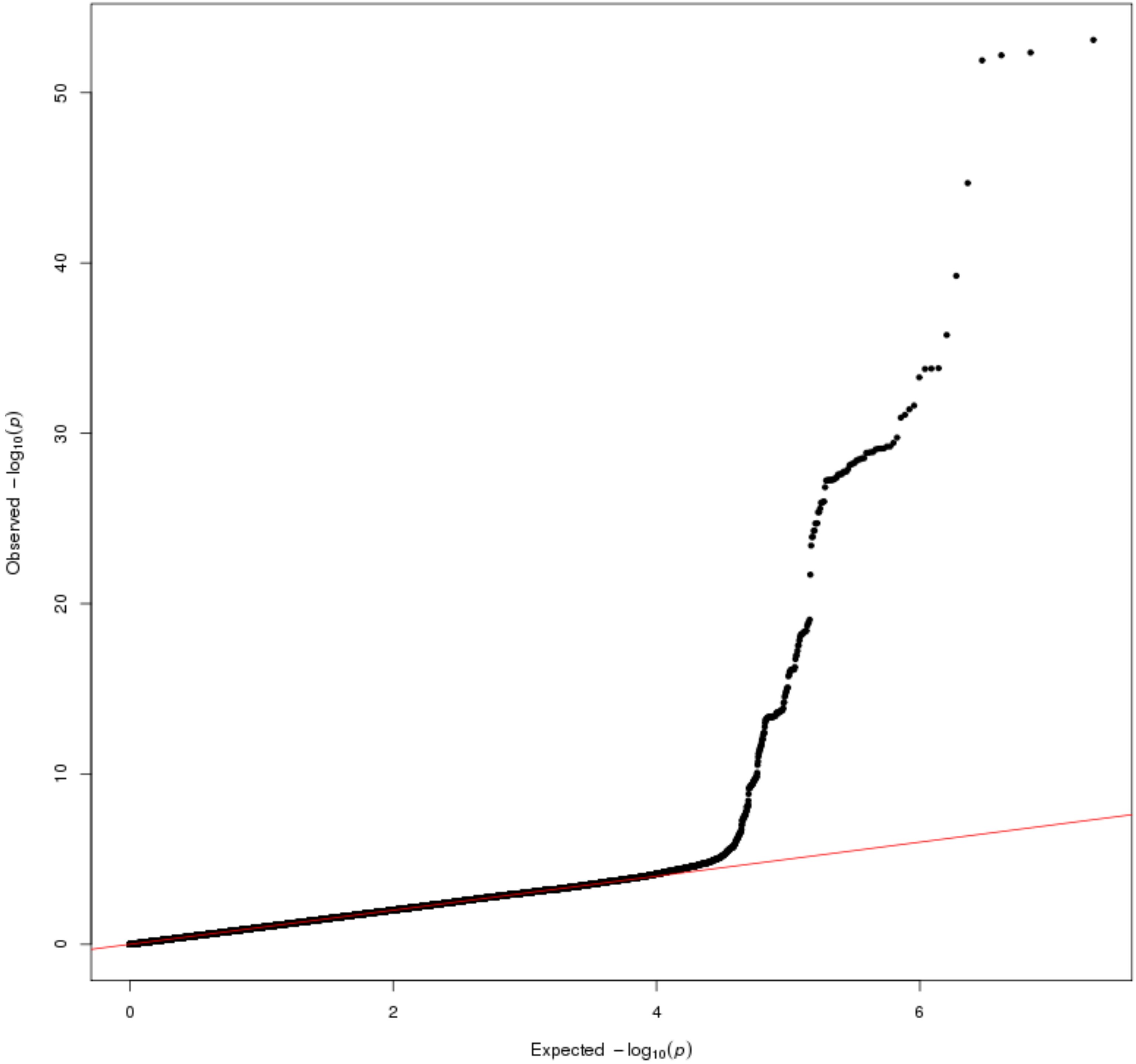

(viii) 3-ureidoisobutyrate

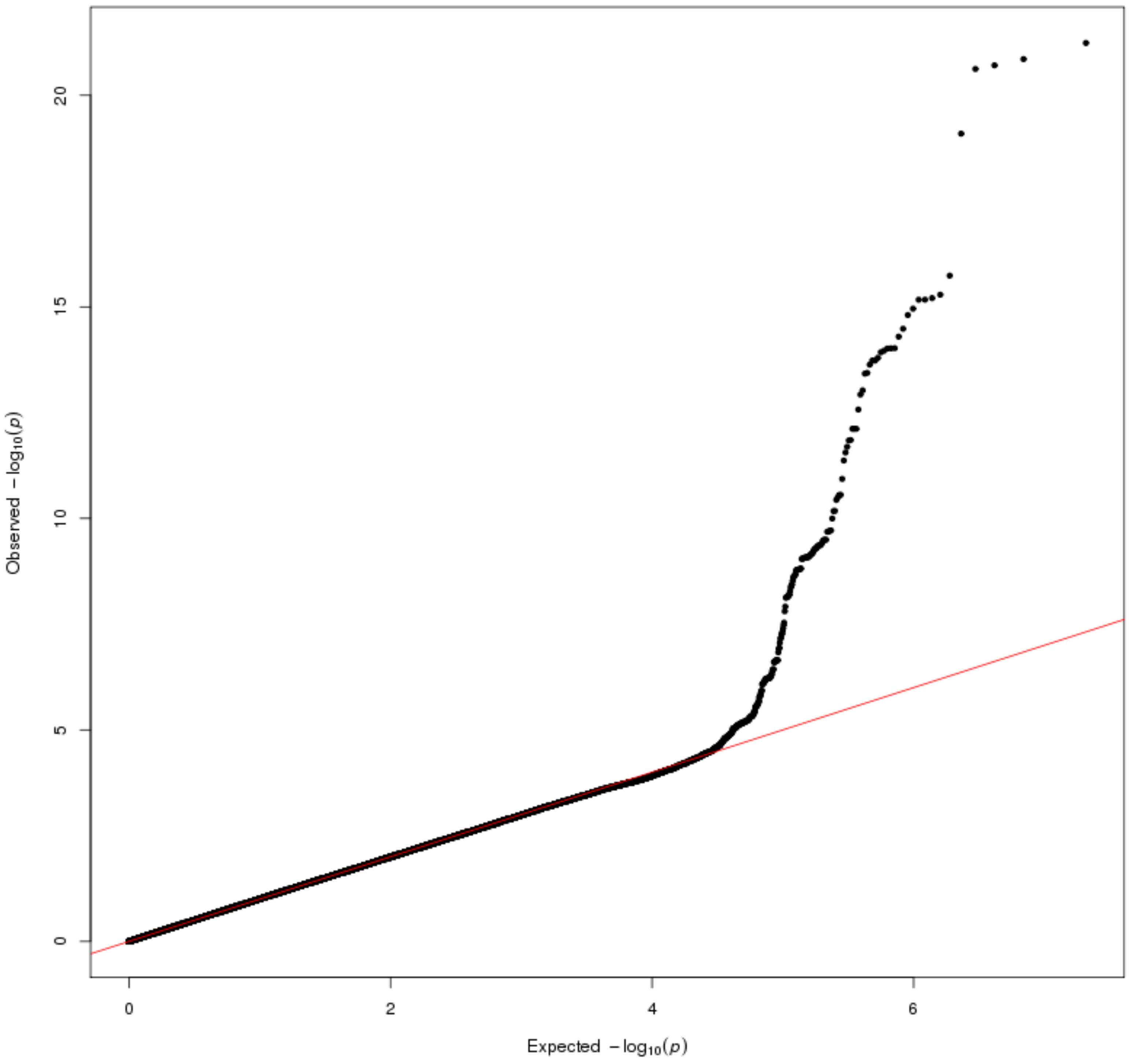

(ix) N-acetylglucosamine/N-acetylgalactosamine

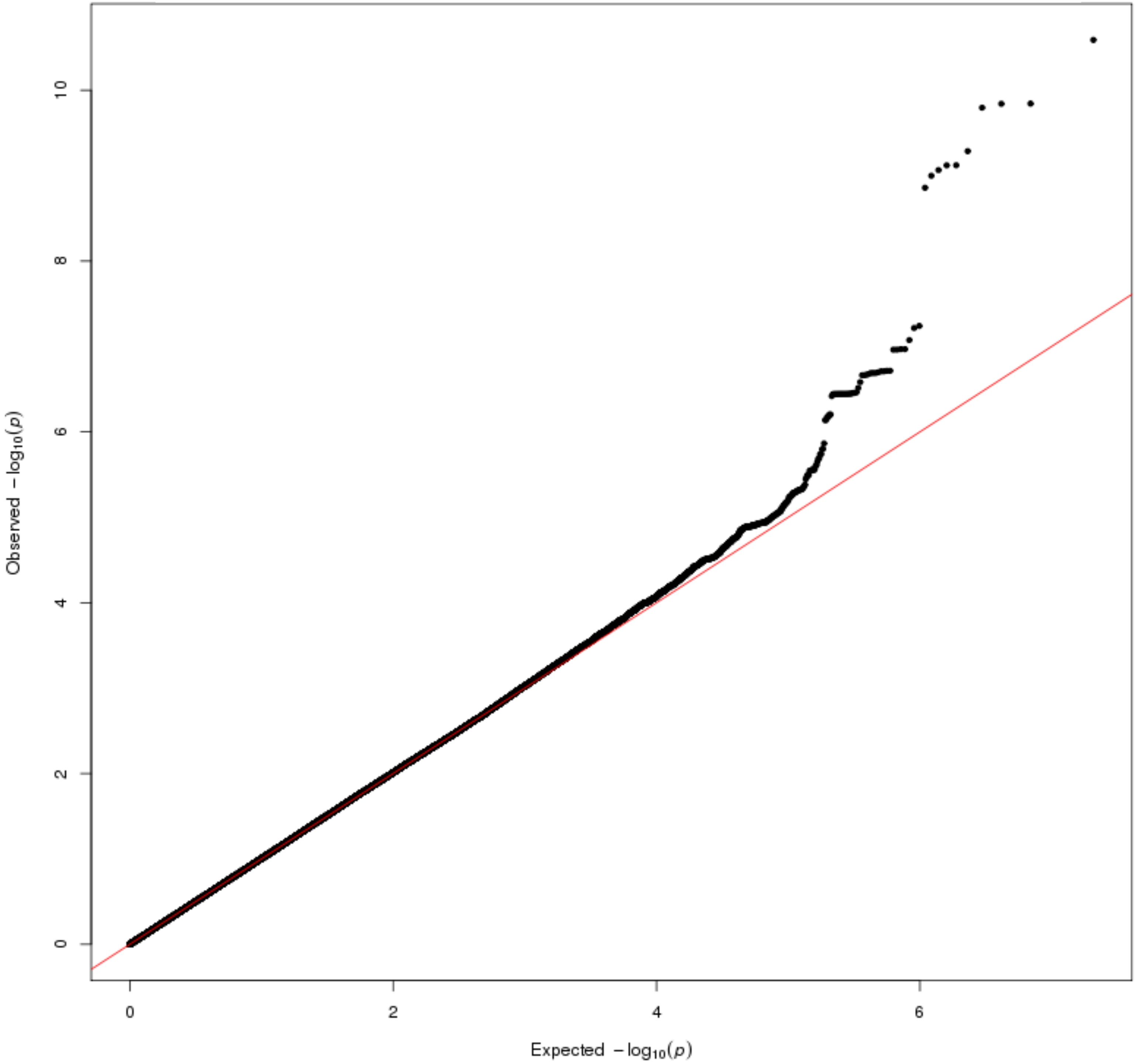

(x) glycosyl-N-stearoyl-sphinganine

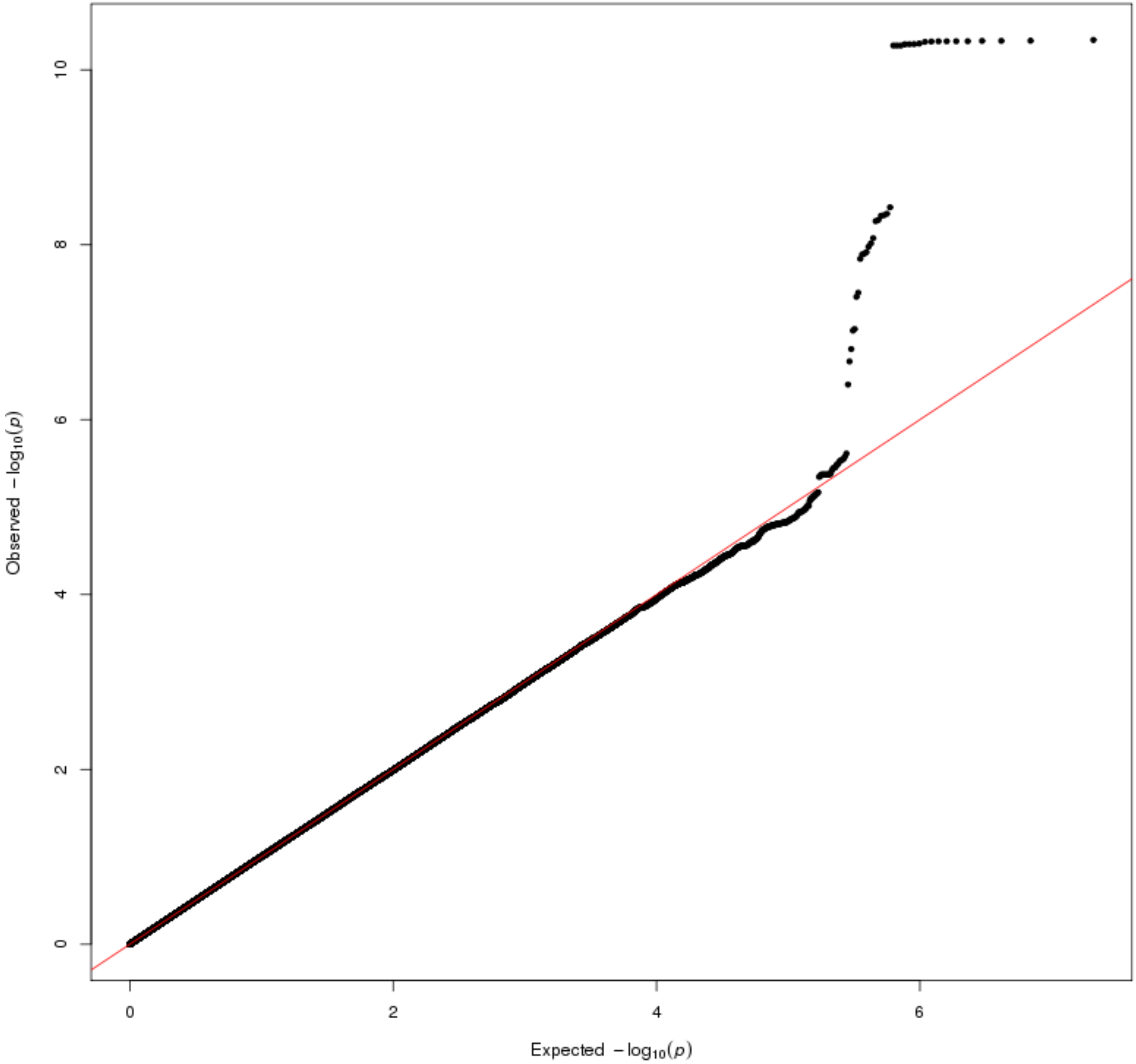

(xi) 1-(1-enyl-palmitoyl)-2-arachidonoyl-GPC

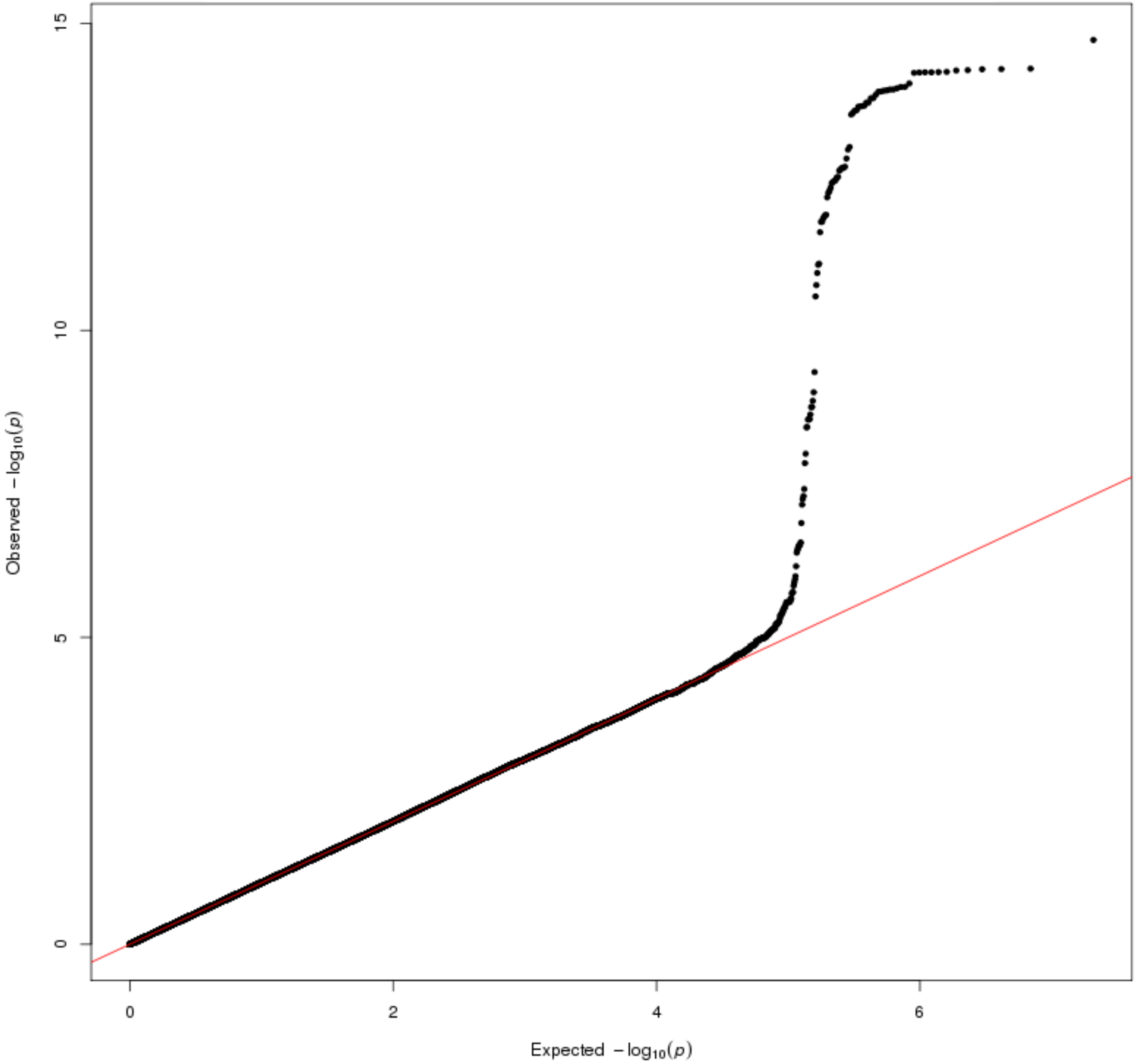

(xii) ethylmalonate

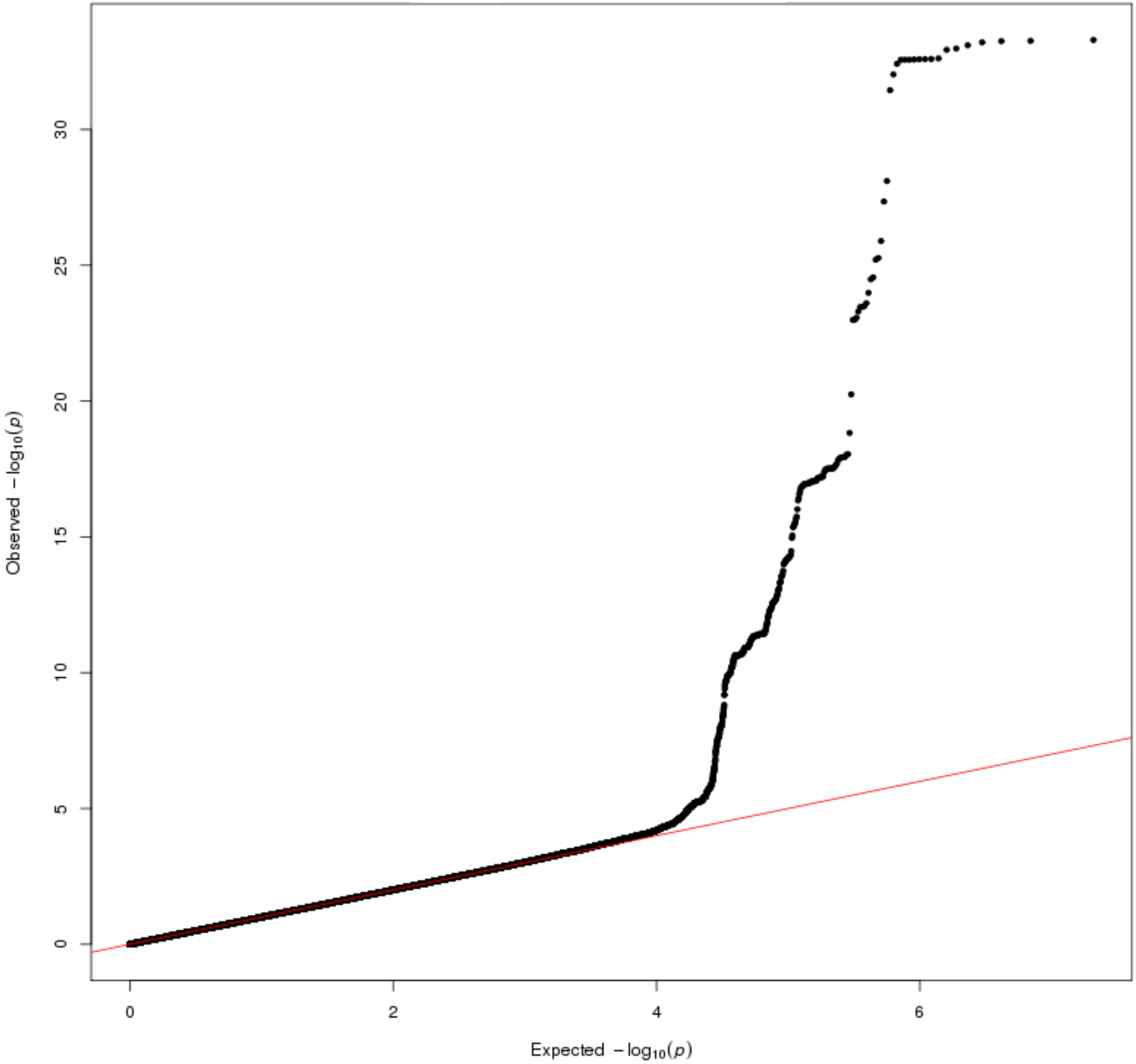

(xiii) gamma-carboxyglutamate

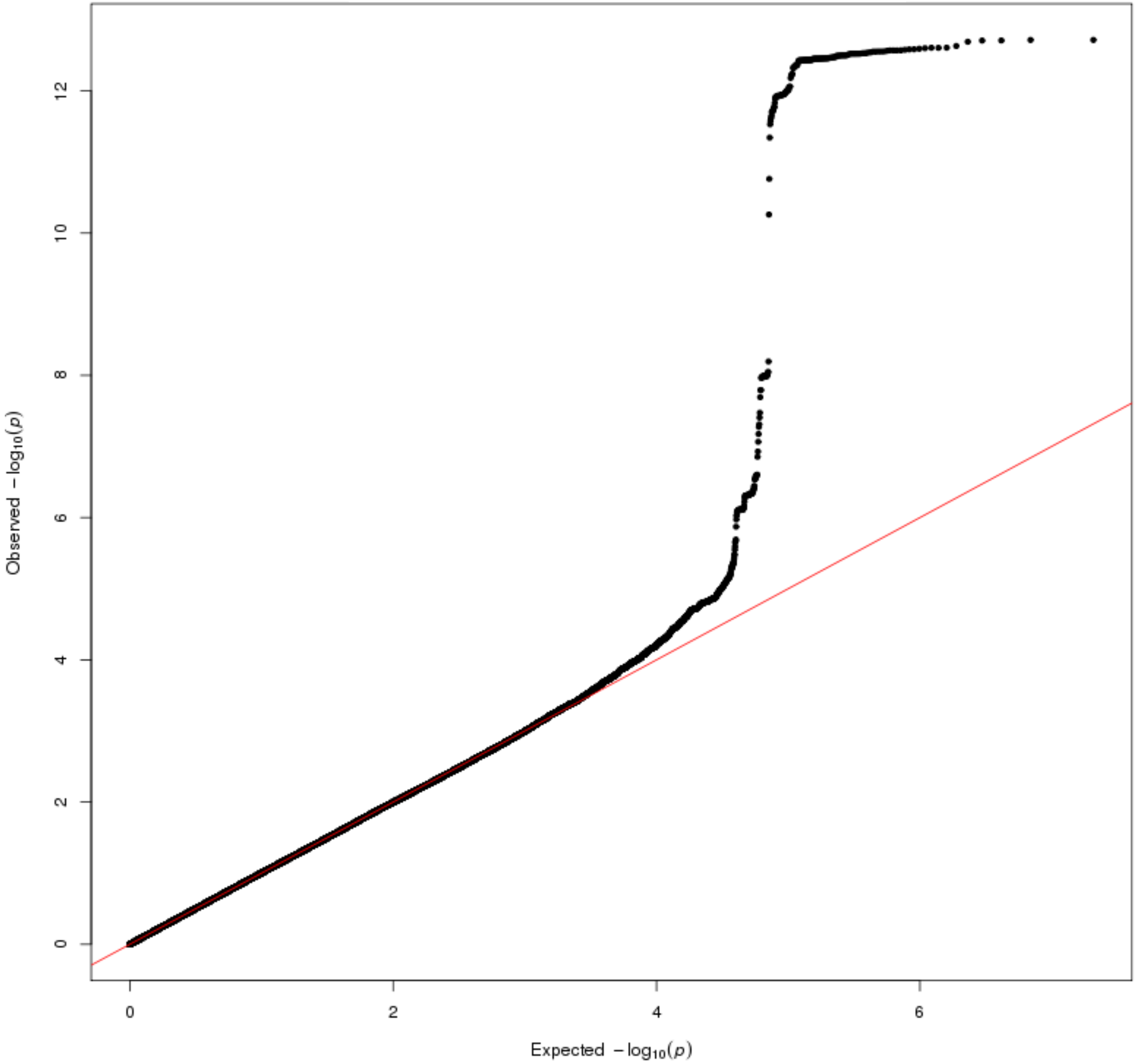

(xiv) ribonate

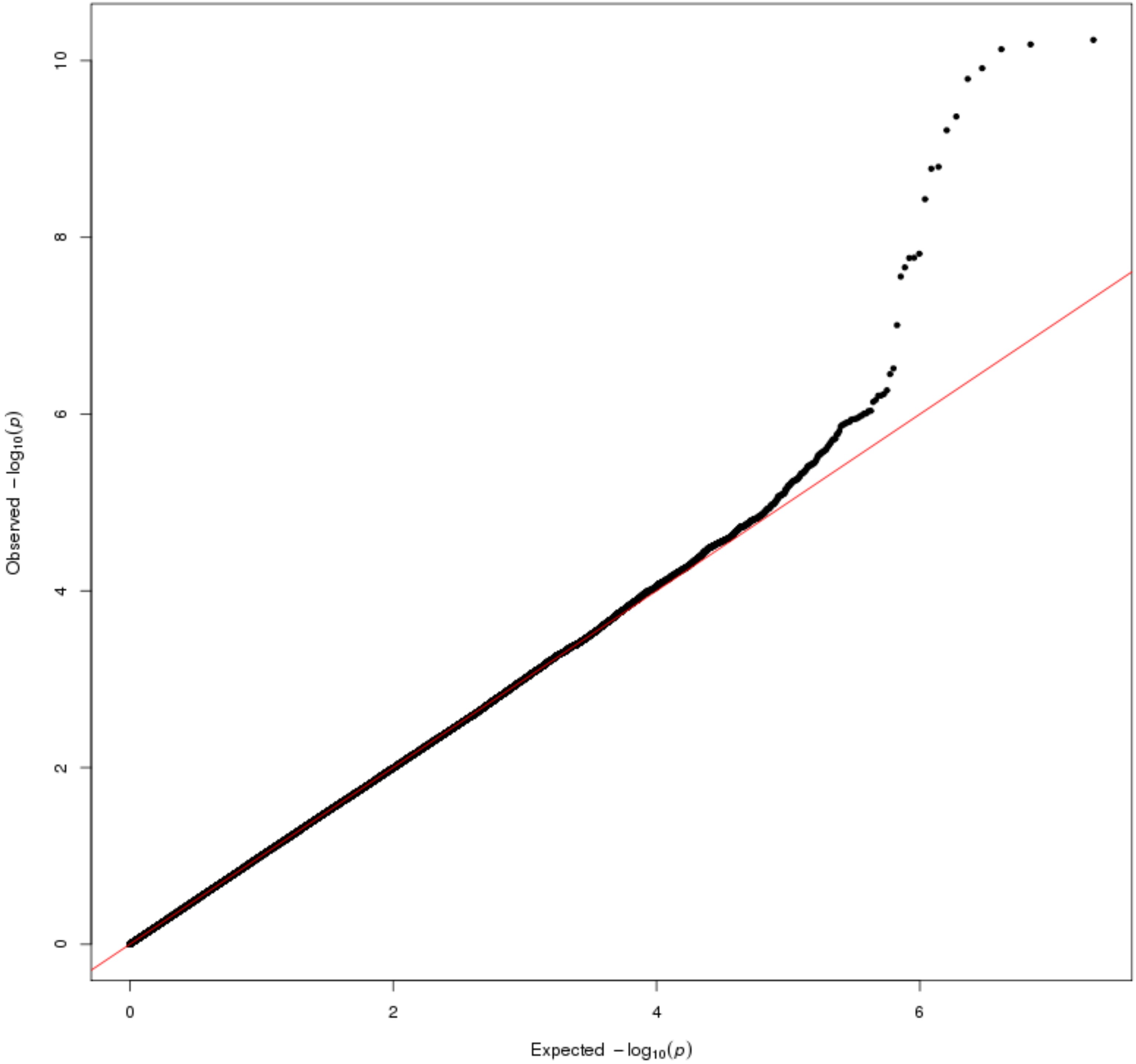

Supplement: FigureS2_ddz308 [file figures2_ddz308.pdf]
