# Supplementary material for: Genome-wide scan identifies novel genetic loci regulating salivary metabolite levels
Source: Hum Mol Genet. 2020 Jan 21;29(5):864–75. doi: 10.1093/hmg/ddz308 (PMC7104674; doi:10.1093/hmg/ddz308)
Supplement: FigureS3_ddz308 [file figures3_ddz308.pdf]

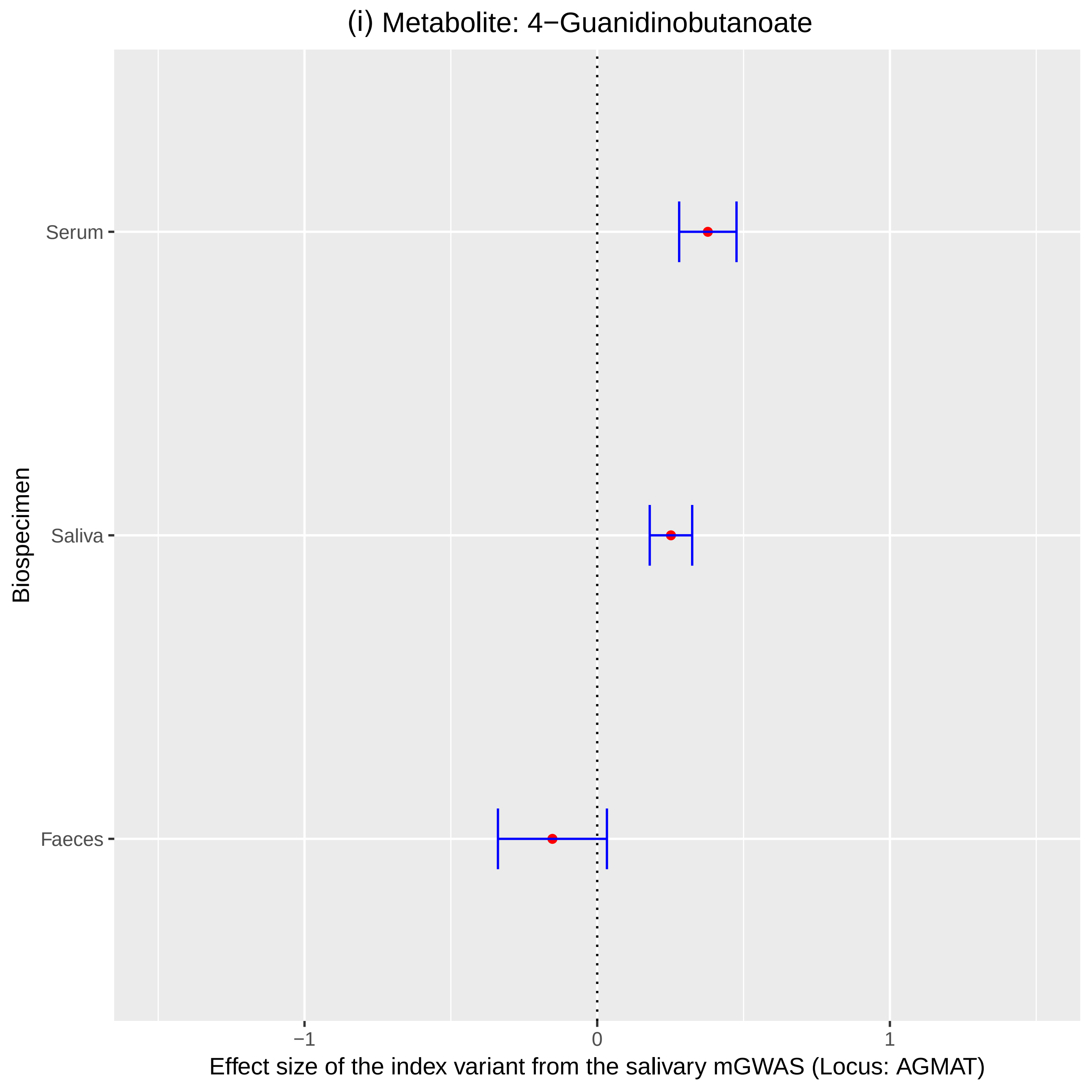

(i) Metabolite: 4−Guanidinobutanoate
Serum
Saliva
Faeces
Biospecimen
−1
0
1
Effect size of the index variant from the salivary mGWAS (Locus: AGMAT)

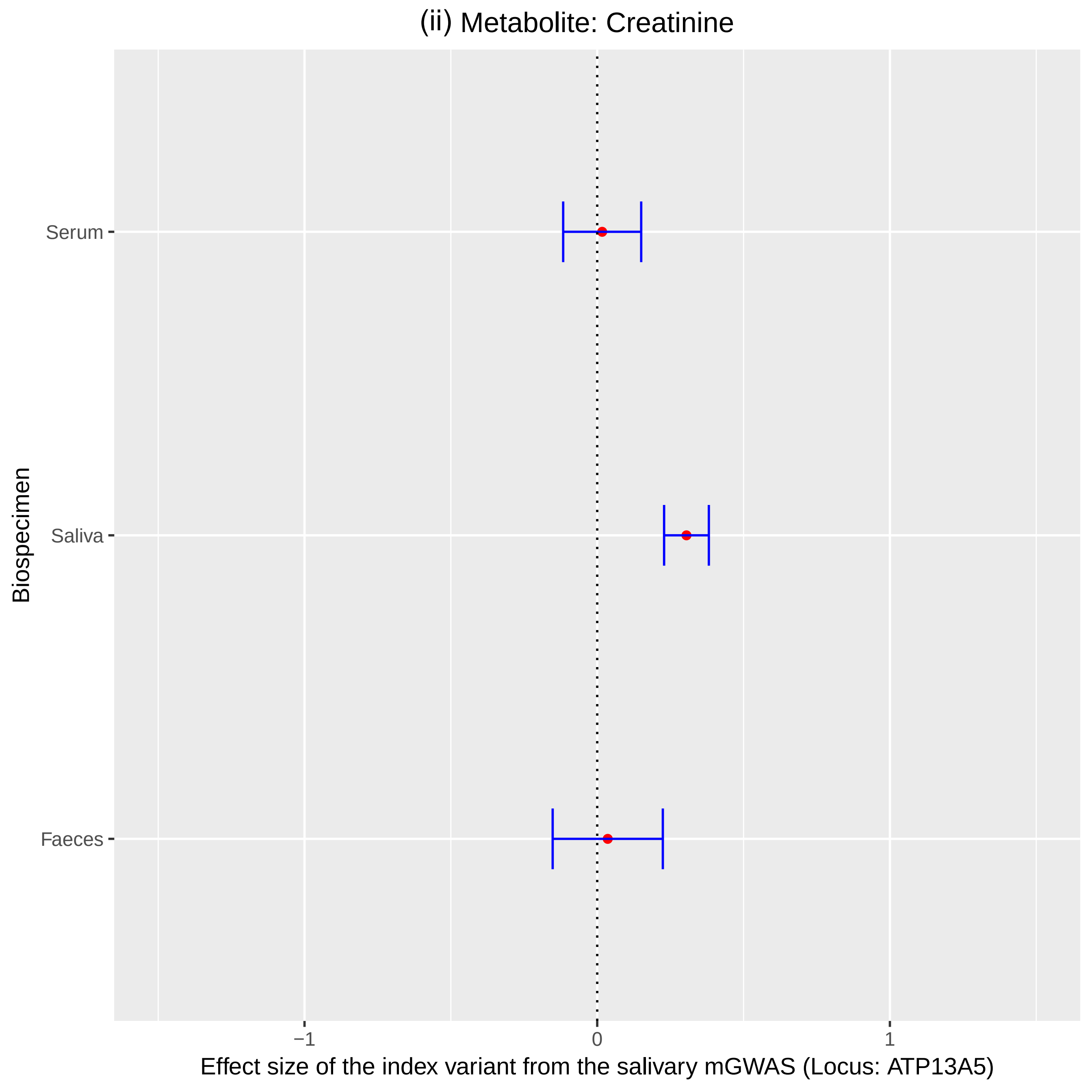

(ii) Metabolite: Creatinine
Serum
Saliva
Faeces
Biospecimen
−1
0
1
Effect size of the index variant from the salivary mGWAS (Locus: ATP13A5)

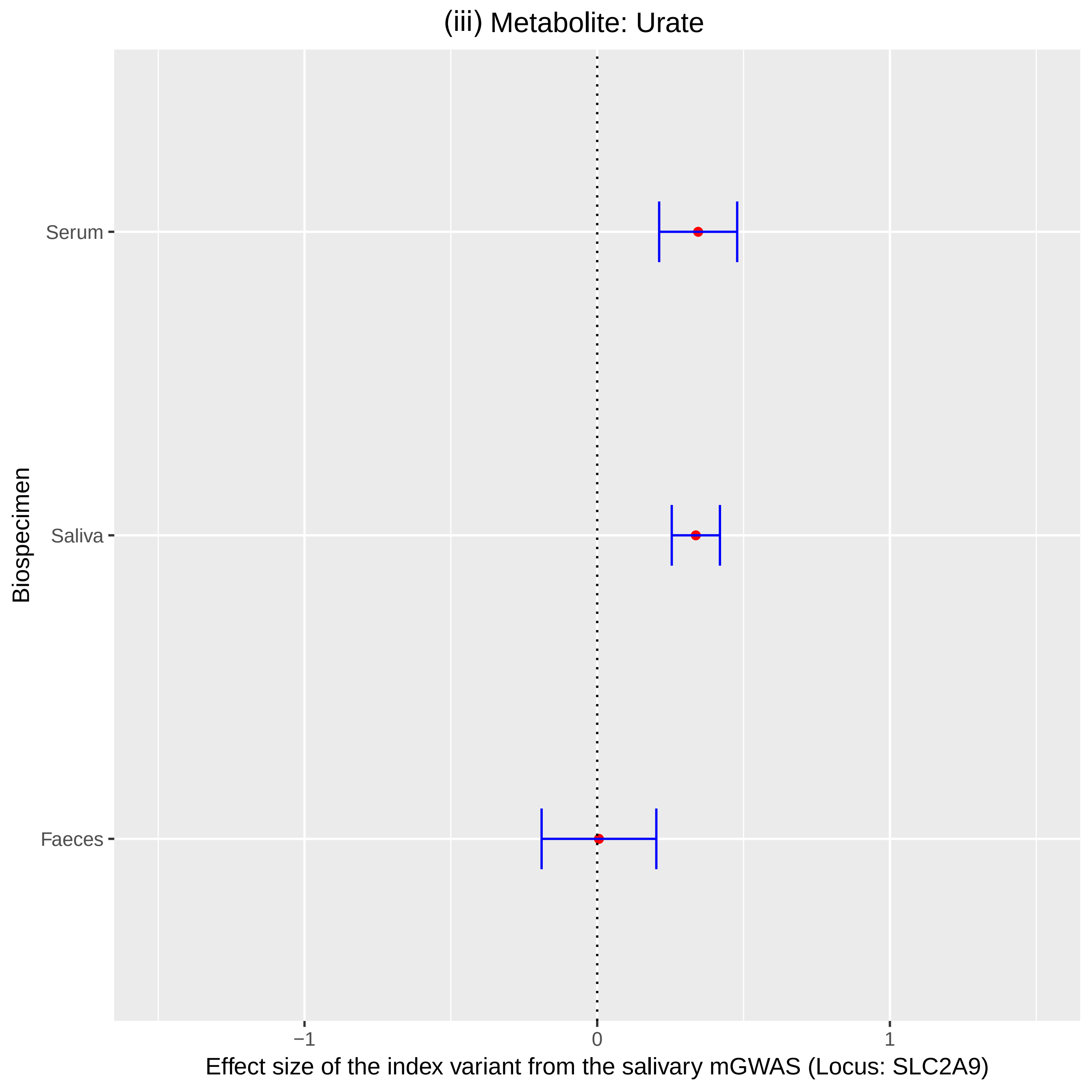

(iii) Metabolite: Urate
Serum
Saliva
Faeces
Biospecimen
−1
0
1
Effect size of the index variant from the salivary mGWAS (Locus: SLC2A9)

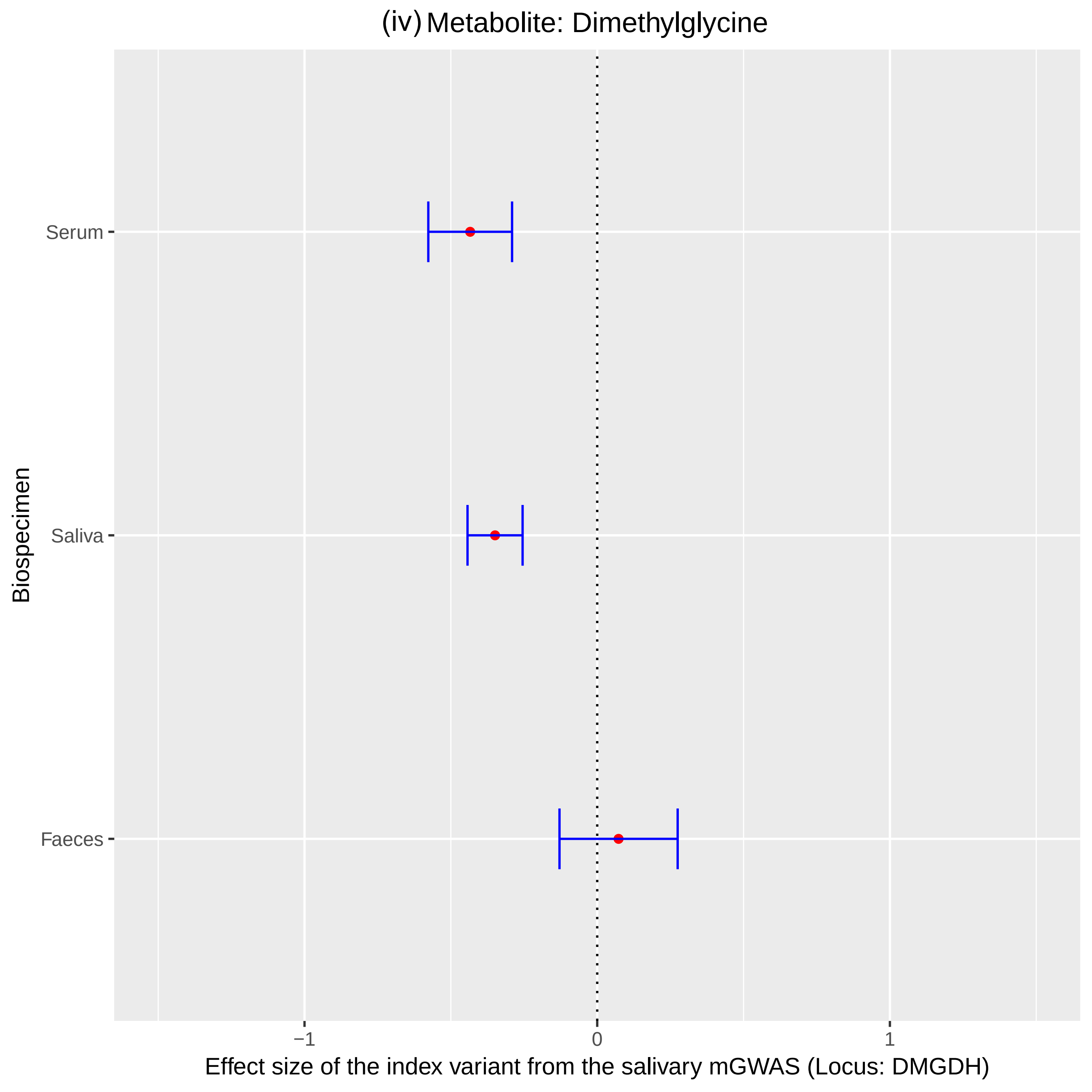

(iv) Metabolite: Dimethylglycine
Serum
Saliva
Faeces
Biospecimen
−1
0
1
Effect size of the index variant from the salivary mGWAS (Locus: DMGDH)

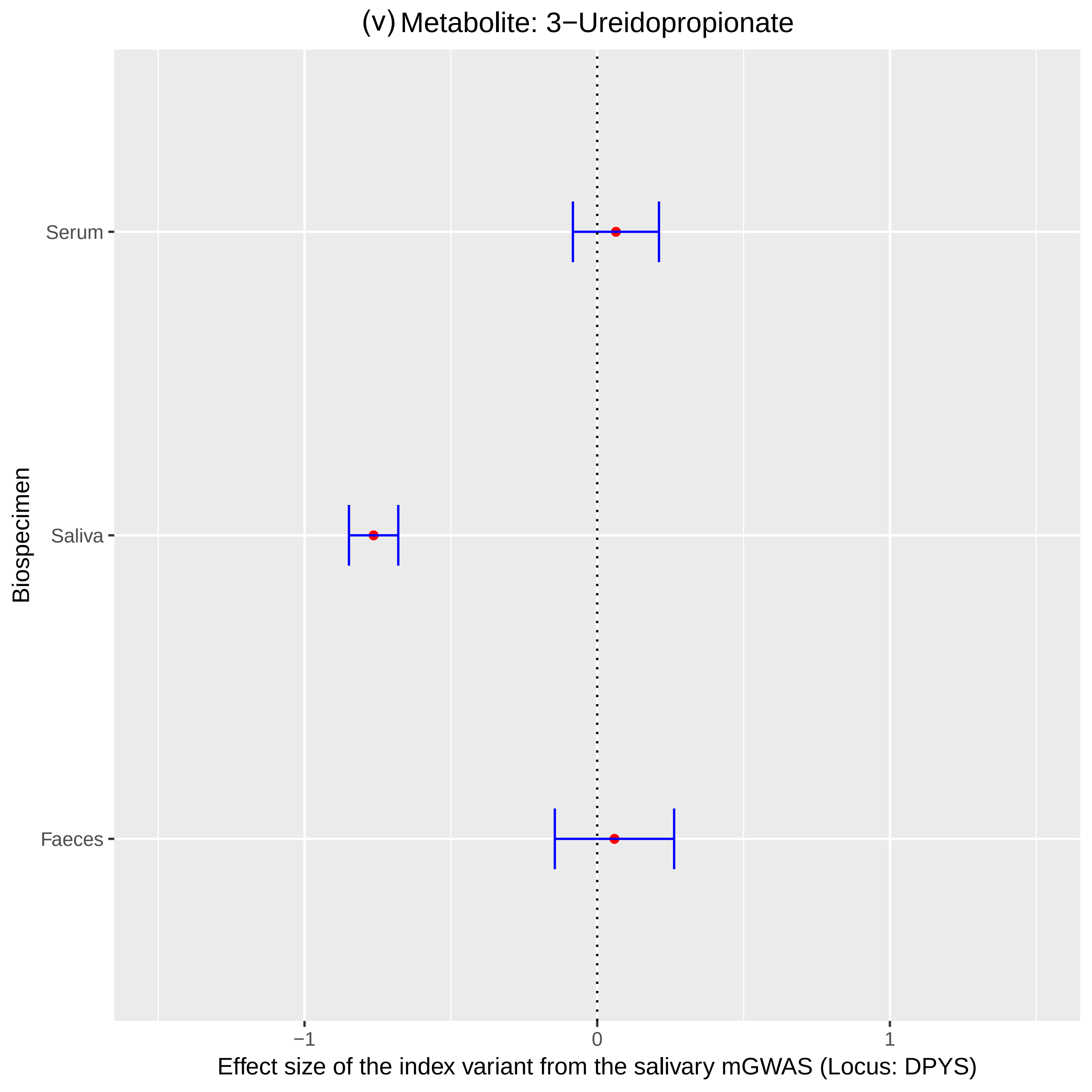

(v) Metabolite: 3−Ureidopropionate
Serum
Saliva
Faeces
Biospecimen
−1
0
1
Effect size of the index variant from the salivary mGWAS (Locus: DPYS)

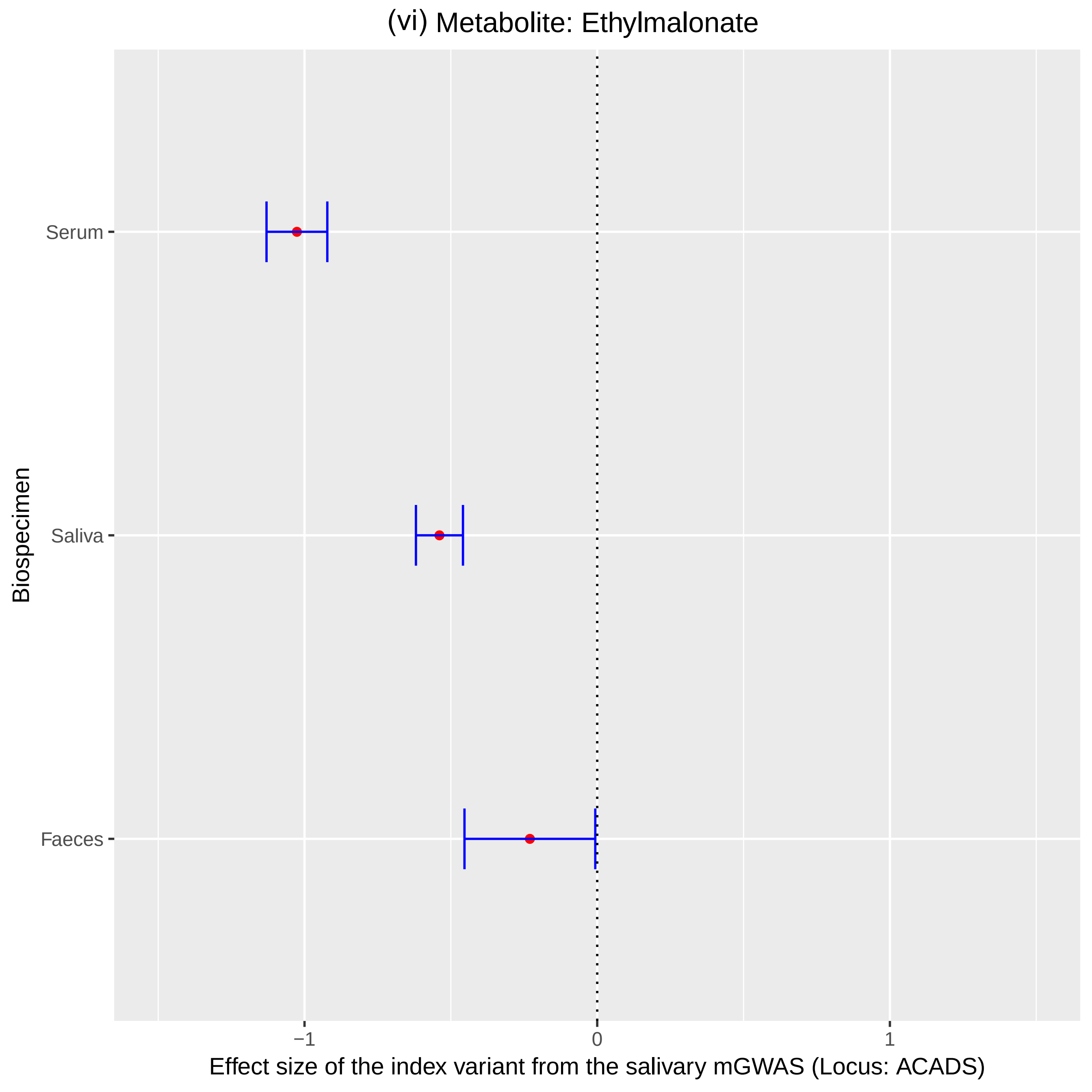

(vi) Metabolite: Ethylmalonate
Serum
Saliva
Faeces
Biospecimen
−1
0
1
Effect size of the index variant from the salivary mGWAS (Locus: ACADS)

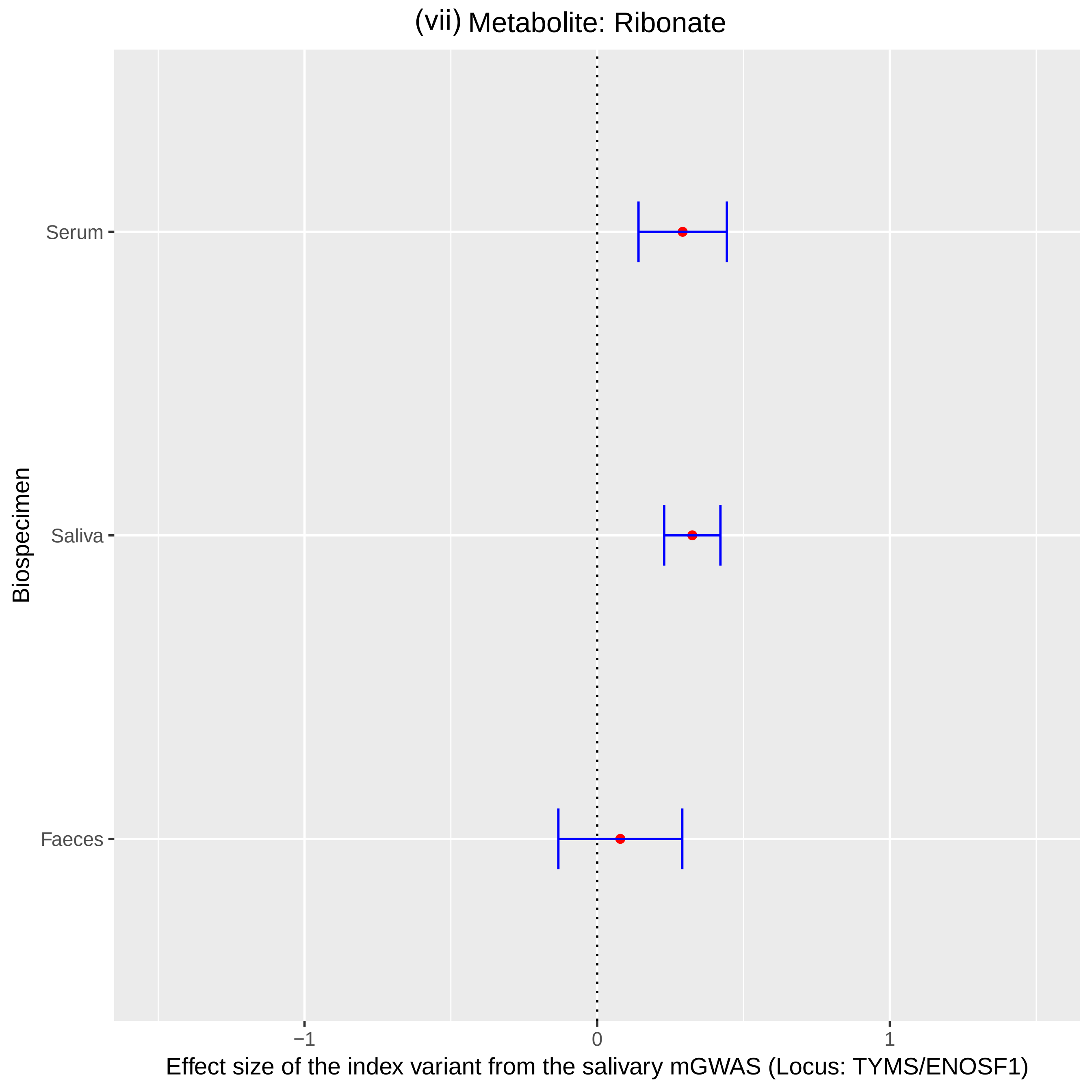
(vii) Metabolite: Ribonate
Serum
Saliva
Faeces
Biospecimen
−1
0
1
Effect size of the index variant from the salivary mGWAS (Locus: TYMS/ENOSF1)
