# Supplementary material for: Genome-wide scan identifies novel genetic loci regulating salivary metabolite levels
Source: Hum Mol Genet. 2020 Jan 21;29(5):864–75. doi: 10.1093/hmg/ddz308 (PMC7104674; doi:10.1093/hmg/ddz308)

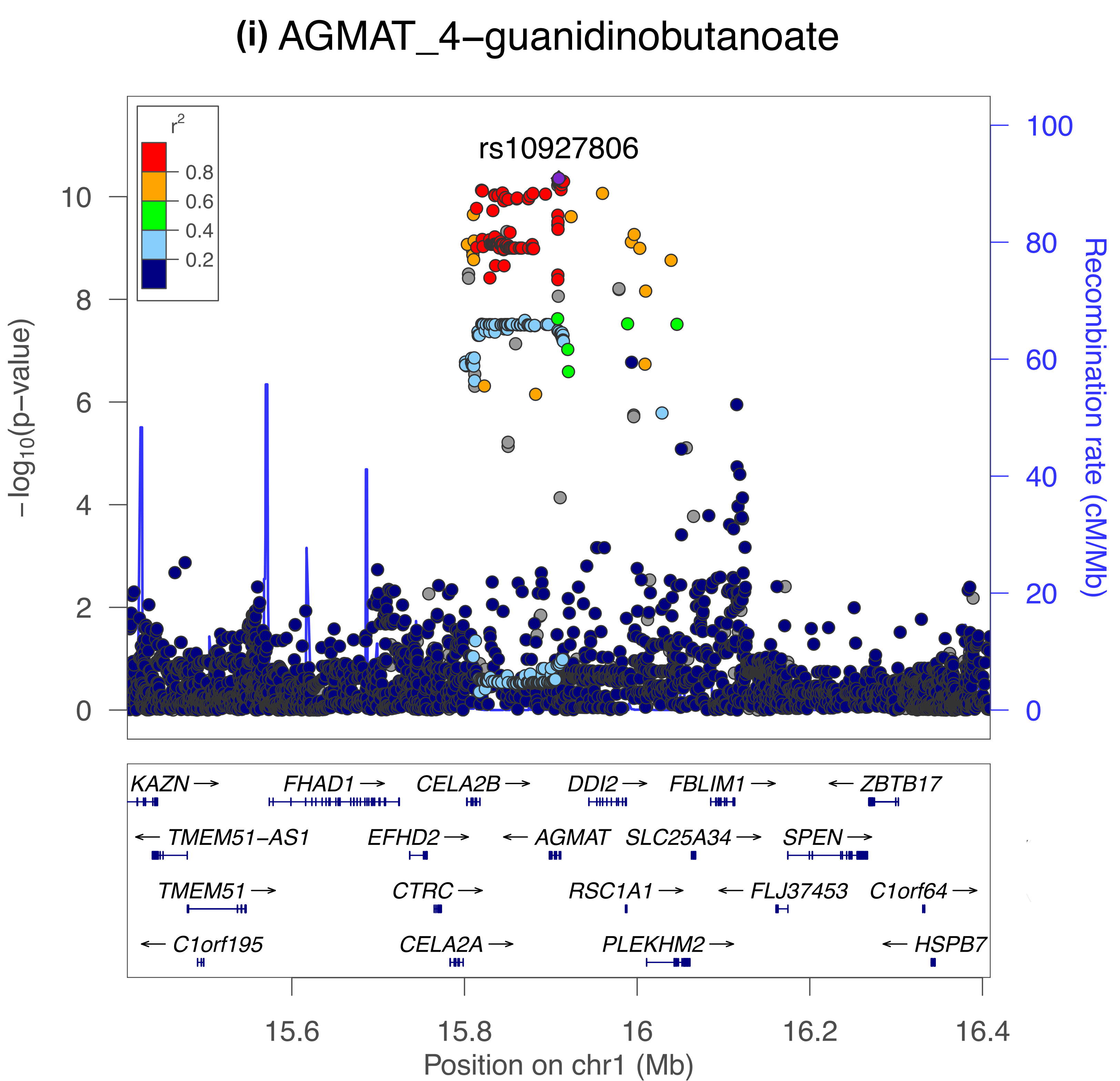

(i) AGMAT_4−guanidinobutanoate
rs10927806
r²
0.8
0.6
0.4
0.2
−log10(p−value)
Recombination rate (cM/Mb)
0
2
4
6
8
10
0
20
40
60
80
100
KAZN →
FHAD1 →
CELA2B →
DDI2 →
FBLIM1 →
← ZBTB17
← TMEM51−AS1
EFHD2 →
← AGMAT
SLC25A34 →
SPEN →
TMEM51 →
CTRC →
RSC1A1 →
← FLJ37453
C1orf64 →
← C1orf195
CELA2A →
PLEKHM2 →
← HSPB7
15.6
15.8
16
16.2
16.4
Position on chr1 (Mb)

(ii) AGMAT_beta−guanidinopropanoate

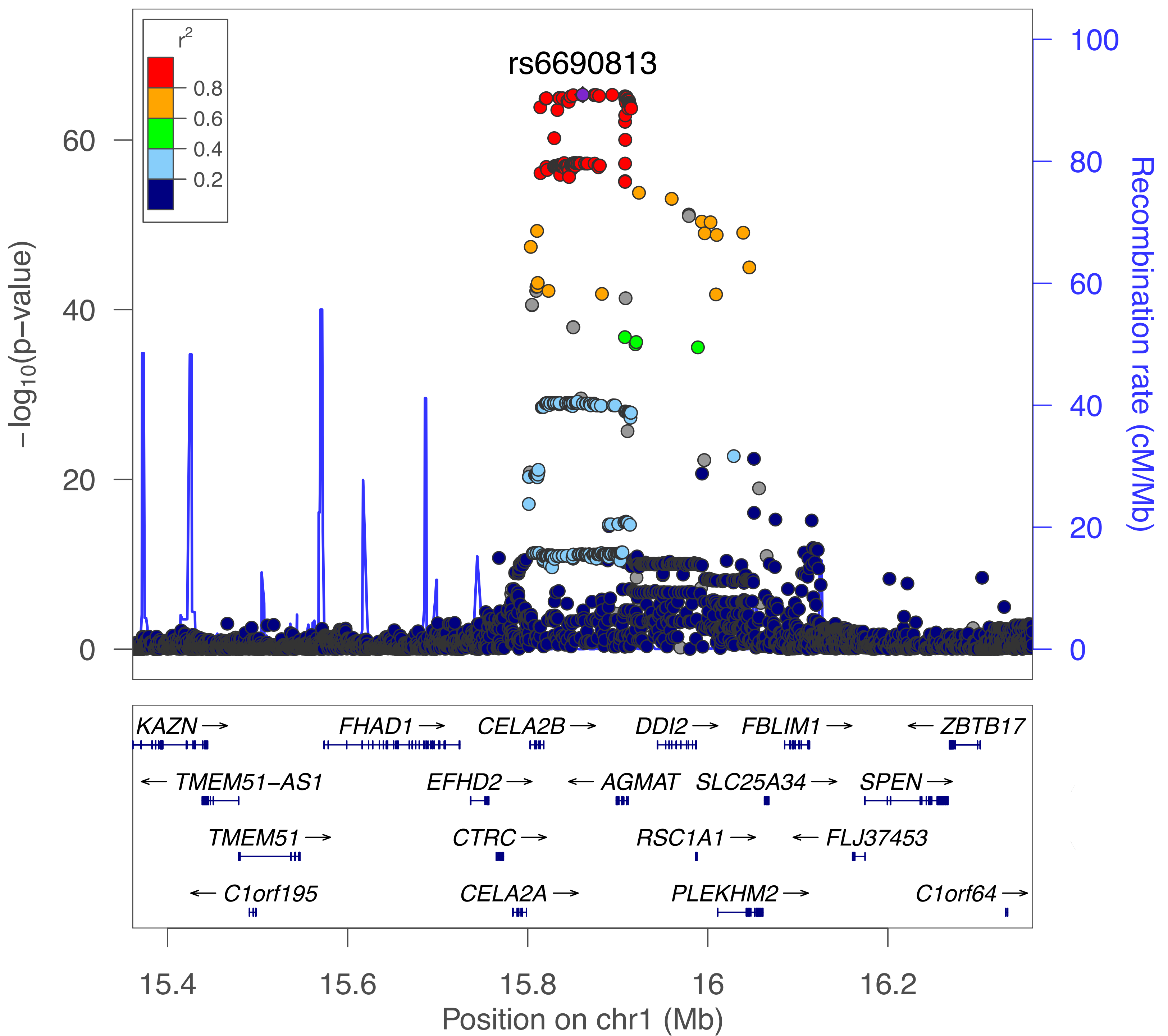

(iii) ATP13A5_creatinine

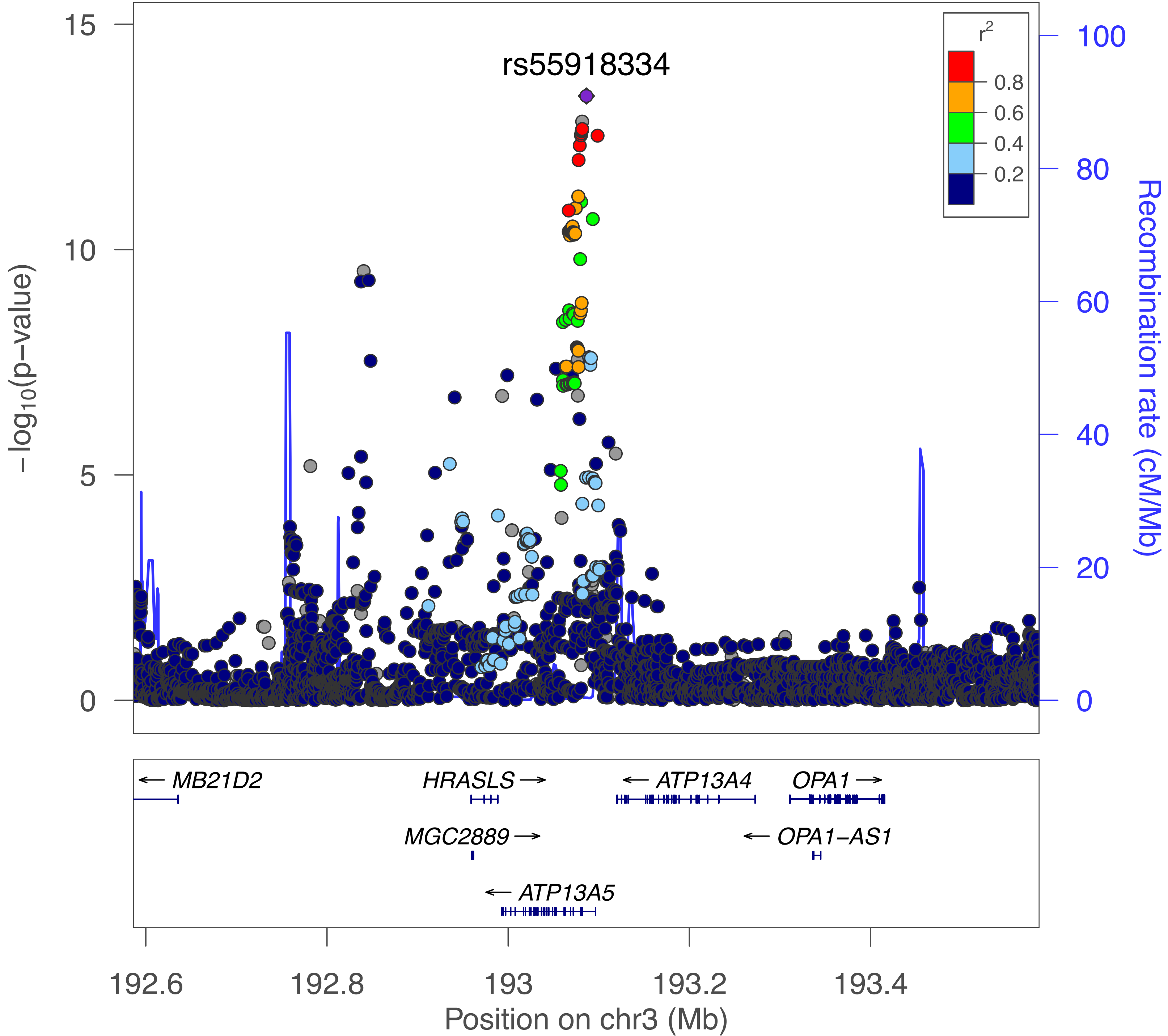

(iv) SLC2A9_urate

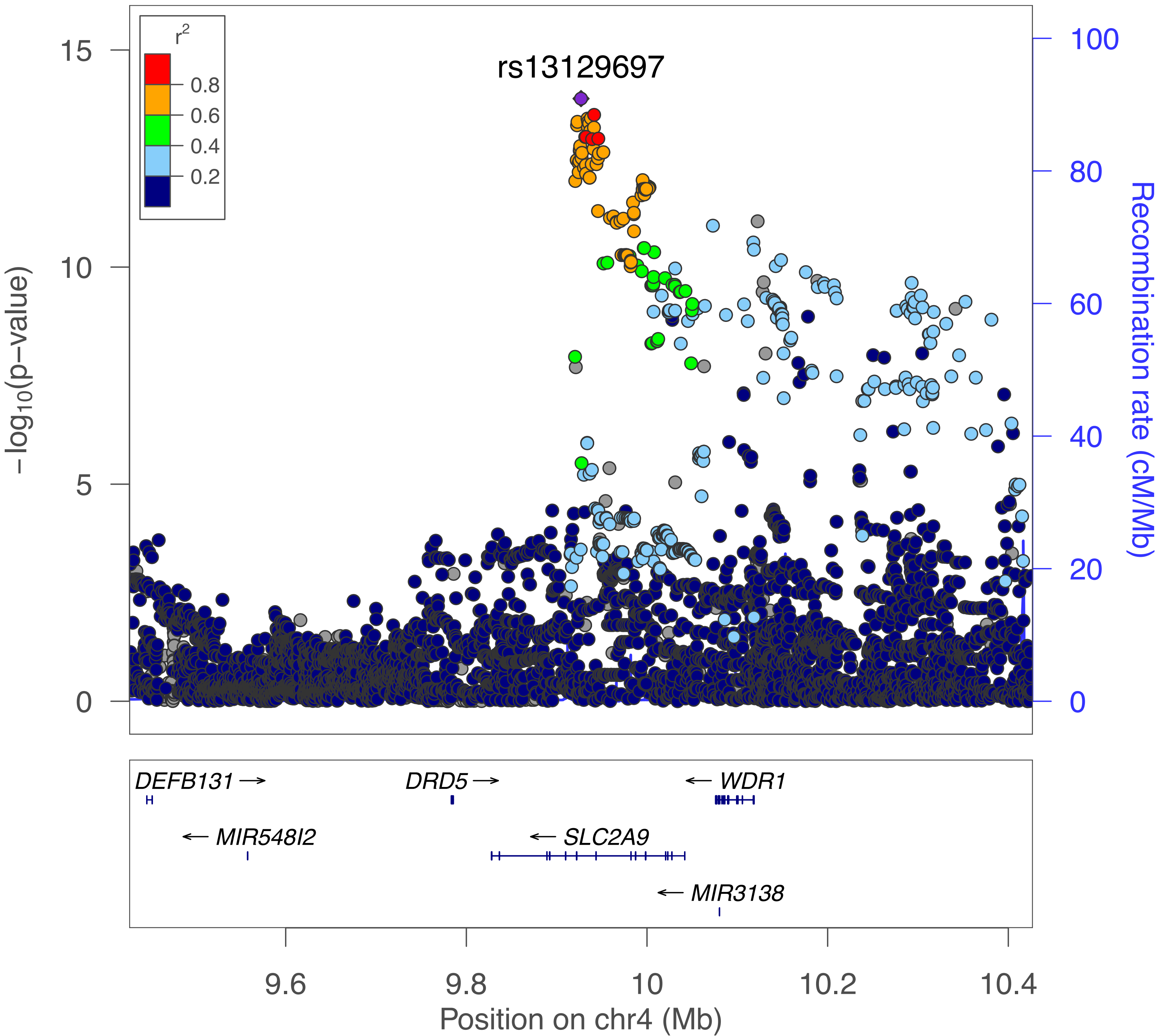

(v) SLC2A9_allantoin

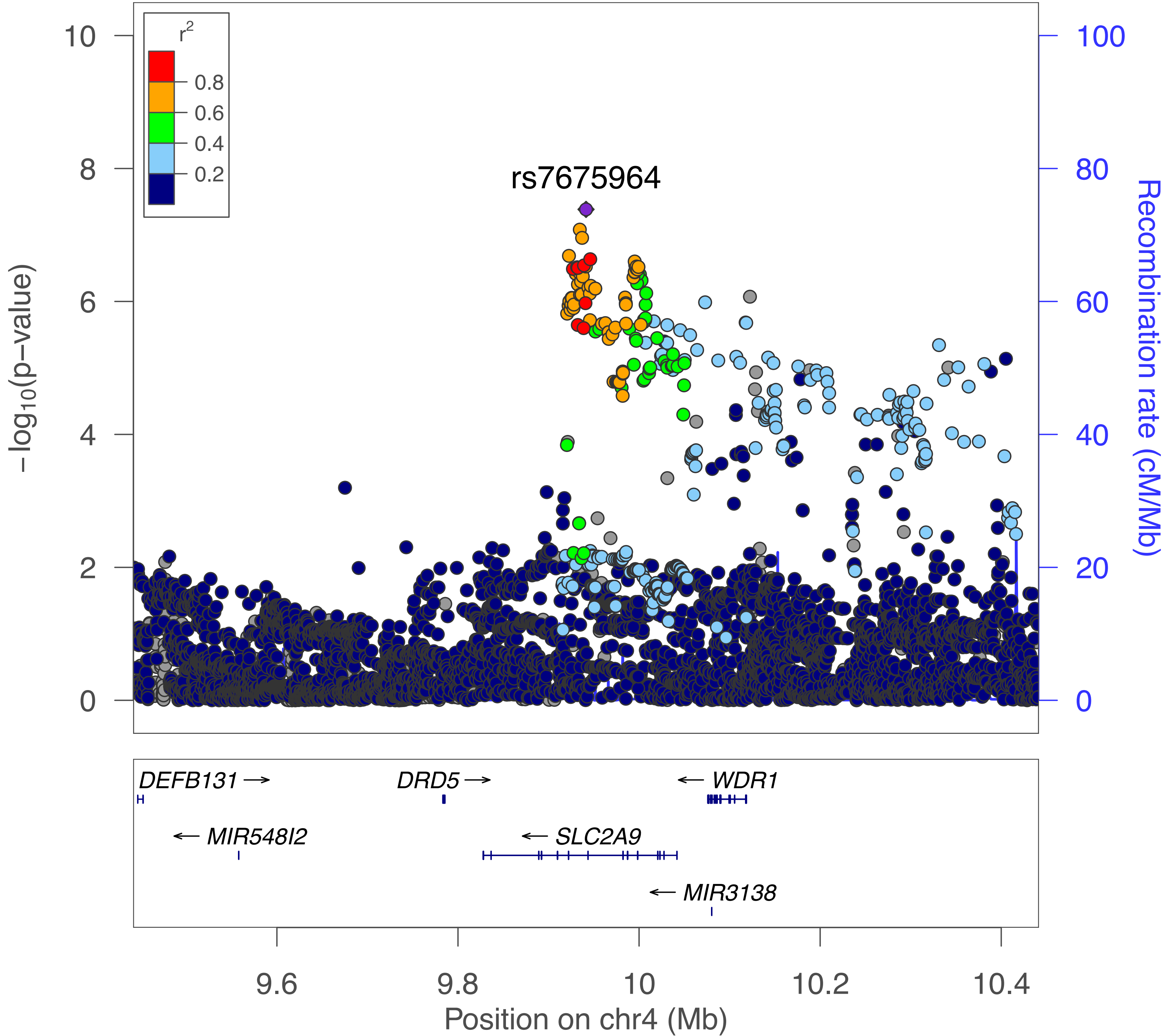

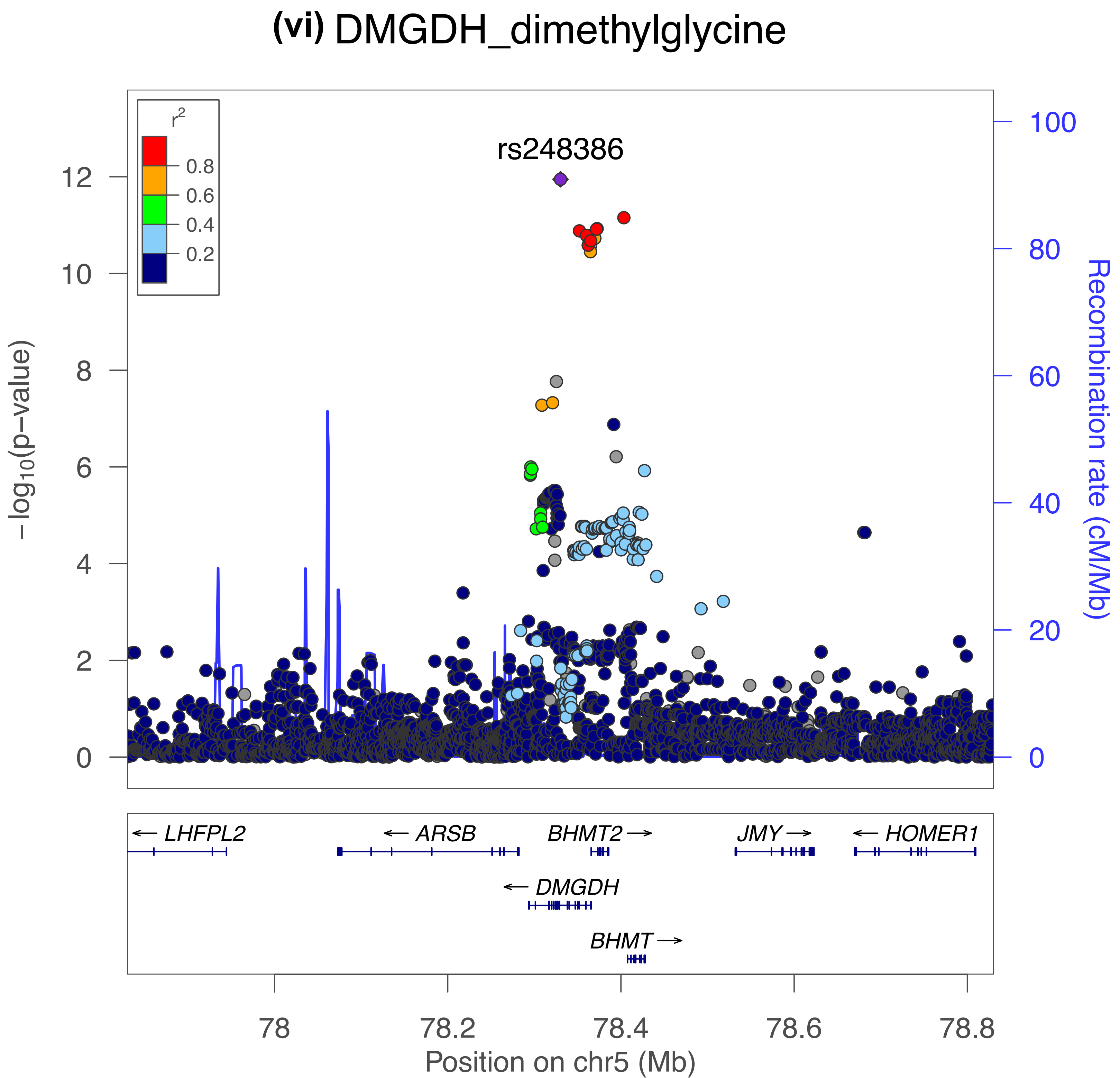
(vi) DMGDH_dimethylglycine
r2
0.8
0.6
0.4
0.2
rs248386
12
10
8
6
4
2
0
−log10(p−value)
100
80
60
40
20
0
Recombination rate (cM/Mb)
← LHFPL2
← ARSB
BHMT2 →
JMY →
← HOMER1
← DMGDH
BHMT →
78
78.2
78.4
78.6
78.8
Position on chr5 (Mb)

(vii) DPYS_3−ureidopropionate

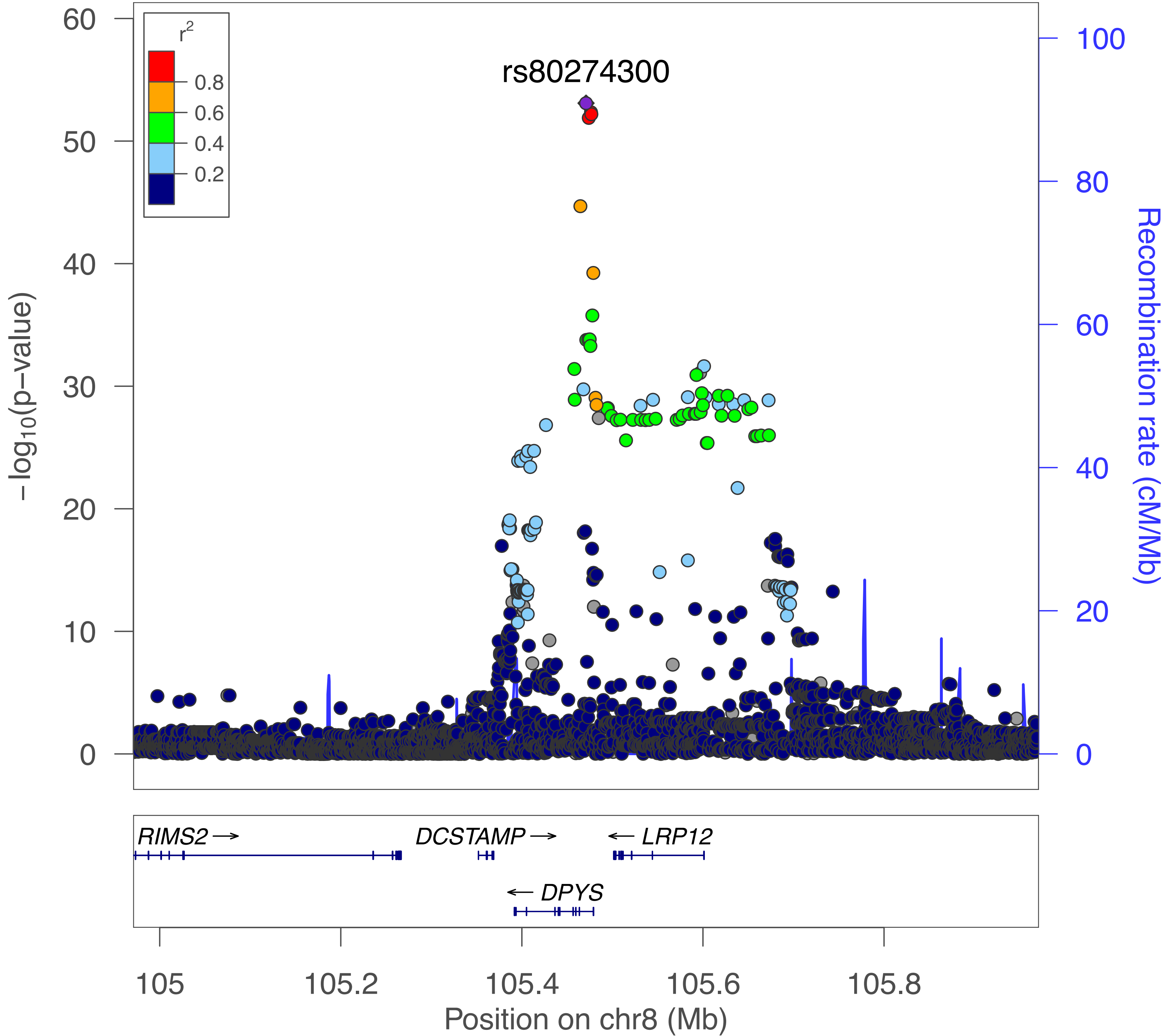

(viii) DPYS_3−ureidoisobutyrate

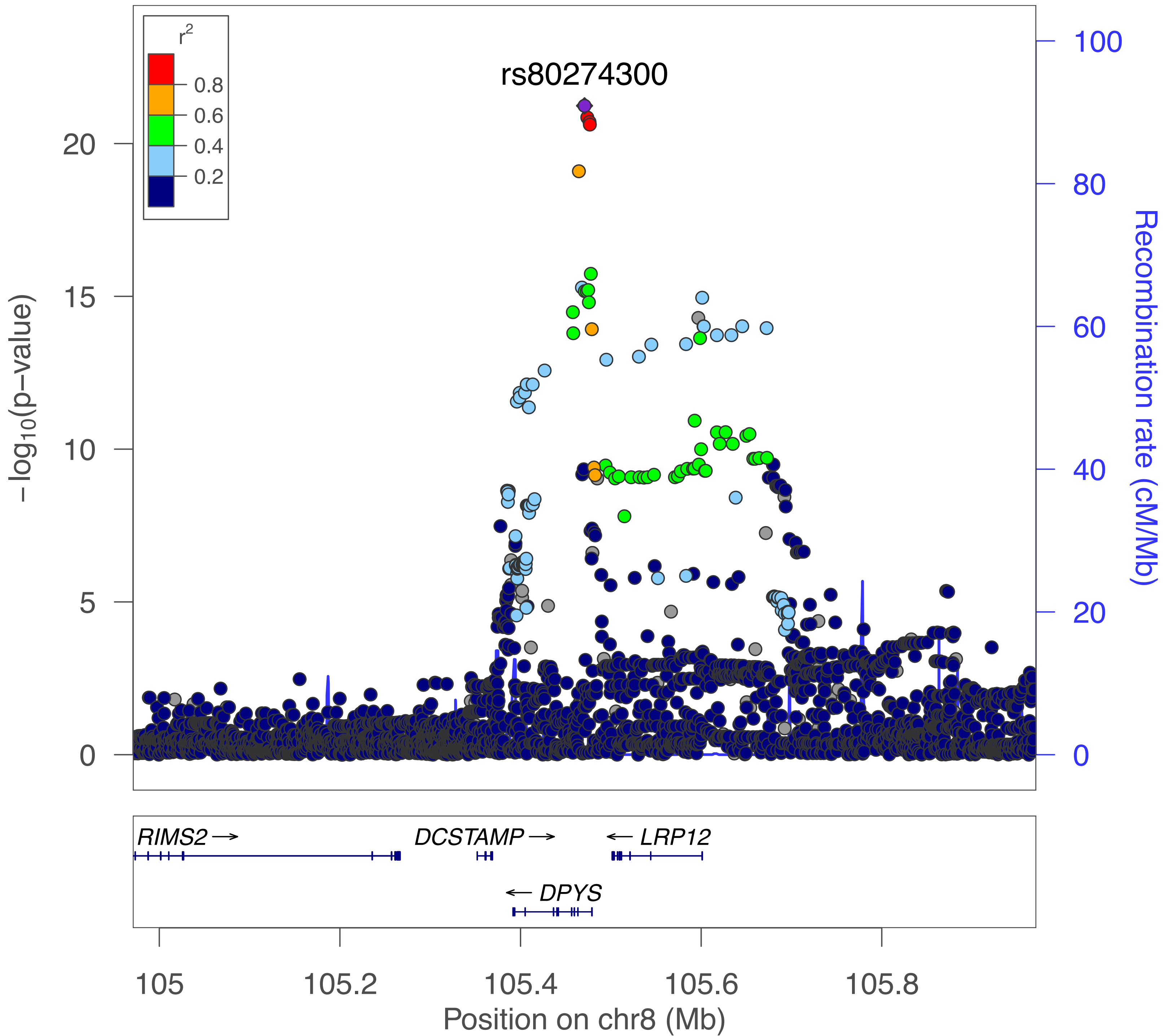

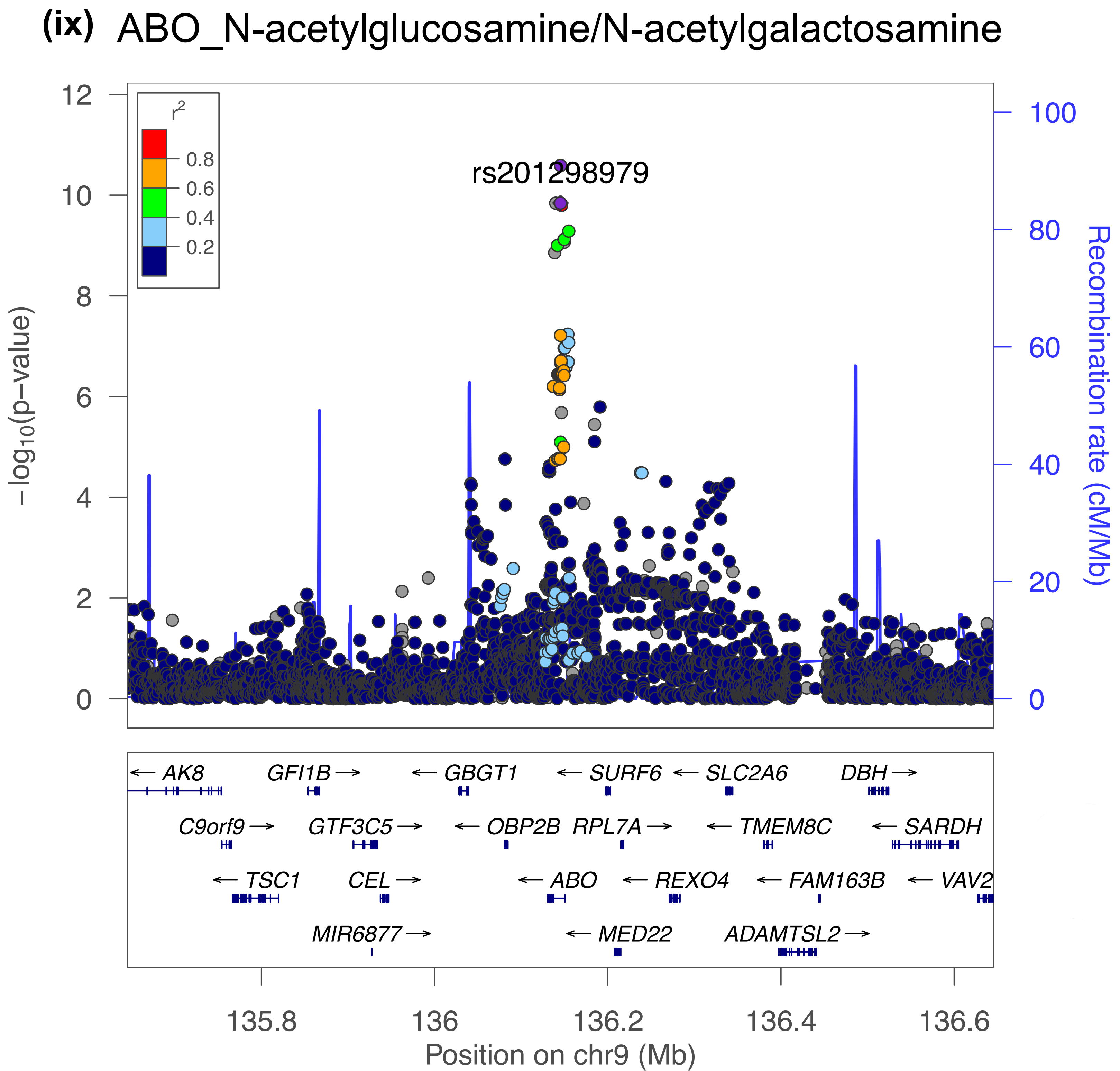
(ix) ABO_N-acetylglucosamine/N-acetylgalactosamine
r²
0.8
0.6
0.4
0.2
rs201298979
12
10
8
6
4
2
0
−log10(p−value)
100
80
60
40
20
0
Recombination rate (cM/Mb)
← AK8
GFI1B →
← GBGT1
← SURF6
← SLC2A6
DBH →
C9orf9 →
GTF3C5 →
← OBP2B
RPL7A →
← TMEM8C
← SARDH
← TSC1
CEL →
← ABO
← REXO4
← FAM163B
← VAV2
MIR6877 →
← MED22
ADAMTSL2 →
135.8
136
136.2
136.4
136.6
Position on chr9 (Mb)

(x) UGCG_glycosyl−N−stearoyl−sphinganine

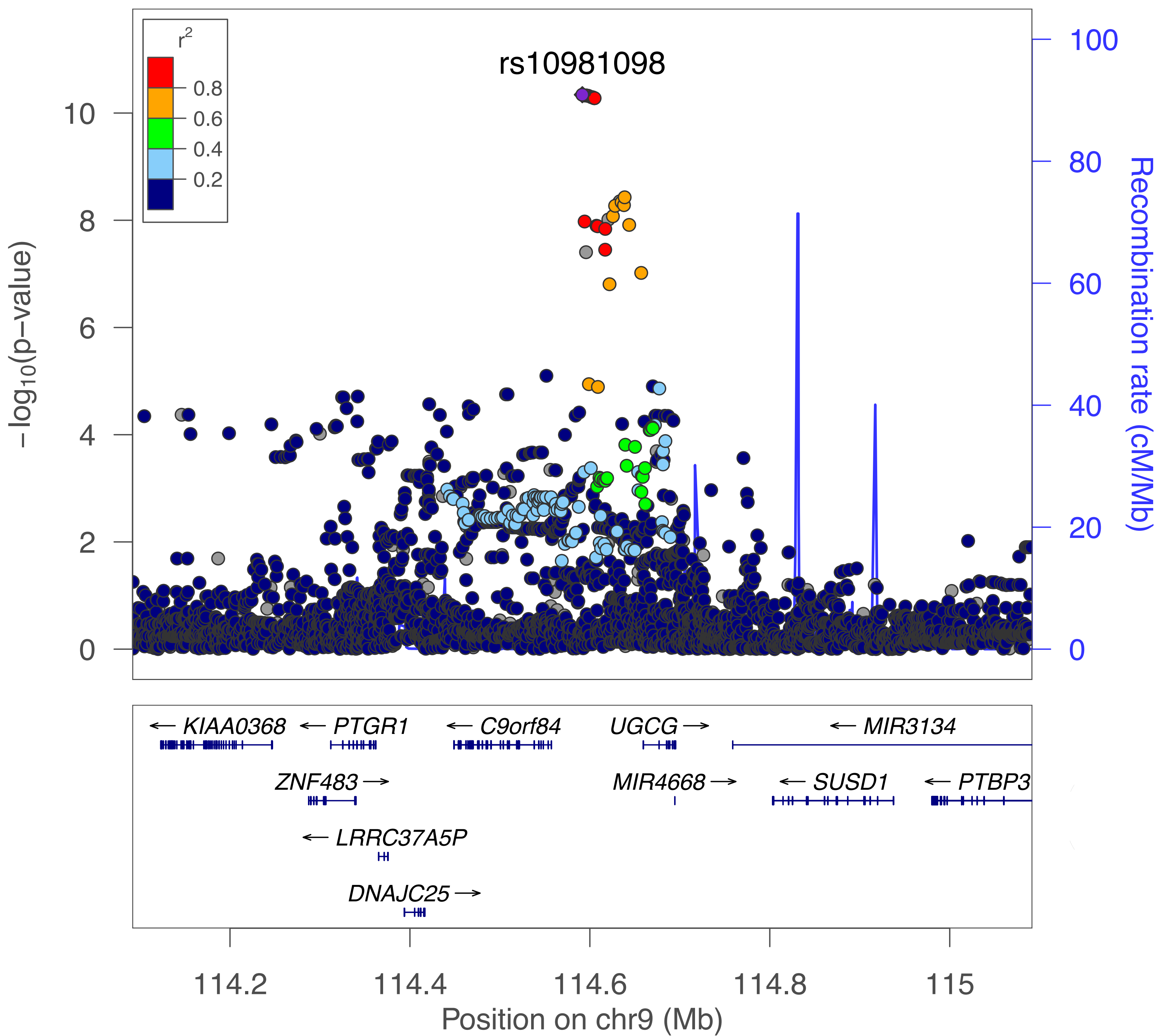

(xi) FADS2_1-(1-enyl-palmitoyl)-2-arachidonoyl-GPC

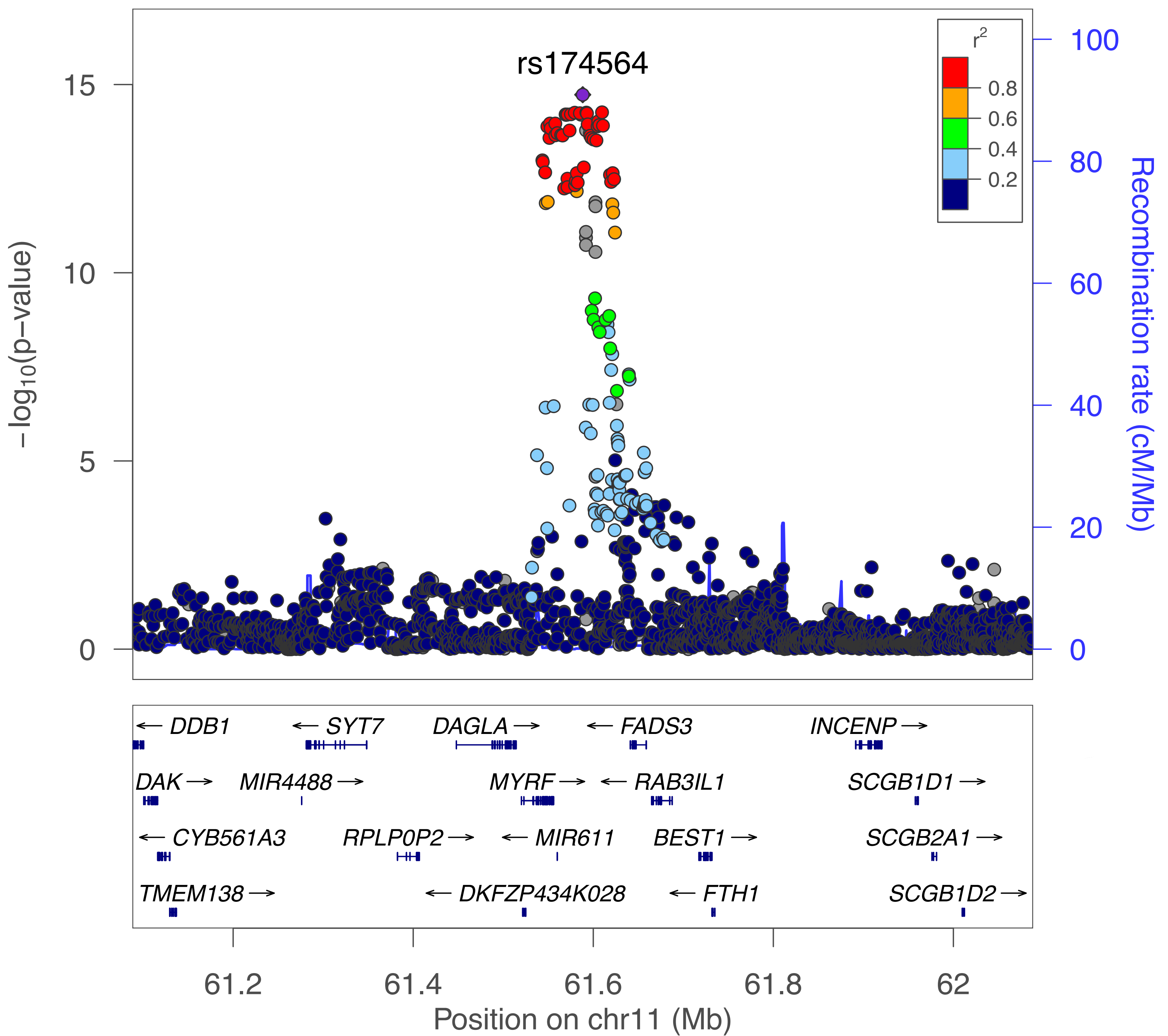

(xii) ACADS_ethylmalonate

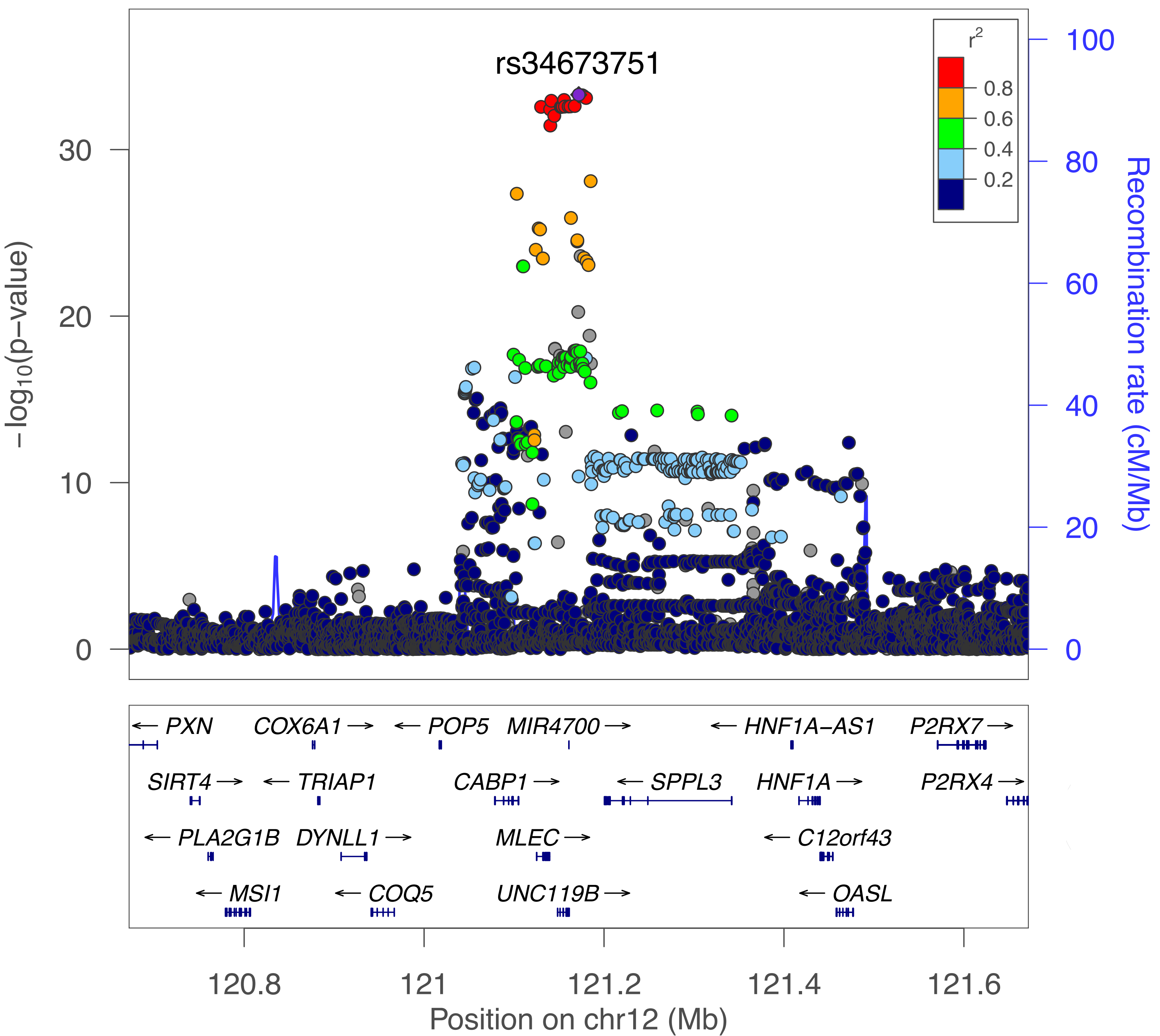

(xiii) TYMS/ENOSF1_ribonate

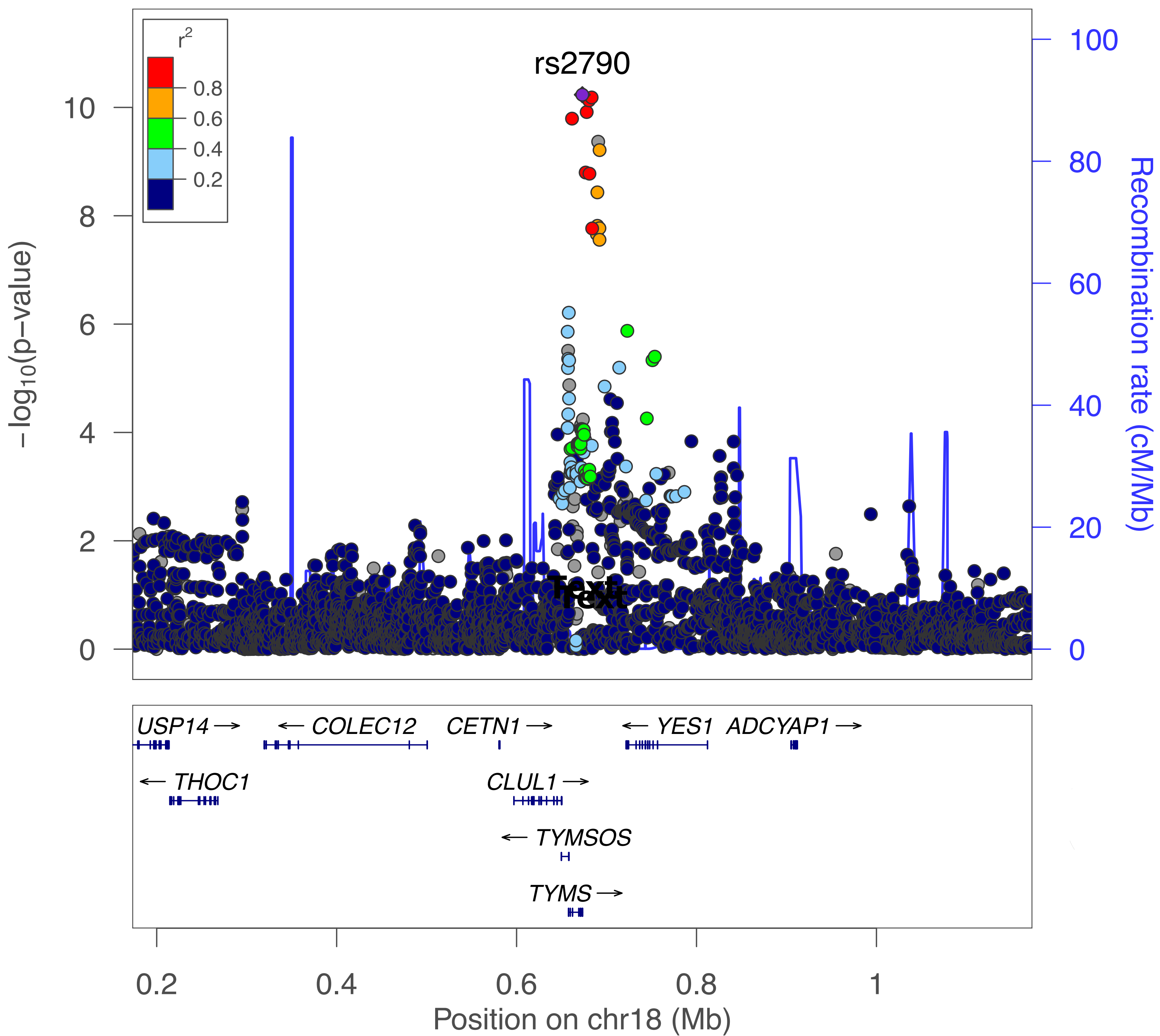

Supplement: FigureS4_ddz308 [file figures4_ddz308.pdf]
