# Supplementary figures and images for: Genome-wide scan identifies novel genetic loci regulating salivary metabolite levels
Source: Hum Mol Genet. 2020 Jan 21;29(5):864–75. doi: 10.1093/hmg/ddz308 (PMC7104674; doi:10.1093/hmg/ddz308)

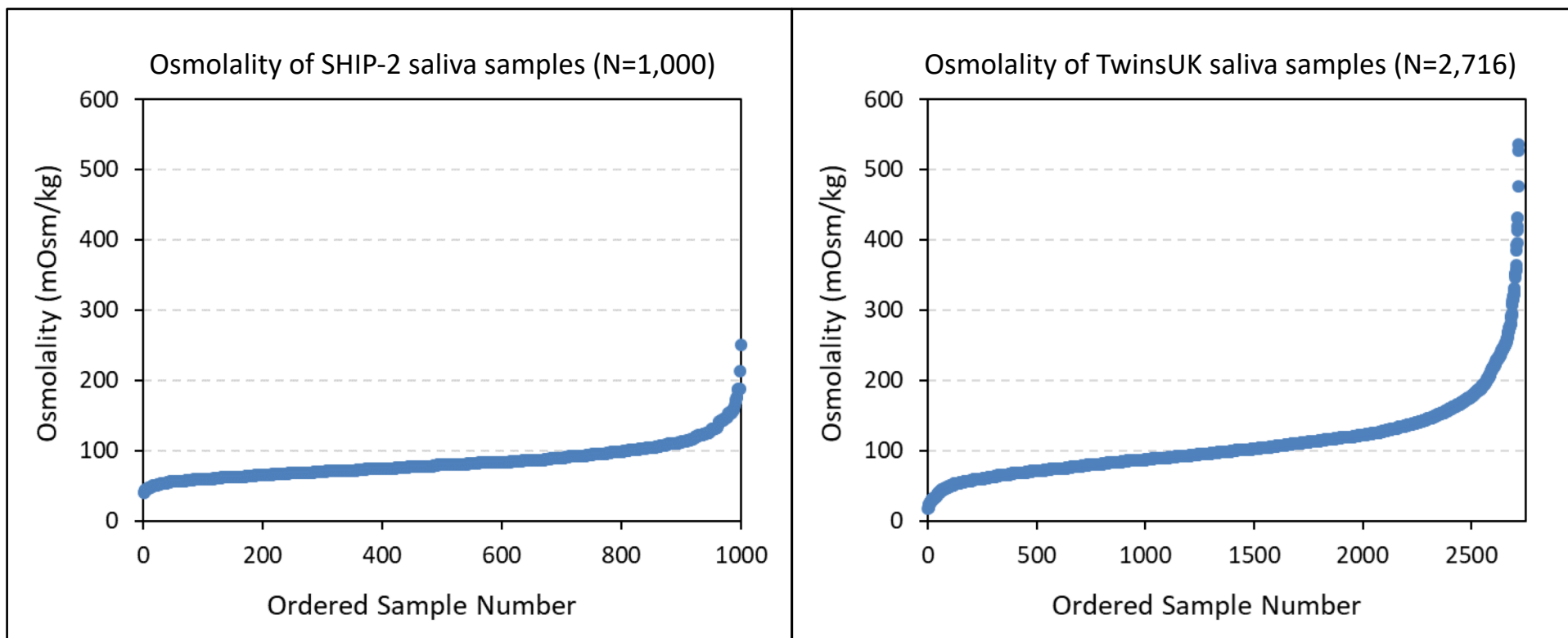

Supplement: FigureS5_ddz308 [file figures5_ddz308.pdf]
