# Supplementary material for: Genome-wide scan identifies novel genetic loci regulating salivary metabolite levels
Source: Hum Mol Genet. 2020 Jan 21;29(5):864–75. doi: 10.1093/hmg/ddz308 (PMC7104674; doi:10.1093/hmg/ddz308)
Supplement: FigureS6_ddz308 [file figures6_ddz308.pdf]

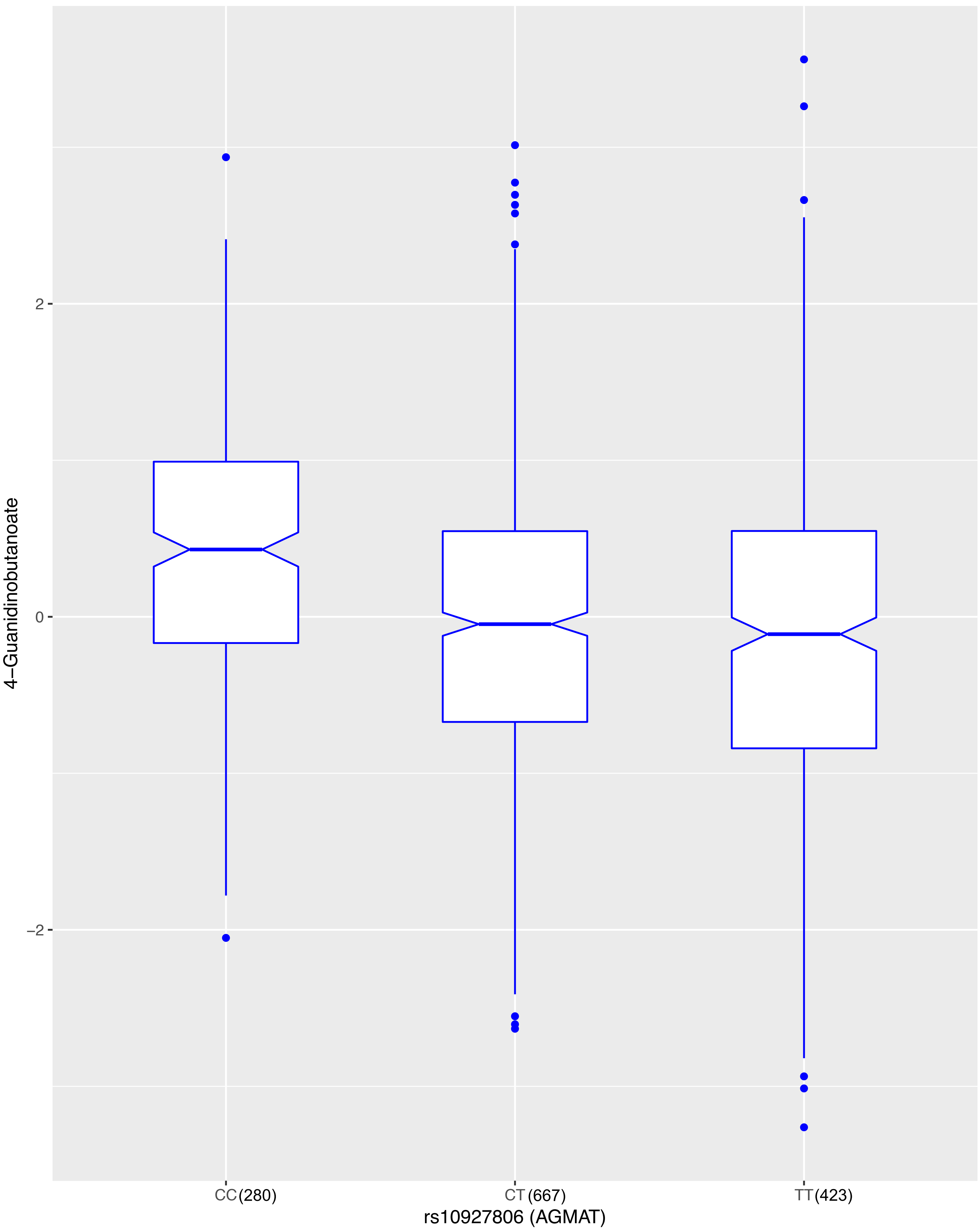

2
0
−2
4−Guanidinobutanoate
CC (280)
CT (667)
TT (423)
rs10927806 (AGMAT)

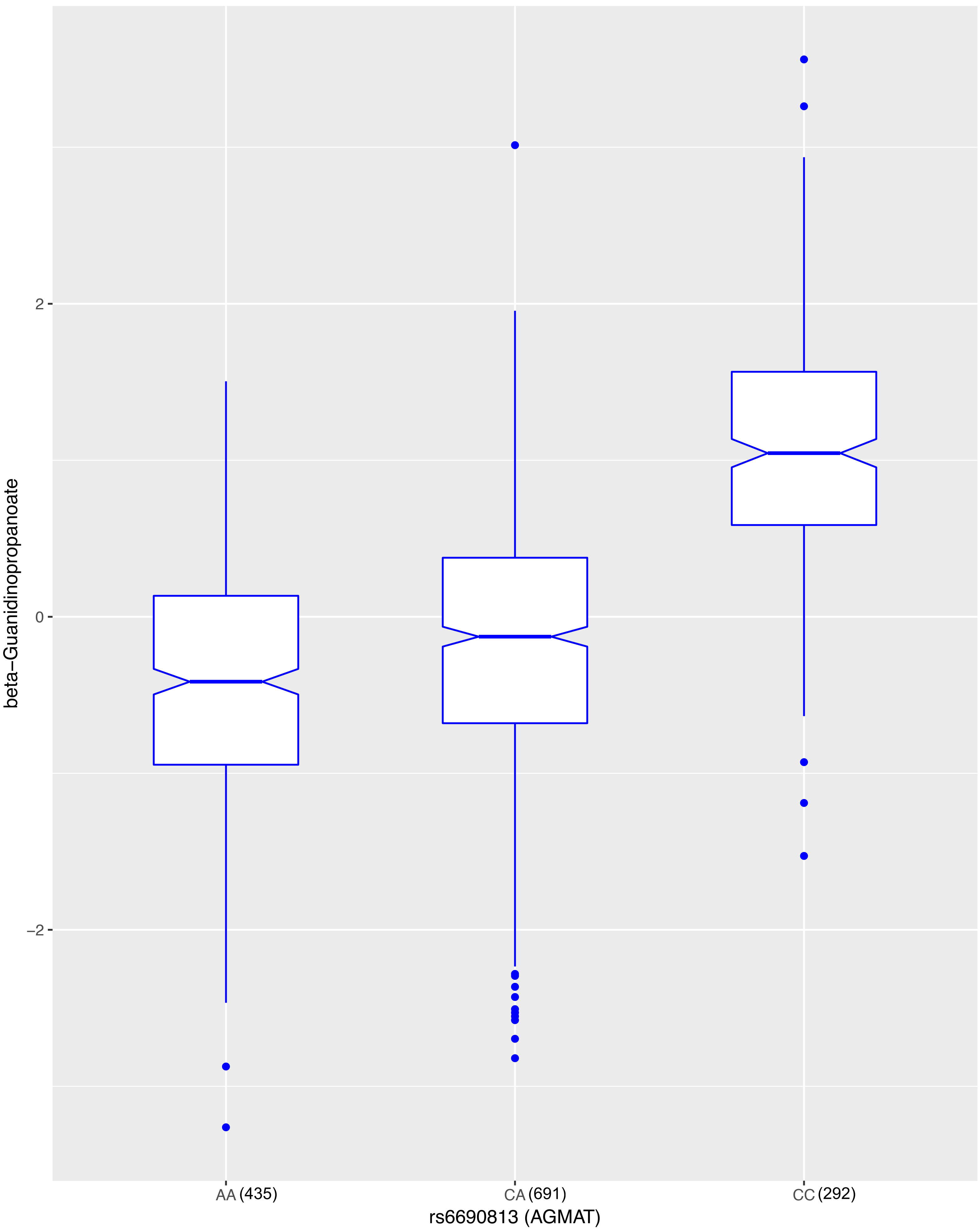
beta−Guanidinopropanoate
2
0
−2
AA (435)
CA (691)
CC (292)
rs6690813 (AGMAT)

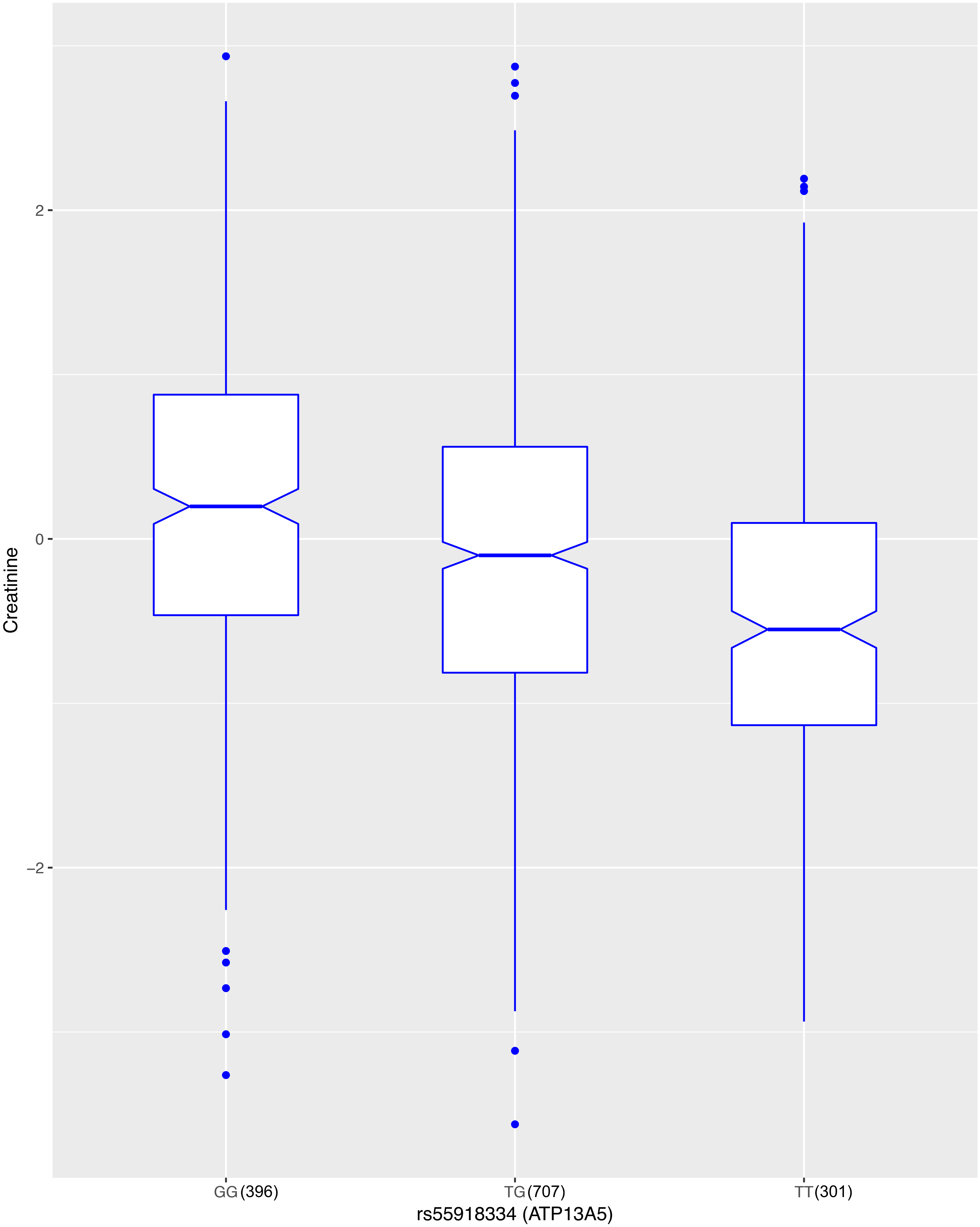

Creatinine
2
0
−2
GG (396)
TG (707)
TT (301)
rs55918334 (ATP13A5)

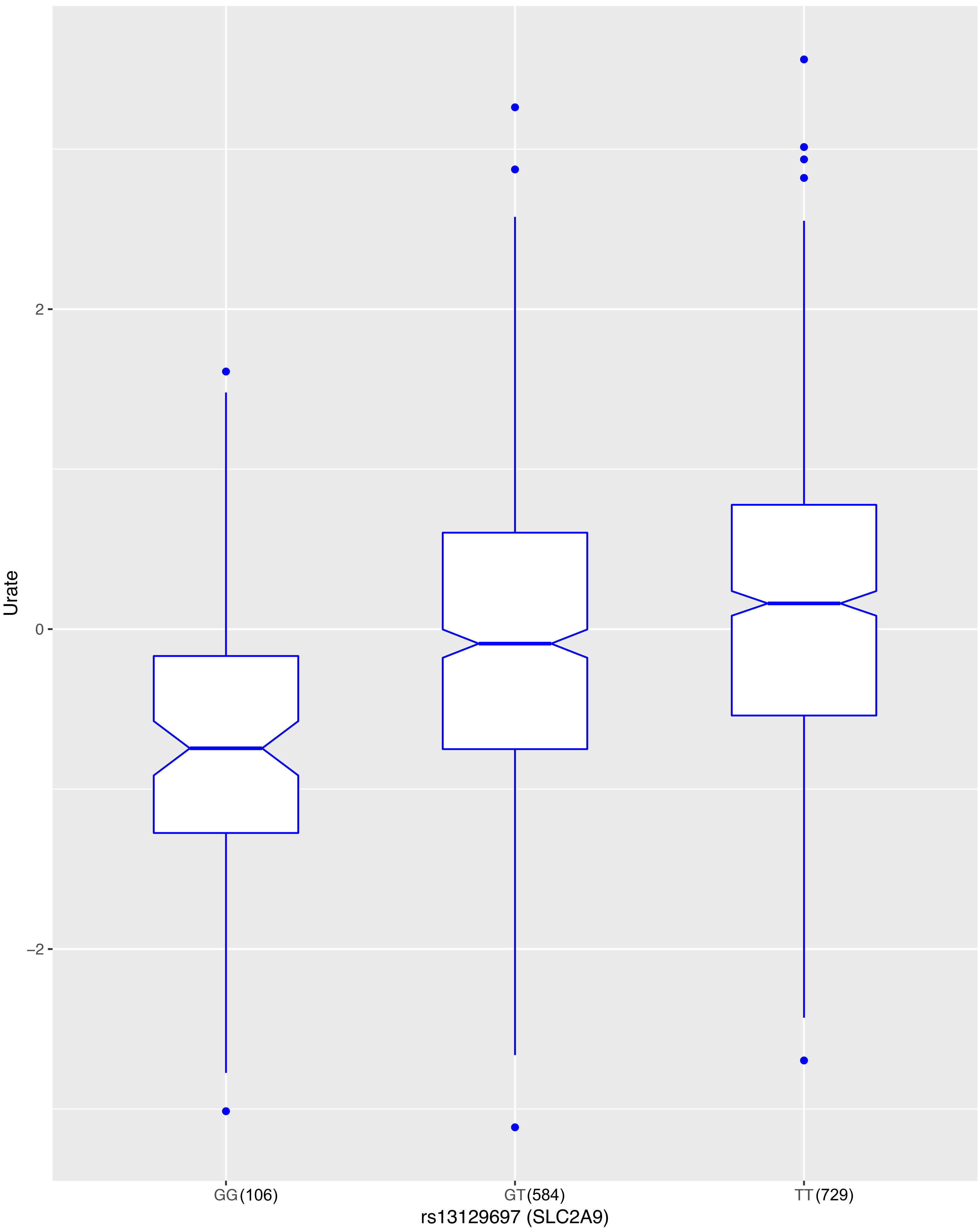
Urate
2
0
−2
GG (106)
GT (584)
TT (729)
rs13129697 (SLC2A9)

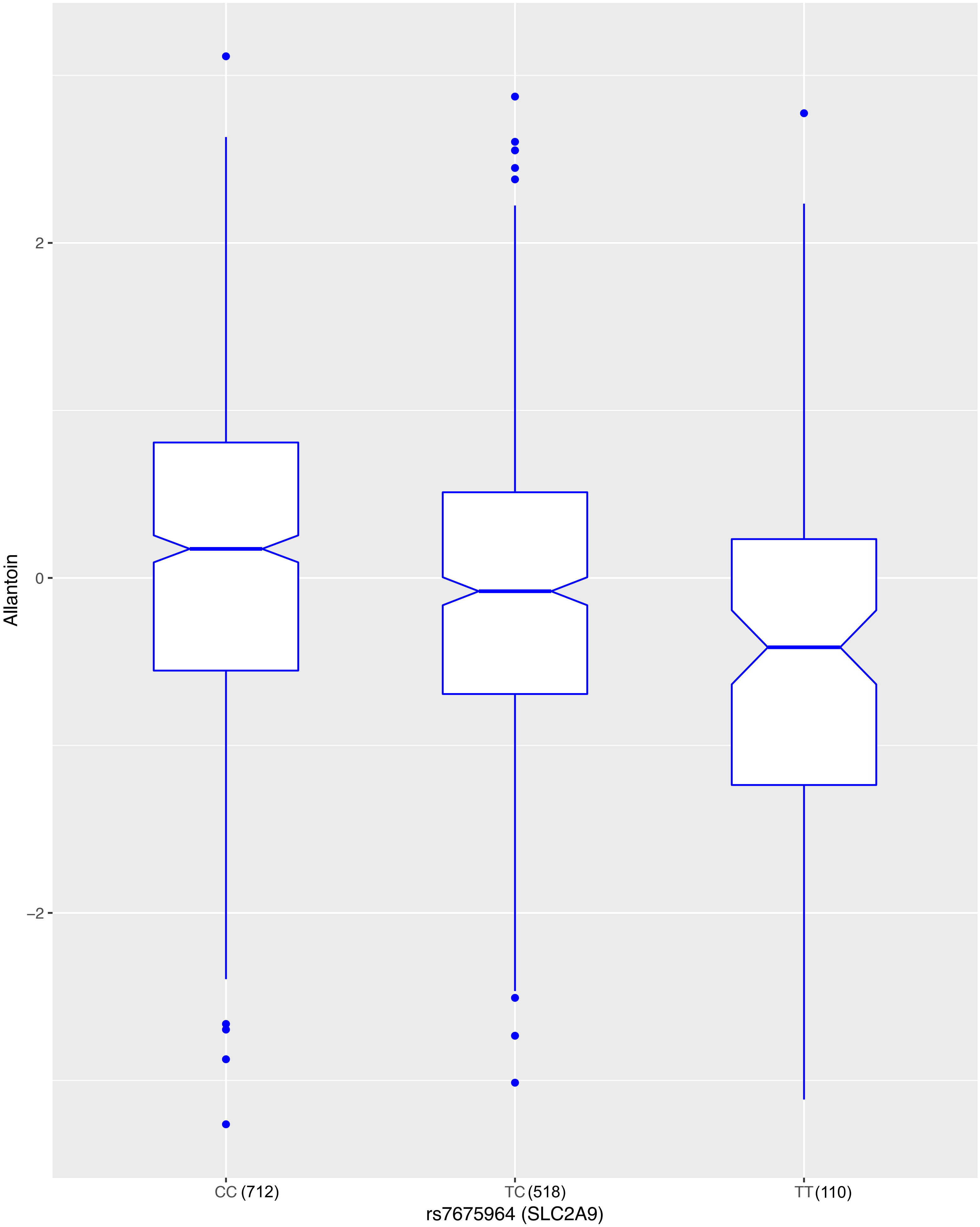

Allantoin
2
0
−2
CC (712)
TC (518)
TT (110)
rs7675964 (SLC2A9)

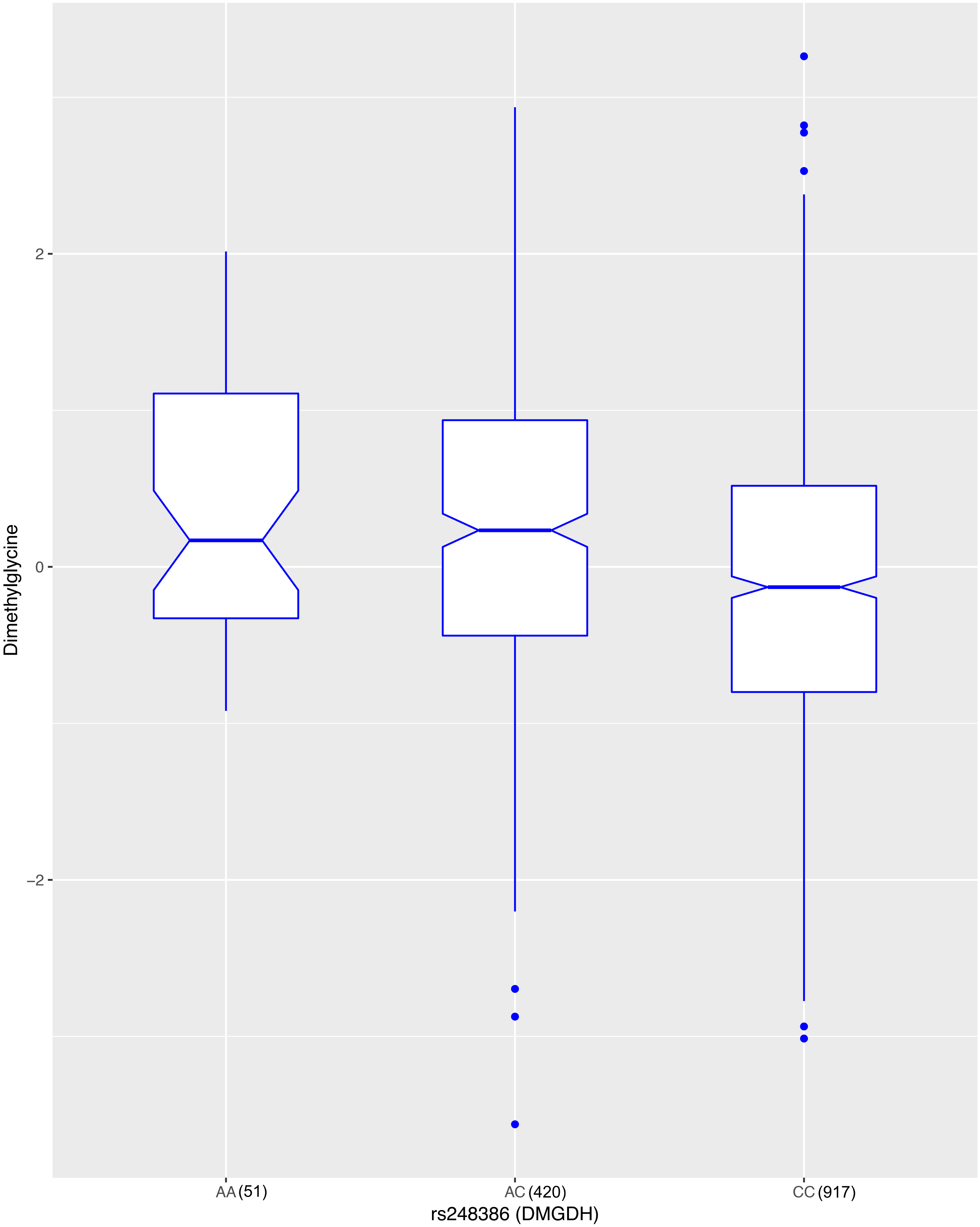

Dimethylglycine
2
0
−2
AA (51)
AC (420)
CC (917)
rs248386 (DMGDH)

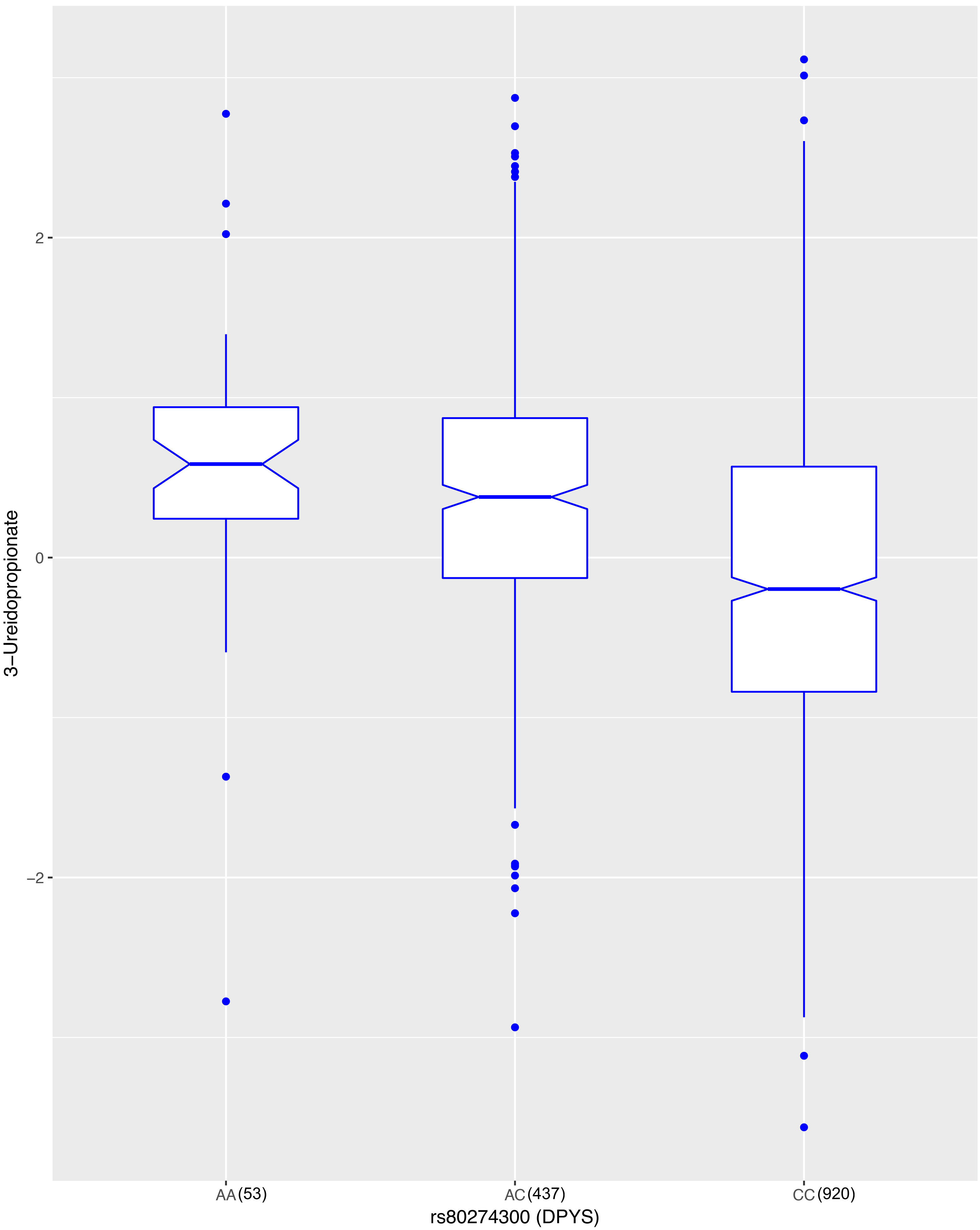

3–Ureidopropionate
2
0
–2
AA (53)
AC (437)
CC (920)
rs80274300 (DPYS)

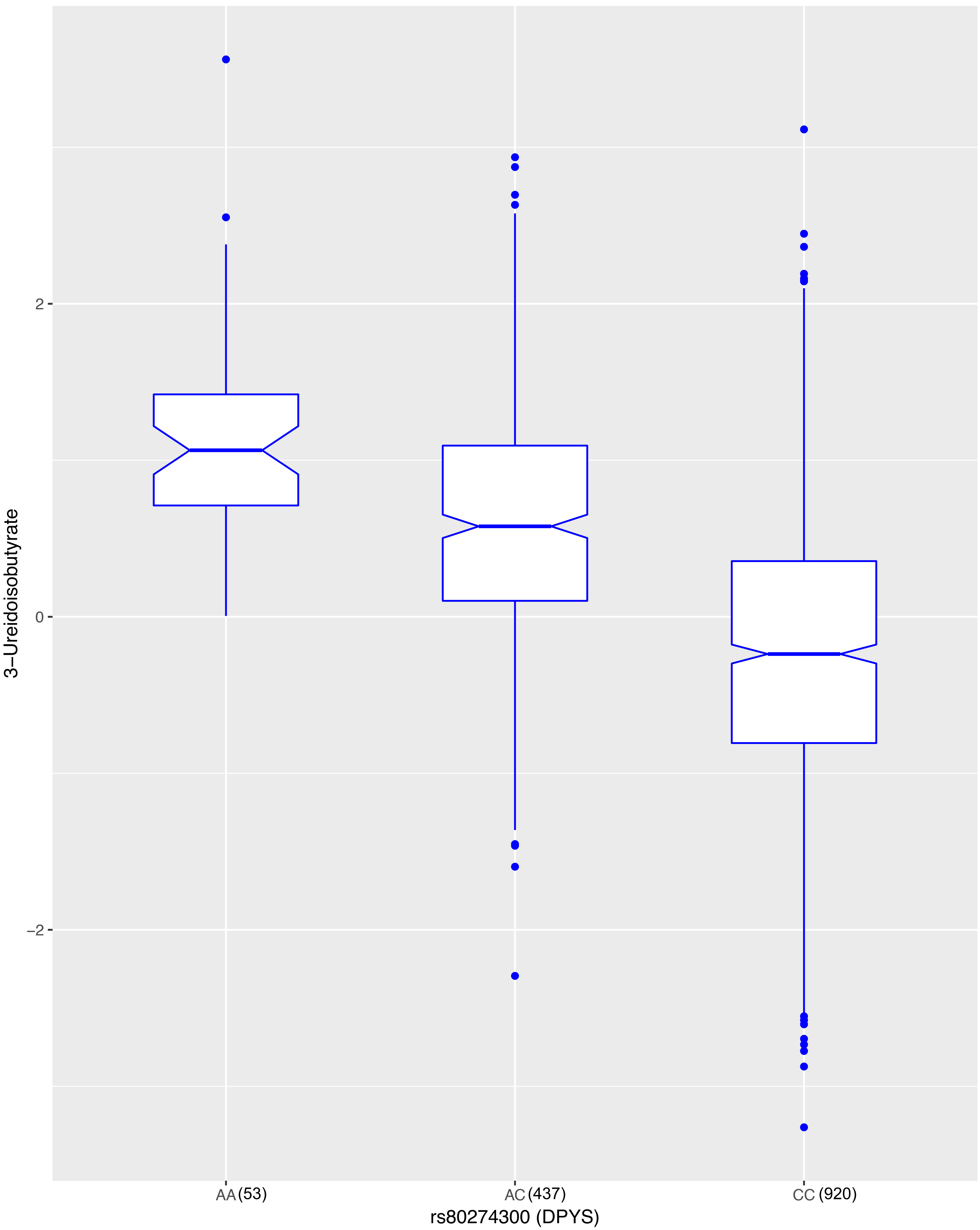

3−Ureidoisobutyrate
2
0
−2
AA (53)
AC (437)
CC (920)
rs80274300 (DPYS)

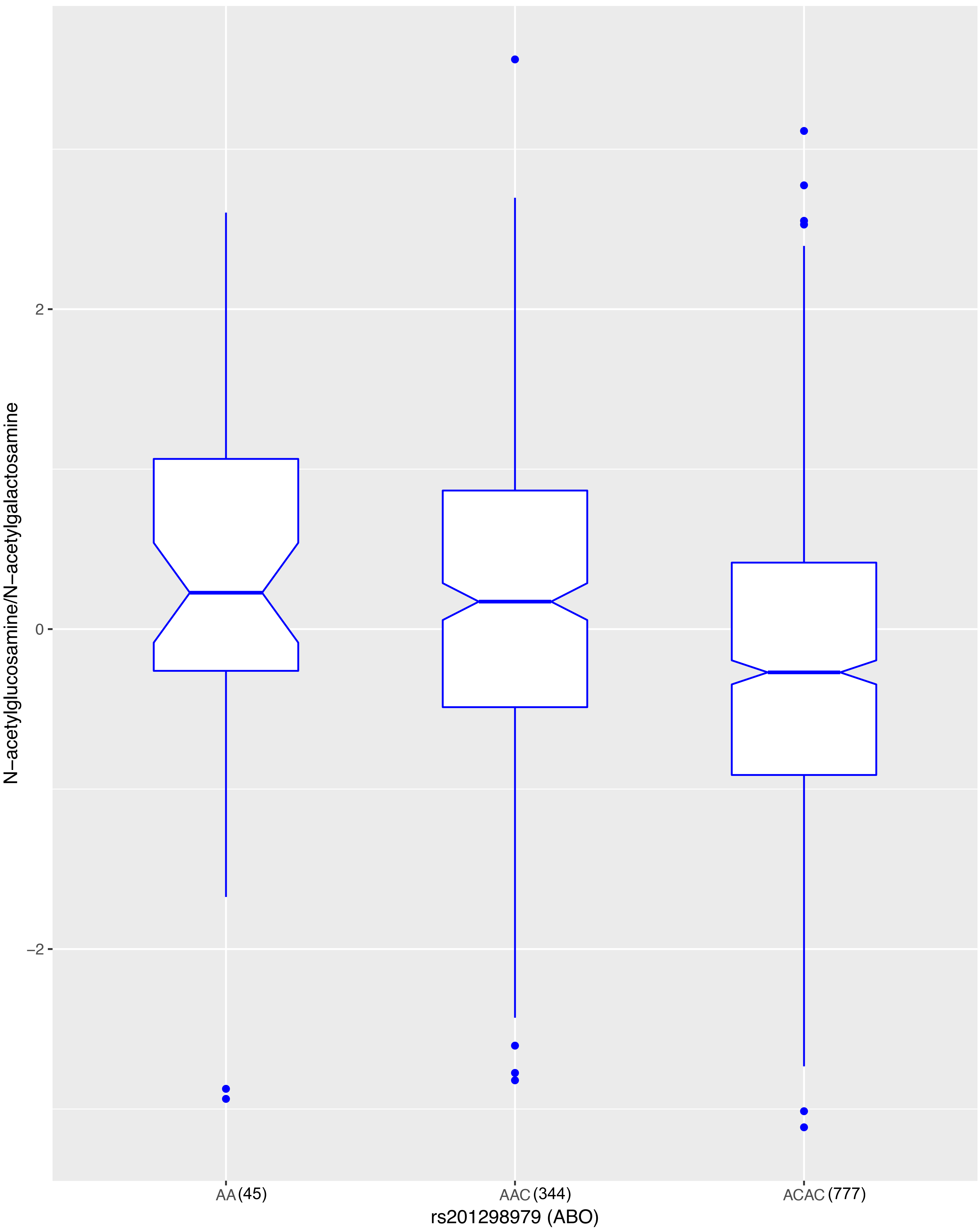

2
0
−2
N−acetylglucosamine/N−acetylgalactosamine
AA (45)
AAC (344)
ACAC (777)
rs201298979 (ABO)

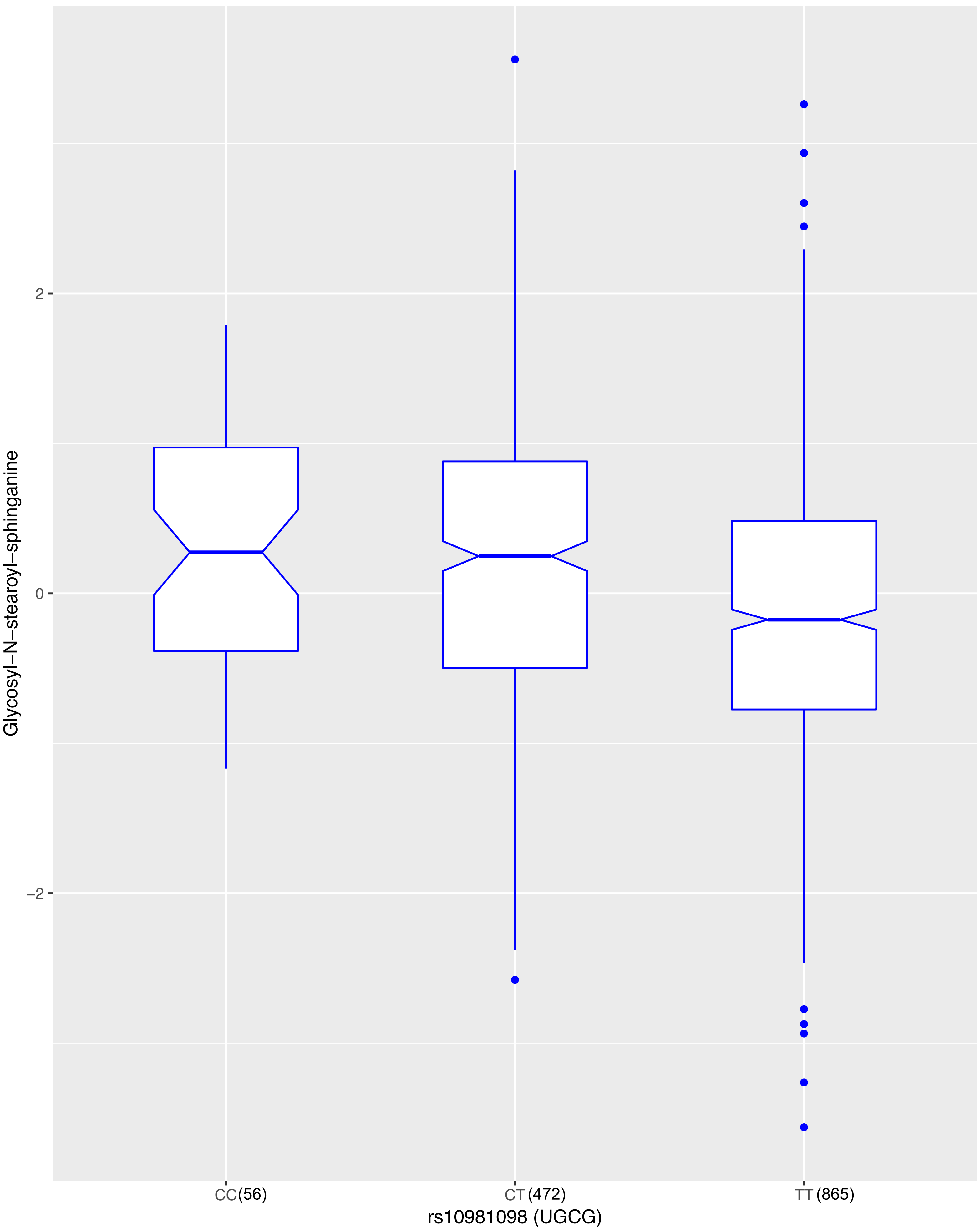

2
0
−2
Glycosyl−N−stearoyl−sphinganine
CC (56)
CT (472)
TT (865)
rs10981098 (UGCG)

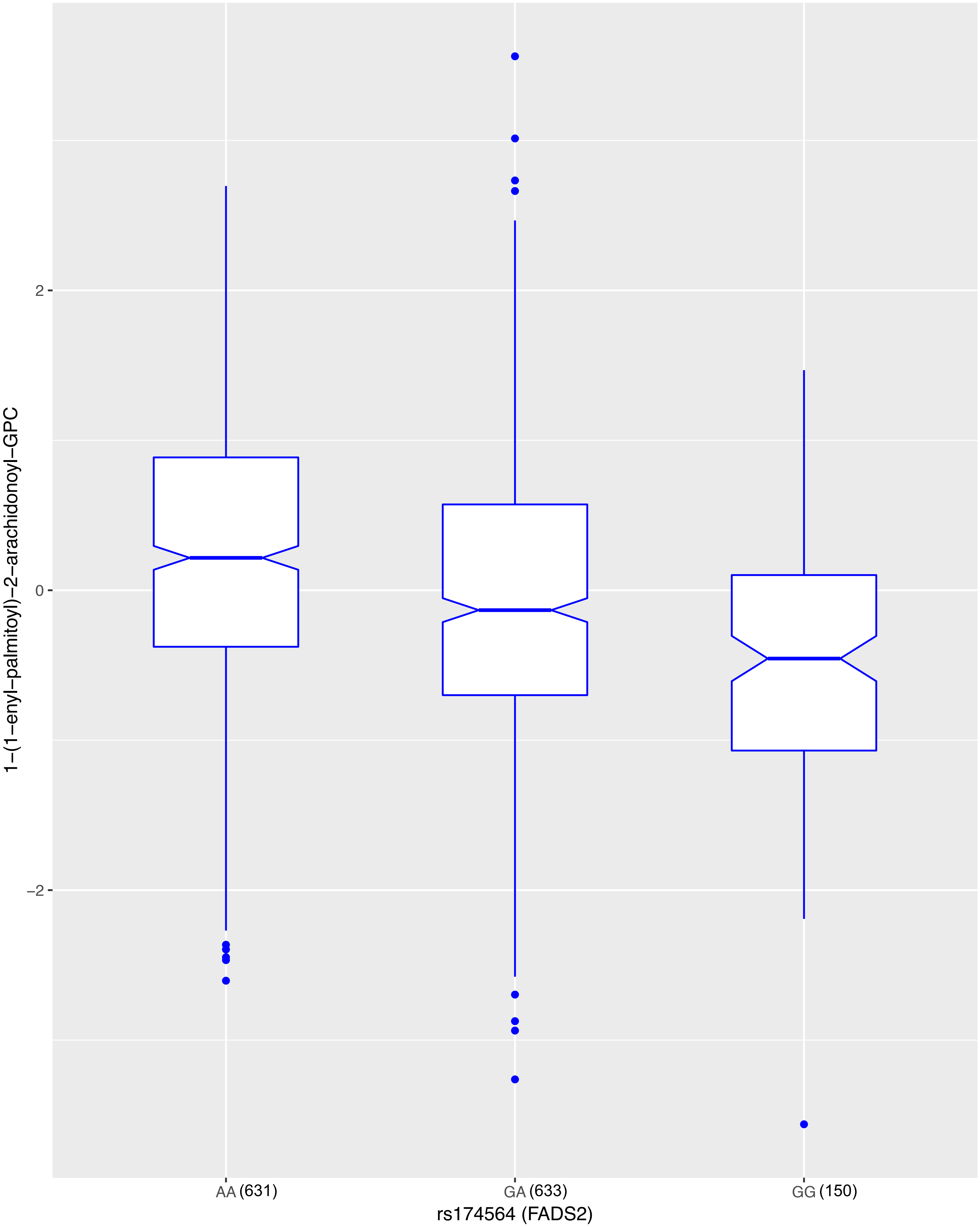

2
0
−2
1−(1−enyl−palmitoyl)−2−arachidonoyl−GPC
AA (631)
GA (633)
GG (150)
rs174564 (FADS2)

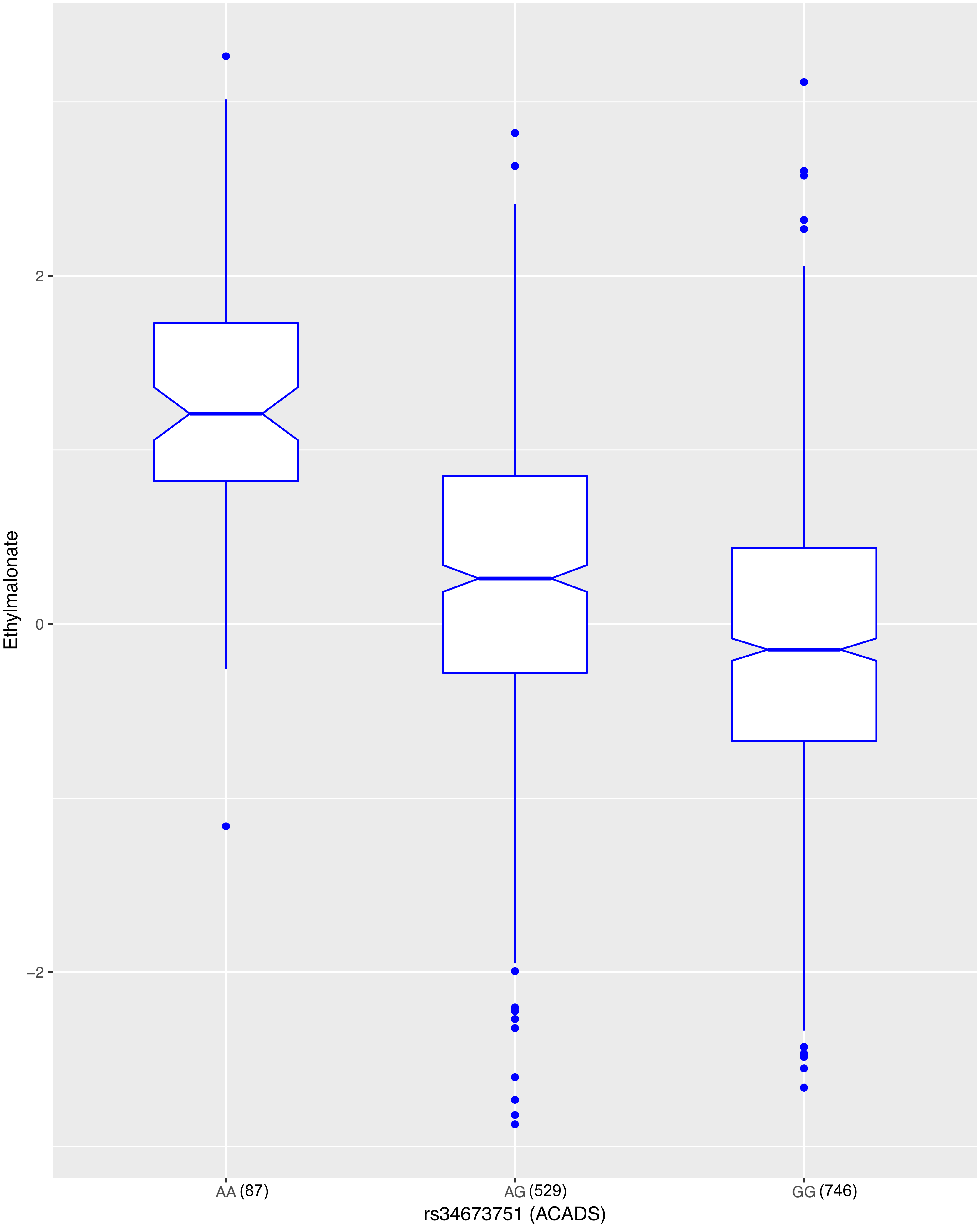
Ethylmalonate
2
0
−2
AA (87)
AG (529)
GG (746)
rs34673751 (ACADS)

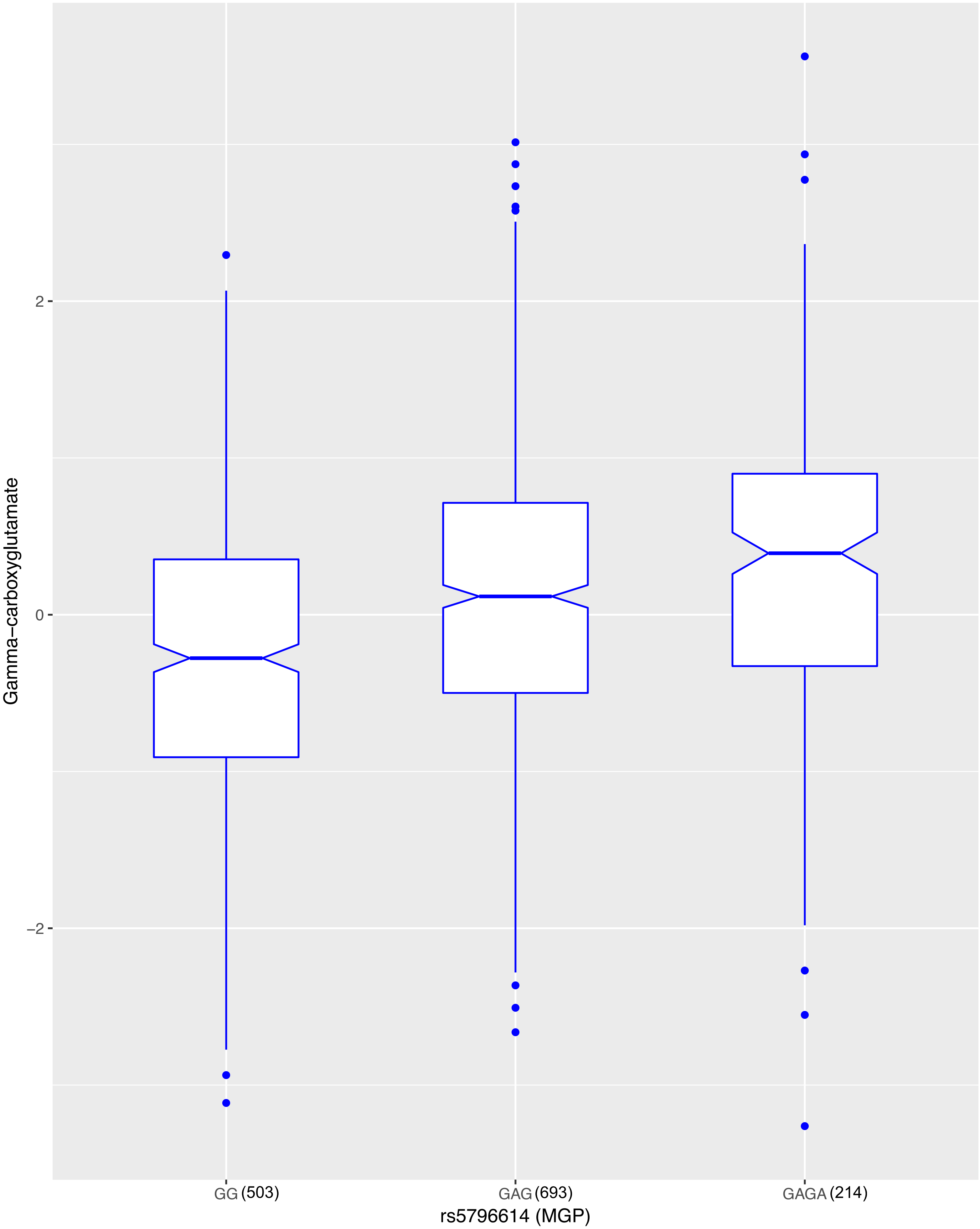
Gamma−carboxyglutamate
2
0
−2
GG (503)
GAG (693)
GAGA (214)
rs5796614 (MGP)

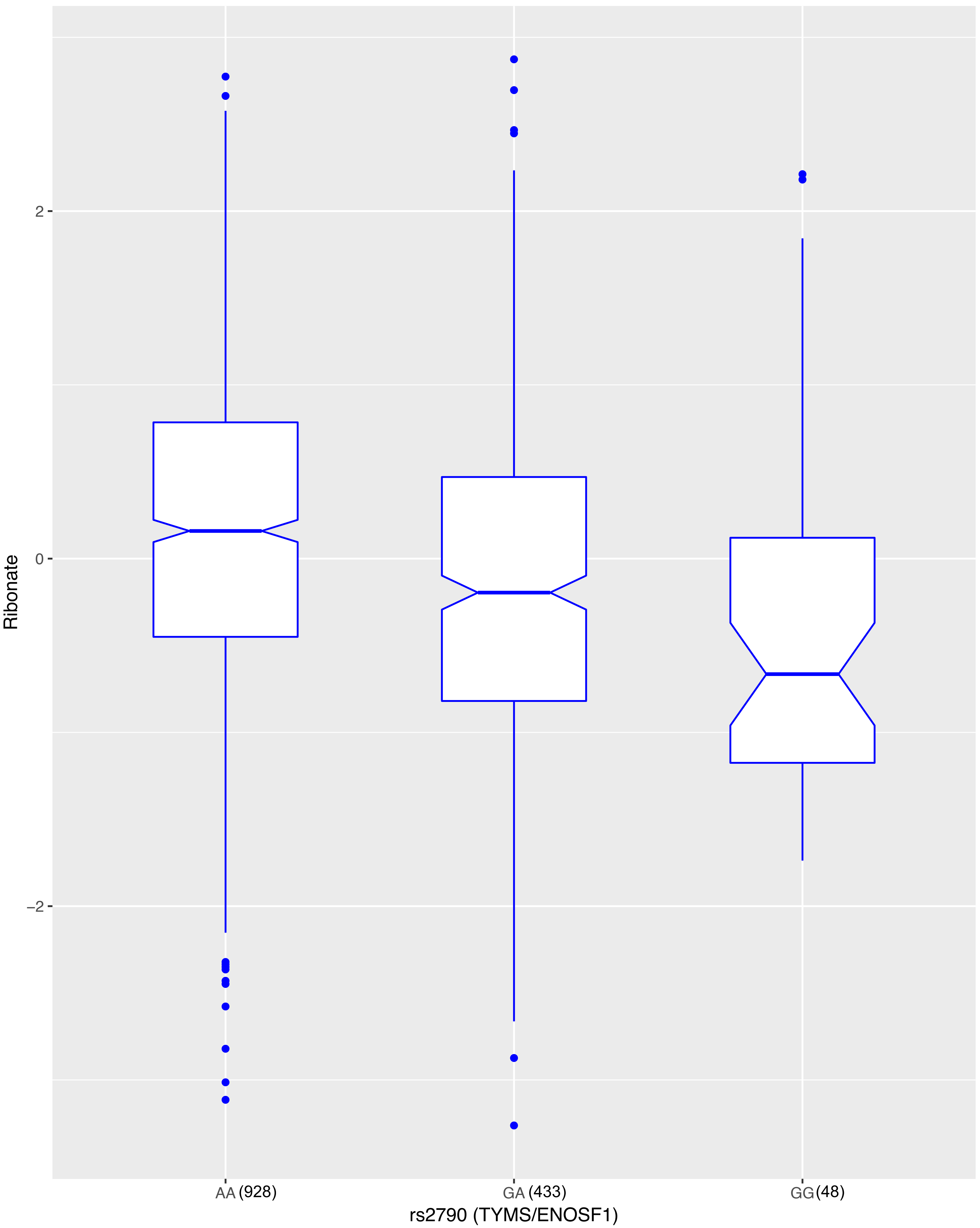

Ribonate
2
0
−2
AA (928)
GA (433)
GG (48)
rs2790 (TYMS/ENOSF1)
